# A microfluidics-based *in vitro* model of the gastrointestinal human–microbe interface

**DOI:** 10.1038/ncomms11535

**Published:** 2016-05-11

**Authors:** Pranjul Shah, Joëlle V. Fritz, Enrico Glaab, Mahesh S. Desai, Kacy Greenhalgh, Audrey Frachet, Magdalena Niegowska, Matthew Estes, Christian Jäger, Carole Seguin-Devaux, Frederic Zenhausern, Paul Wilmes

**Affiliations:** 1 Luxembourg Centre for Systems Biomedicine, University of Luxembourg, 6 avenue du Swing, Belvaux L-4367, Luxembourg; 2 Center for Applied Nanobioscience and Medicine, University of Arizona, 145S 79th Street, Suite 16, Chandler, Arizona 85226, USA; 3 Department of Infection and Immunity, Luxembourg Institute of Health, 29 rue Henri Koch, Esch-sur-Alzette L-4354, Luxembourg; 4 Department of Basic Medical Sciences, University of Arizona, 425N. 5th Street, Phoenix, Arizona 85004, USA

## Abstract

Changes in the human gastrointestinal microbiome are associated with several diseases. To infer causality, experiments in representative models are essential, but widely used animal models exhibit limitations. Here we present a modular, microfluidics-based model (HuMiX, human–microbial crosstalk), which allows co-culture of human and microbial cells under conditions representative of the gastrointestinal human–microbe interface. We demonstrate the ability of HuMiX to recapitulate *in vivo* transcriptional, metabolic and immunological responses in human intestinal epithelial cells following their co-culture with the commensal *Lactobacillus rhamnosus* GG (LGG) grown under anaerobic conditions. In addition, we show that the co-culture of human epithelial cells with the obligate anaerobe *Bacteroides caccae* and LGG results in a transcriptional response, which is distinct from that of a co-culture solely comprising LGG. HuMiX facilitates investigations of host–microbe molecular interactions and provides insights into a range of fundamental research questions linking the gastrointestinal microbiome to human health and disease.

## Abstract (web summary)

Research on the interactions between the gut microbiota and human cells would greatly benefit from improved *in vitro* models. Here, Shah *et al*. present a modular microfluidics-based model that allows co-culture of human and microbial cells followed by 'omic' molecular analyses of the two cell contingents.

## Graphical abstract

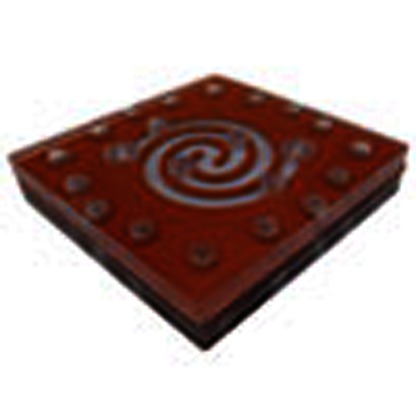

The human microbiome is emerging as a key player governing human health and disease^1^,^2^. Recent high-resolution molecular analyses have linked microbial community disequilibria (dysbiosis), primarily in the gastrointestinal tract (GIT), to several idiopathic diseases, including diabetes^3^, obesity^4^, inflammatory bowel disease^5^, cancer^6^ and, most recently, neurodegenerative diseases^7^. However, a detailed understanding of the fundamental molecular mechanisms underlying host–microbe interactions and their potential impact on immune regulation, drug metabolism, nutrition and infection remain largely elusive^8^,^9^. More specifically, patterns of association between distinct microorganisms, their traits and disease states resolved using ‘meta-omics' do not allow direct causal inference, and thus experimental validation is essential^10^. For this, robust experimental models that allow the systematic manipulation of variables are required to test the multitude of hypotheses that arise from the generated high-dimensional data sets^10^. Animal models used in human microbiome research are physiologically not representative^11^. *In vitro* models that mimic microbial processes along the GIT allow the simulation of luminal microbial communities^12^,^13^,^14^ and/or mucus-adherent microbiota^15^,^16^, but typically do not include provisions for assessing human host responses.

Host responses to GIT microbiota have traditionally been assessed following the exposure of cultured human cells to bacteria-free supernatants^17^ or through short-term direct-contact co-cultures involving, for example, Transwell systems^18^, microcarrier beads^19^ or mouse gut organoid models^20^. Recent advances in multi-layer microfluidics have led to the development of a gut-on-a-chip model that includes a provision for peristalsis^21^ and that has been used to study intestinal inflammation on a chip^22^. These human–microbial co-culture approaches are, however, limited in their scope because they only allow experiments with commensal and/or mutualistic microorganisms growing under aerobic conditions^21^,^22^. To overcome these limitations, the recently introduced host–microbiota interaction (HMI) module, which interfaces with the *in vitro* simulator of the human intestinal microbial ecosystem model, incorporates a semi-permeable membrane between co-cultured human enterocytes and bacteria^23^. Through inclusion of a partitioning membrane between the human and microbial culture chambers, the HMI module allows the co-culture of intestinal cells with complex microbial communities under microaerophilic conditions^23^. This two-chamber design requires intermittent perfusion of the human cell culture medium to the apical surface of the epithelial cells, which is not representative of the continuous supply of nutrients to the basal membrane seen *in vivo*^24^,^25^,^26^. The lack of modularity makes it difficult to include additional cell types of relevance to the GIT in the HMI module, for example, immune cells. Furthermore, it prevents the extraction of biomolecular fractions from the individual co-cultured cell contingents following specific experimental regimes and thereby renders the HMI module incompatible with downstream high-resolution molecular analyses. Although the HMI module currently is the most representative *in vitro* model of gastrointestinal host–microbial interactions, there still remains an unmet need for a modular, representative *in vitro* model of the gastrointestinal human–microbe interface.

Here we present a modular microfluidics-based human–microbial co-culture model, HuMiX, which overcomes the majority of the limitations of existing *in vitro* models and allows the partitioned yet proximal co-culture of representative human and microbial cells followed by downstream molecular analyses of the individual cell contingents. More specifically, we demonstrate the viable co-culture of differentiated human epithelial cells (Caco-2) with either a facultative anaerobe, *Lactobacillus rhamnosus* GG (LGG), grown solely under aerobic or anaerobic conditions, or grown in combination with an obligate anaerobe, *Bacteroides caccae*, under anaerobic conditions. Co-culture experiments were followed by detailed molecular analyses of the effects of the induced co-cultures on the physiology of human and bacterial cells. Comparison of our results with published *in vitro* and *in vivo* data sets demonstrates the ability of HuMiX to representatively mimic the gastrointestinal human–microbe interface.

## Results and Discussion

### Design and characterisation of the HuMiX model

**Figure 1:**
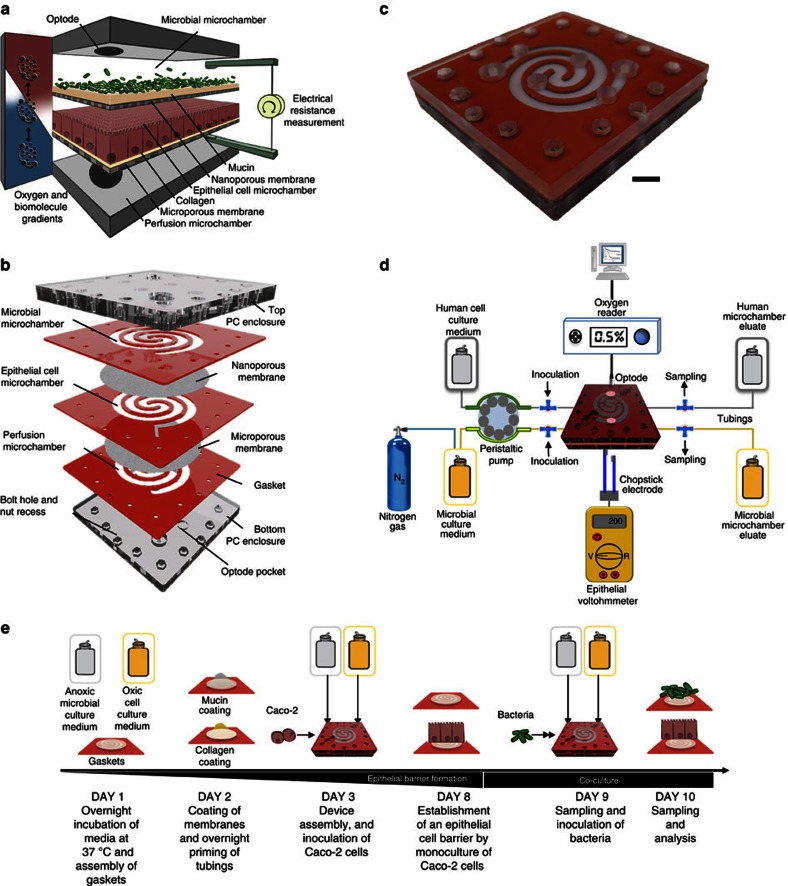
The HuMiX model. (**a**) Conceptual diagram of the HuMiX model for the representative co-culture of human epithelial cells with gastrointestinal microbiota. (**b**) Annotated exploded view of the HuMiX device. The device is composed of a modular stacked assembly of elastomeric gaskets (thickness: 700 μm) sandwiched between two polycarbonate (PC) enclosures, and each gasket defines a distinct spiral-shaped microchannel with the following characteristics: length of 200 mm, width of 4 mm and height of 0.5 mm, amounting to a total volume of 400 μl per channel. Semi-permeable membranes affixed to the elastomeric gaskets demarcate the channels. The pore sizes of the membranes were chosen for their intended functionality. A microporous membrane (pore diameter of 1 μm), which allows diffusion-dominant perfusion to the human cells, is used to partition the perfusion and human microchambers. A nanoporous membrane (pore diameter of 50 nm) partitions the human and microbial microchambers to prevent the infiltration of microorganisms, including viruses, into the human microchamber. (**c**) Photograph of the assembled HuMiX device (scale bar, 1 cm). (**d**) Diagram of the experimental set-up of the HuMiX model with provisions for the perfusion of dedicated oxic and anoxic culture media as well as the monitoring of the oxygen concentration and transepithelial electrical resistance. The oxygen concentration in the anoxic medium is maintained at 0.1% by continuously bubbling the medium with dinitrogen gas. (**e**) Diagrammatic overview of the HuMiX co-culture protocol.

To overcome the limitations of existing *in vitro* models^10^,^23^, we developed a modular microfluidics-based device, which allows the establishment of a model of the gastrointestinal human–microbe interface, named HuMiX (human-microbial crosstalk) (Fig. 1a–c). The device consists of three co-laminar microchannels: a medium perfusion microchamber (henceforth referred to as the ‘perfusion microchamber'), a human epithelial cell culture microchamber (henceforth referred to as the ‘human microchamber') and a microbial culture microchamber (henceforth referred to as the ‘microbial microchamber'; Fig. 1a,b; Supplementary Fig. 1a,b). Each microchamber has a dedicated inlet and outlet for the inoculation of cells as well as for the precise control of physicochemical parameters through the perfusion of laminar streams of dedicated culture media (Fig. 1d,e). Dedicated outlets provide means to collect eluates from the individual chambers for downstream characterisation (Fig. 1d; Supplementary Fig. 1a,b). By juxtaposing the human and microbial cell contingents at a distance of 0.5-1 mm across a separatory nanoporous membrane, the HuMiX model is representative of a healthy intact epithelial barrier^10^ (Supplementary Note 1). Furthermore, the model integrates oxygen sensors (optodes) for the real-time monitoring of the dissolved oxygen concentrations within the device (Fig. 1a,b,d; Supplementary Fig. 1c). Given the challenges associated with measuring transepithelial electrical resistance (TEER) on a chip^27^, a specially designed version of HuMiX, which allows the insertion of a commercial chopstick style electrode (STX2; Millipore), was fabricated to monitor TEER for the characterisation of cell growth and differentiation within the device (Fig. 1d; Supplementary Fig. 1d).

**Figure 2:**
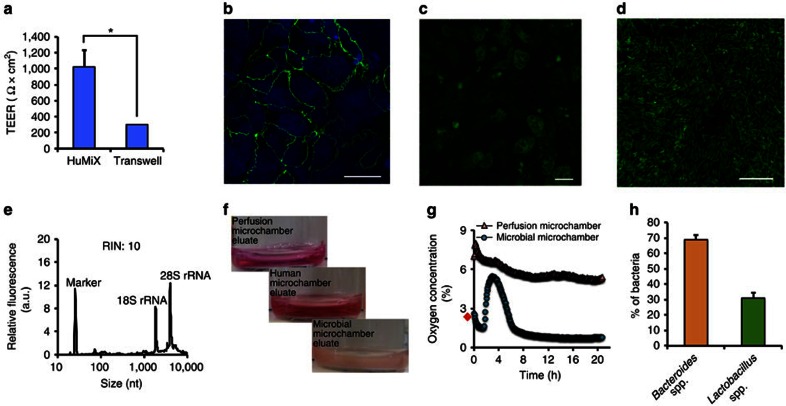
*In vitro* co-culture of human and microbial cells inside the HuMiX device. (**a**) Characterisation of epithelial cell monolayer formation in HuMiX in comparison with the standard Transwell system. In both cases, the transepithelial electrical resistance (TEER) was determined on 7-day-old Caco-2 cell layers using standard chopstick electrodes. The error bars indicate the s.e.m. (*n*=3). * Indicates a statistically significant difference (paired Student's *t*-test, *P*<0.05). (**b**) Immunofluorescent microscopic observation of the tight junction protein occludin (green) in Caco-2 cells following 24 h of co-culture with LGG grown under anaerobic conditions. The cell nuclei are stained with 4,6-diamidino-2-phenylindole and appear in blue. (**c**,**d**) Viability assessment of Caco-2 cells and LGG at 24 h post co-culture, respectively. The cells were stained using a live–dead stain and observed using a fluorescence microscope. The live cells appear in green, whereas the dead cells appear in red. The collagen-coated microporous membrane does support the attachment and proliferation of the Caco-2 cells, whereas the mucin-coated nanoporous membrane provides a surface for the attachment and subsequent proliferation of the bacteria. (**e**) Representative electropherogram of an RNA fraction obtained from the Caco-2 cells co-cultured in HuMiX. The RNA Integrity Number (RIN) is provided. (**f**) Sampled eluates from the HuMiX device following a 24 h co-culture with LGG. (**g**) Oxygen concentration profiles within the perfusion and microbial microchambers upon initiation of the co-culture with LGG. ⋄indicates the pre-inoculation oxygen concentration of 2.6% in the microbial microchamber. (**h**) The relative abundances (in %) of *Lactobacillus* spp. and *Bacteroides* spp. following 24 h of co-culture with Caco-2 cells determined by 16S rRNA gene amplicon sequencing (*n*=4). Scale bars, 10 μm (**b**–**d**).

Following the conceptualisation and engineering of the HuMiX model (Supplementary Note 1), we developed an optimised protocol for the co-culture of human epithelial cells with gastrointestinal microbes (Fig. 1e). The human cell line and bacterial isolates used for the co-culture experiments were originally obtained from the human large intestine and, together with the physical characteristics of the model (Supplementary Note 1), allowed the assembly of a model representing the human–microbe interface of the human colon. Nonetheless, given the modularity of the device and the flexibility of its set-up, other sections of the human GIT may also be modelled following appropriate modifications to the presented model (Supplementary Note 1). The protocol includes an extensive sterilisation and handling procedure that enables the culture of human epithelial cells (Caco-2) in antibiotic-free DMEM medium to allow their subsequent co-culture with bacteria in HuMiX. The Caco-2 cell line was chosen because it represents the most widely used model for the human gastrointestinal epithelial barrier, as it exhibits essential functional and physiological traits of the intestinal epithelium^25^,^28^. The differentiation of the epithelial cells was evaluated by measuring TEER of the Caco-2 cell monolayer (Fig. 2a) and through microscopic observation of the expression of the tight junction protein occludin (Fig. 2b).

Following the establishment of differentiated Caco-2 cell monolayers, we initiated co-cultures of these cells with LGG grown in anoxic DMEM medium (Supplementary Fig. 2a). LGG of the phylum Firmicutes was chosen, as it represents a commensal facultative anaerobic bacterium originally isolated from the human GIT^29^,^30^,^31^. Importantly, extensive data exist on its physiological impacts on mammalian mucosal tissues *in vivo*^32^,^33^,^34^. The developed co-culture protocol (Fig. 1e) first results in the establishment and maintenance of an epithelial cell monolayer. The Caco-2 cells adhere to the collagen-coated microporous membrane (Fig. 1a,e; Supplementary Note 1), proliferate and differentiate into confluent cell monolayers that form tight junctions between adjacent cells (Fig. 2a,b). The diffusion-based perfusion of the cell culture medium to the basal side of the Caco-2 cells through the microporous membrane mimics the intestinal blood supply and provides shear-free conditions accelerating the growth of the human cells^35^.

Co-culture with LGG was initiated after 7 days of epithelial cell culture (day 9 of the HuMiX co-culture protocol; Figs 1e and 2a,b). This first involved the introduction of anaerobically grown LGG cell suspensions into the microbial microchamber through the port on a three-way connector (Fig. 1d).

Following the co-culture, the modular device architecture allows access to individual cell contingents on disassembly, whereby one half of each of the cell contingents can be used for microscopic evaluation and the other half can be used for the extraction of intracellular biomolecules (DNA, RNA, proteins and metabolites) for subsequent high-resolution molecular analyses^36^. The viability of the co-cultured contingents was determined via live–dead staining and subsequent fluorescence microscopy, demonstrating that no apparent cytotoxic effects were induced in either cell contingent following their co-culture (Fig. 2c,d). RNA electropherograms confirmed that high-quality biomolecular fractions were obtained from the individual co-cultured contingents (Fig. 2e).

Due to the laminar flow profiles within the microchambers, eluate samples (Fig. 2f) can be recovered from each microchamber, thereby providing a means to continually monitor the effects of the co-culture on the individual co-cultured cell contingents through various analyses, such as the use of cytokine assays and metabolomic profiling. Visible differences in the eluates from the three proximal microchambers support the notion of distinct microenvironments in each of the microchambers (Fig. 2f).

Integrated oxygen sensors (optodes) allow continuous monitoring of the dissolved oxygen concentrations in the perfusion and microbial microchambers (Fig. 1e; Supplementary Fig. 1c). The simultaneous perfusion of oxic (21% dissolved O_2_) and anoxic (0.1% O_2_) media through the perfusion microchamber and the microbial microchamber, respectively, allowed the establishment and maintenance of an oxygen gradient representative of the *in vivo* situation (Fig. 2g). The measured dissolved oxygen concentrations in the perfusion microchamber stabilised to 5.43±0.137% for the final 12 h of co-culture between the Caco-2 cells and LGG, which is comparable to the actual recorded concentrations in human intestinal tissues, that is, 4.6% (ref. 37; Fig. 2g). The oxygen profiles in the microbial microchamber were characterised by a rapid decrease in the oxygen concentration (from 2.6 to ≤0.8% of dissolved oxygen), following an intermittent spike due to the introduction of small amounts of oxygen into the microbial microchamber during the inoculation process of LGG (Fig. 2g). The established anoxic conditions are analogous to those observed *in vivo* between the mucus layer and the luminal anaerobic zone (∼0.88%; ref. 38) and such oxygen concentrations have been reported to be favourable for the growth of diverse microbiota, including obligate anaerobes^39^. The gradient of oxygen in the HuMiX model was maintained through the continuous perfusion of anoxic media (0.1%) into the microbial microchamber and further shaped by the consumption of oxygen by Caco-2 cells and the facultative anaerobe LGG (Fig. 2g).

Through the consumption of oxygen, anaerobic niches are established in the microbial microchamber, which subsequently allow colonisation of the microbial microchamber by obligate anaerobes^40^. To showcase the ability of HuMiX to sustain culture of an obligate anaerobe, we initiated co-cultures using a simple microbial consortium comprising LGG in combination with *B. caccae* (Supplementary Figs 2b and 3). *B. caccae* was chosen as it represents an obligate anaerobic commensal that belongs to the phylum Bacteroidetes, the other dominant phylum apart from the Firmicutes (LGG) constituting the human GIT microbiome^41^. Both organisms were inoculated in equal starting proportions (optical density (OD) ∼1) and co-cultured with Caco-2 cells for 24 h (Supplementary Fig. 2b). The consortium was sustained via continuous perfusion of anoxic DMEM medium. The consortium structure was determined using 16S rRNA gene amplicon sequencing after 24 h of co-culture, and the relative abundances of *Bacteroides* spp. and *Lactobacillus* spp. were found to be 69 and 31%, respectively (Fig. 2h). These results confirm the ability of the HuMiX model to support the growth of an obligate anaerobic microbial strain. Human cells still exhibited tight junctions (Supplementary Fig. 3a) and both contingents were viable (Supplementary Fig. 3b,c). It follows from these experiments that the inclusion of more complex communities into the HuMiX model is possible but goes beyond the scope of the reported proof-of-concept experiments.

Furthermore, to demonstrate the ability to incorporate other cell types within HuMiX, we cultured non-cancerous colonic cells, i.e., CCD-18Co, in the human microchamber (Supplementary Fig. 4a,b). In addition, to demonstrate that HuMiX can be used in a three-layered set-up for addressing specific research questions, we cultured primary CD4+ T cells in the perfusion microchamber of HuMiX (Supplementary Fig. 4c,d). The primary CD4+ T cells were cultured in the absence (Supplementary Fig. 4c,d) or presence of LGG (Supplementary Fig. 4e,f) over 48 h and did not exhibit any significant differences in terms of cell viability. These experiments highlight the potential of HuMiX to be used for investigating the cellular mechanisms involved in the interplay between GIT bacteria and different human cell types.

In summary, HuMiX exhibits the following essential characteristics: (1) modular microfluidic device architecture consisting of three microchambers engineered to facilitate the proximal co-culture of human and microbial cells; (2) ability to perfuse the device with dedicated culture media to allow the establishment of aerobic conditions for human cell culture and anaerobic conditions for GIT bacteria; (3) real-time monitoring of oxygen concentrations; (4) easy access to the individual cell contingents following specific experimental regimes; and (5) compatibility with end point microscopic assays as well as high-resolution multi-omic analyses.

### HuMiX recapitulates *in vivo* responses

Given the demonstrated ability to establish conditions representative of the human GIT in HuMiX, we conducted further validation experiments to assess the human cellular responses with respect to different co-culture conditions in HuMiX. LGG has been widely used in several human clinical trials aimed at understanding the efficacy of probiotic treatments in humans^32^,^33^. More specifically, gene expression differences have been documented in human intestinal mucosal biopsy samples after the administration of LGG to either healthy subjects^32^ or as a therapeutic supplement for male individuals suffering from esophagitis^33^. Therefore, to validate our *in vitro* co-culture approach, we performed detailed experiments involving the co-culture of Caco-2 cells maintained under aerobic conditions with LGG cultured under anaerobic conditions (Supplementary Fig. 2a) and compared the resulting Caco-2 gene expression data with reference data from clinical studies^32^,^33^. For this, total RNA was first extracted from Caco-2 cells following their co-culture with LGG grown under anaerobic conditions as well as their corresponding LGG-free controls (anoxic medium was perfused through the microbial microchamber, but no bacteria were inoculated, Supplementary Fig. 2a). The RNA was then subjected to DNA microarray-based messenger RNA and microRNA (miRNA) profiling.

**Figure 3:**
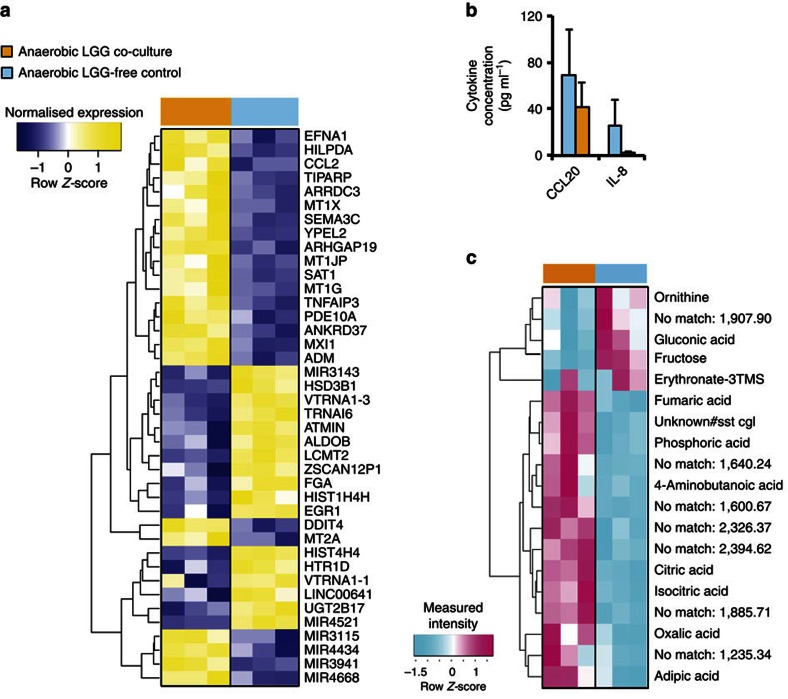
Validation of the HuMiX model by transcriptomic, metabolomics and immunological analyses. (**a**) Heat map highlighting the top 30 differentially expressed genes and miRNAs in Caco-2 cells co-cultured with LGG growing under anaerobic conditions compared with their corresponding LGG-free controls (*n*=3). The threshold parameters used were FC>2 and *P*<0.01, as determined using the empirical Bayes moderated *t*-statistic^65^. Ranking was based on the *π*-values calculated using the log-fold changes and *P* values (BtS). An average linkage hierarchical clustering with the Euclidean distance metric was performed to determine the ordering of the genes. (**b**) Extracellular CCL20/MIP3A and IL-8 cytokine levels before and 24 h after the initiation of co-culture with LGG. Eluate samples were obtained from the perfusion microchamber (*n*=3). (**c**) Heat map of intracellular metabolites from Caco-2 cells co-cultured with LGG growing under anaerobic conditions compared with their corresponding LGG-free controls (*n*=3). The threshold parameter used was *P*<0.1 (StT). An average linkage hierarchical clustering with the Euclidean distance metric was performed to determine the ordering of the metabolites.

Overall, we identified 208 genes that were differentially expressed following co-culture with LGG grown under anaerobic conditions (fold change (FC)>1.5 and equivalently with swapped conditions for decreased expression, *P*<0.01, empirical Bayes moderated *t*-statistic (B*t*S); Fig. 3a; Supplementary Fig. 5a; Supplementary Table 1). Given the lack of detail regarding the identities of the majority of genes found to be differentially expressed *in* *vivo*, we limited our subsequent analyses and discussions to genes that were explicitly highlighted in the *in vivo* clinical studies and that showed statistically significant differences in our study (Table 1). Among the top differentially expressed genes, we validated the gene expression of four genes—*ccl2*, *pi3*, *egr1* and *mt2a—*using quantitative PCR with reverse transcription (RT–qPCR) analyses. The RT–qPCR results showed differential expression patterns analogous to those observed in the microarray data (Supplementary Fig. 5b).

The transcriptomic results exhibit a high level of concordance between the LGG-treated human mucosal *in vivo* transcriptomic data and the differentially expressed gene sets identified through the comparison of HuMiX-based co-cultures with LGG grown under anaerobic conditions compared with the corresponding LGG-free controls^32^,^33^ (Table 1; Supplementary Fig. 5a). The co-culture involving LGG in HuMiX resulted in the up- and downregulation of 127 and 81 genes in the Caco-2 cells, respectively (Supplementary Table 1; FC>1.5 and *P*<0.01, B*t*S). Importantly, the co-culture of Caco-2 with LGG resulted in the differential expression of eight genes (*egr1*, *ccl2*, *slc9a1*, *ubd*, *cxcr4*, *mybl2*, *pim1* and *cyp1a1* (Table 1; Supplementary Fig. 5a: Supplementary Note 2; *P*<0.05, B*t*S)), which had also been found to be differentially expressed in human intestinal biopsy samples after the administration of LGG^32^,^33^. In addition to the genes described above, we also identified four (*elf3*, *cdk9*, *gadd45b* and *pilrb*) genes, previously highlighted as responsive to LGG in human subjects^32^,^33^ (Table 1), but the expression of these genes was found to be disparate when comparing our results to the *in vivo* expression data (Table 1). The highlighted differences in the expression of these four genes are likely due to the reduced complexity of the microenvironment, the human epithelial cells and the microbiota used in our proof-of-concept experiments compared with the *in vivo* situation. In addition, we found a high degree of concordance in responsive pathways (for example, interferon response, calcium signalling and ion homeostasis) in Caco-2 cells following their co-culture with LGG grown under anaerobic conditions when compared to the available *in vivo* mucosal transcriptomic data^32^,^33^ (Supplementary Tables 2 and 3; Supplementary Note 3).

The inoculation of HuMiX with LGG is more similar to the primocolonisation of germ-free animals than its introduction into an already mature GIT microbiome. At present, the only systematic *in vivo* study highlighting the host transcriptomic response to the primocolonisation by LGG was conducted in germ-free piglets^34^. In accordance with the findings from the latter study, our data also highlight a differential expression in eight genes (all *P*<0.03, B*t*S; Table 1; Supplementary Note 4), which also exhibited an altered transcriptional response in mucosal tissues of gnotobiotic piglets 24 h after their inoculation with LGG^34^.

Caco-2 cells are known to secrete distinct cytokines analogous to immune cells when they are challenged with different microbial stimuli. More specifically, the secretion of the pro-inflammatory cytokines interleukin-8 (IL-8) and CCL20 by Caco-2 cells following direct co-culture with microbial strains^42^,^43^ or the application of cell-free microbial supernatants and/or other microbial products is well established^44^. Consequently, they represent a good model for assessing the specific immunological responses to different microorganisms and their products^18^. To test for similar responses in Caco-2 cells when co-cultured in HuMiX, we sampled eluate from the perfusion microchamber (which is in contact with the basal side of the Caco-2 cells) before and 24 h after co-culture with LGG grown under anaerobic conditions, and we screened for immunological markers, including IL-8 and CCL20 (Fig. 3b). No statistically significant increase (paired Student's *t*-test (S*t*T); *P*<0.3) but an apparent slight decrease (Fig. 3b) in the pro-inflammatory cytokines released by the human epithelial cells was observed when they were co-cultured for 24 h with LGG. This observation (Fig. 3b) is in agreement with previous findings, suggesting a subtle anti-inflammatory effect by LGG on human epithelial cells^44^.

In addition to the highlighted cytokine and transcriptional responses of Caco-2 cells, the proximal co-culture of host and microbial cells has the potential to elucidate the complex molecular crosstalk that may induce metabolic changes in the host and microbial cells. Hence, to demonstrate the potential of HuMiX for investigating metabolic interactions between human and microbial cells and for assessing the impact of co-culture on human cellular metabolism, we conducted metabolomic analyses of the intracellular metabolite fractions from the Caco-2 cells when these were co-cultured with LGG growing under anaerobic conditions (Fig. 3c). After 24 h of co-culture, of the 313 metabolites detected, 214 (14 of which were statistically significant (*P*<0.1, S*t*T)) were more and 99 (5 of which were statistically significant (*P*<0.1, S*t*T)) were less abundant in the co-cultured Caco-2 intracellular metabolite fractions when compared with their levels in the corresponding controls (Supplementary Table 4). Sixty-eight per cent of metabolites could not be identified using available metabolite databases. Five unknown metabolites that were present in control samples were not detected in the Caco-2 metabolite fractions following co-culture. The intracellular levels of certain tricarboxylic acid cycle intermediates increased. In particular, the intracellular concentrations of fumaric acid (FC>3, *P*<0.05, S*t*T), citric acid (FC>6, *P*<0.05, S*t*T) and isocitric acid (FC>6, *P*<0.07, S*t*T; Fig. 3c) increased significantly (Supplementary Table 4). Interestingly, the increase in tricarboxylic acid cycle intermediates agrees with the previous observations of similar increases in the blood serum of germ-free mice upon their conventionalisation^45^.[Fig f4]

Furthermore, the apparent decrease in intracellular concentrations of urea (FC>2, *P*<0.2, S*t*T; Supplementary Table 4) after inoculation with LGG was analogous to the earlier reports describing the induced metabolic changes following the conventionalisation of germ-free mice^45^. Our transcriptomic data further revealed that the *cps1* gene was downregulated in Caco-2 cells following their co-culture with LGG grown under anaerobic conditions (Fig. 5a; Supplementary Fig. 8; FC>1.4, *P*<0.05, B*t*S). The CPS1 protein is the first and rate-limiting step of the urea cycle that converts ammonia to carbamoyl phosphate. CPS1 has previously been found to be expressed in intestinal epithelial cells^46^, and our results suggest that microbiome-mediated modulation of ureagenesis may occur in the GIT.

Analogous to the experiments involving Caco-2 cells, we also conducted a metabolomic investigation of the intracellular LGG metabolite fractions after co-culture with Caco-2 cells and compared the results with those derived from mono-cultured LGG to further investigate crosstalk between the Caco-2 cells and LGG. Interestingly, 170 intracellular metabolites (representing 47% of all metabolites detected) were reduced or even undetectable after the co-culture with Caco-2 cells (*P*<0.05, S*t*T; Supplementary Fig. 6; Supplementary Table 5). Furthermore, fumaric acid was one of the metabolites under the detection limit after co-culture with Caco-2 cells (*P*<0.05; Supplementary Table 5). The concomitant increase in the intracellular fumaric acid concentration in the Caco-2 cells (Fig. 3c) suggests possible cross-feeding of this metabolite between the Caco-2 and LGG cells. Furthermore, this suggests that the catalytic activity of the enzyme succinate dehydrogenase might be differentially regulated in bacteria compared with human cells following their co-culture. Most of the metabolites detected (77%) did not result in a direct match in the available databases (Supplementary Fig. 6a). Interestingly, 51 of those metabolites were only discovered in the mono-cultured LGG but were not discovered in the intracellular LGG metabolite fraction after co-culture with Caco-2 cells (*P*<0.05, S*t*T; Supplementary Table 5). Intriguingly, three unknown (no match) metabolites were detectable in the intracellular LGG pool only after the co-culture with Caco-2 cells (*P*<0.05, S*t*T; Supplementary Table 5). These results suggest significant shifts in LGG metabolism owing to extensive cross-feeding with the human epithelial cells. Our results further confirm that despite the presence of a partitioning nanoporous membrane between the epithelial cells and LGG in the HuMiX model, there exists an efficient crosstalk between the human and microbial cells, as demonstrated by the specific physiological responses in both human epithelial and bacterial cells following their co-culture in HuMiX.

Taking into account in particular the concordance between the transcriptional responses of the epithelial cells co-cultured with LGG in HuMiX and *in vivo* expression data obtained from human and piglet studies, the presented results validate the HuMiX model and support the notion that this model may be regarded as an alternative to animal models for first-pass experiments aimed at elucidating host–microbial molecular interactions and their effects on the host.

### HuMiX-based co-cultures with a bacterial consortium

**Figure 4 f4:**
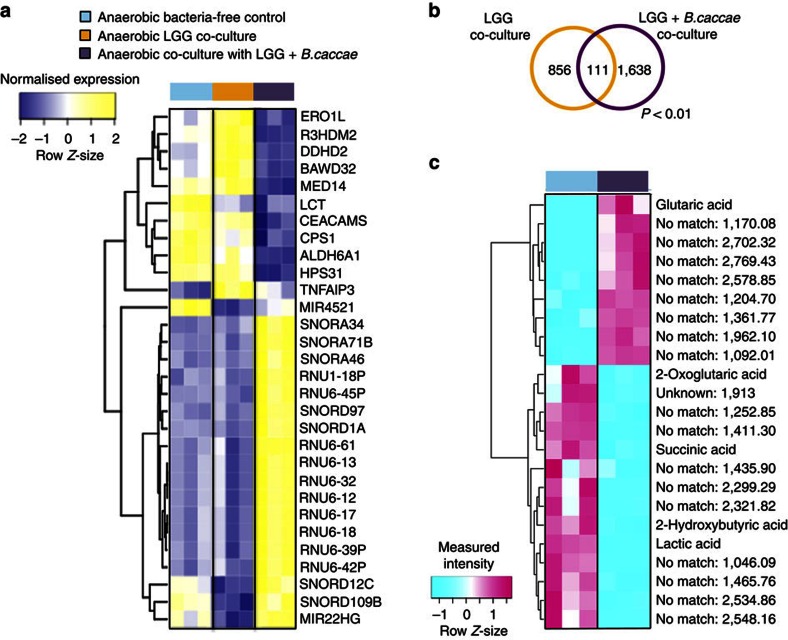
Transcriptional and metabolic changes induced in human cells following their co-culture with LGG and *B. caccae*. (**a**) Heat map highlighting the top 30 differentially expressed genes and miRNAs in Caco-2 cells co-cultured with either LGG alone or LGG and *B. caccae* growing under anaerobic conditions compared with their bacteria-free controls (*n*=3). The threshold parameters used were FC>2 and *P*<0.01, as determined using the empirical Bayes moderated *t*-statistic^65^. Ranking was based on the *π*-values calculated using the log-fold changes and *P* values (BtS). An average linkage hierarchical clustering with the Euclidean distance metric was performed to determine the ordering of the genes. (**b**) Venn diagram comparing the gene expression patterns obtained when Caco-2 cells were co-cultured with LGG or with a consortium of LGG and *B. caccae* growing under anaerobic conditions. The threshold parameters used were FC>1.5 and *P*<0.01 (B*t*S). (**c**) Heat map of intracellular metabolites from Caco-2 cells co-cultured with LGG and *B. caccae* growing under anaerobic conditions in comparison with monocultures of Caco-2 cells for which anaerobic medium was perfused through the microbial microchamber. The threshold parameter used was *P*<0.1 (S*t*T). An average linkage hierarchical clustering with the Euclidean distance metric was performed to determine the ordering of the metabolites.

To evaluate the effect of a bacterial consortium on Caco-2 cells, *B. caccae* and LGG were both placed in co-culture with Caco-2 cells, whereby the bacterial consortium was maintained under anaerobic conditions (Supplementary Fig. 2b). The addition of *B. caccae* lead to a significant change in the transcriptional response of the Caco-2 cells in comparison with the response when Caco-2 cells were co-cultured solely with only LGG (Fig. 4a).

Interestingly, following the inclusion of *B.* caccae, only 6 genes (*slc9a1*, *elf3*, *mybl2*, *gadd45b*, *igfbp2* and *gsta1*) out of the previously highlighted 19 genes (Table 1) which showed differential gene expression under an LGG anaerobic co-culture regime as well as in the *in vivo* human clinical studies^32^,^33^,^34^, were identified to be differentially expressed in the Caco-2 cells. However, three additional Caco-2 genes (*ndrg3*, *hmgcs2* and *cyr61*, all FC>1.5, *P*<0.08; Supplementary Note 5) earlier highlighted in human clinical trials to be differentially expressed after LGG administration were found to be differentially expressed only after co-culturing with LGG and *B. caccae*, which suggests that consortium-driven synergistic mechanisms are likely at play^32^,^33^ (Supplementary Table 6). Overall, we found that 1,638 human genes exhibited differential expression specifically when Caco-2 cells were co-cultured with LGG and *B. caccae* compared with 856 genes that were differentially expressed by Caco-2 cells when solely co-cultured with LGG (Fig. 4b; *P*<0.01, B*t*S). One hundred and eleven genes showed a similar expression pattern under both co-culture conditions (Fig. 4b; *P*<0.01, B*t*S).

Furthermore, we analysed the intracellular metabolite fractions of the Caco-2 cells to determine the induced effects as a result of the co-culture regimes involving LGG and *B. caccae* (Fig. 4c). Analogous to the transcriptional response, the intracellular metabolite fractions of the Caco-2 cells were significantly altered in response to the consortium co-culture as compared with the cells co-cultured solely with LGG (Fig. 3c; Supplementary Table 7). Our results demonstrate that the HuMiX model is capable of capturing transcriptional and metabolic responses of the human epithelial cells in response to changes in the composition of the co-cultured microorganisms.

### Anaerobic or aerobic bacterial co-affects human transcriptome

**Figure 5:**
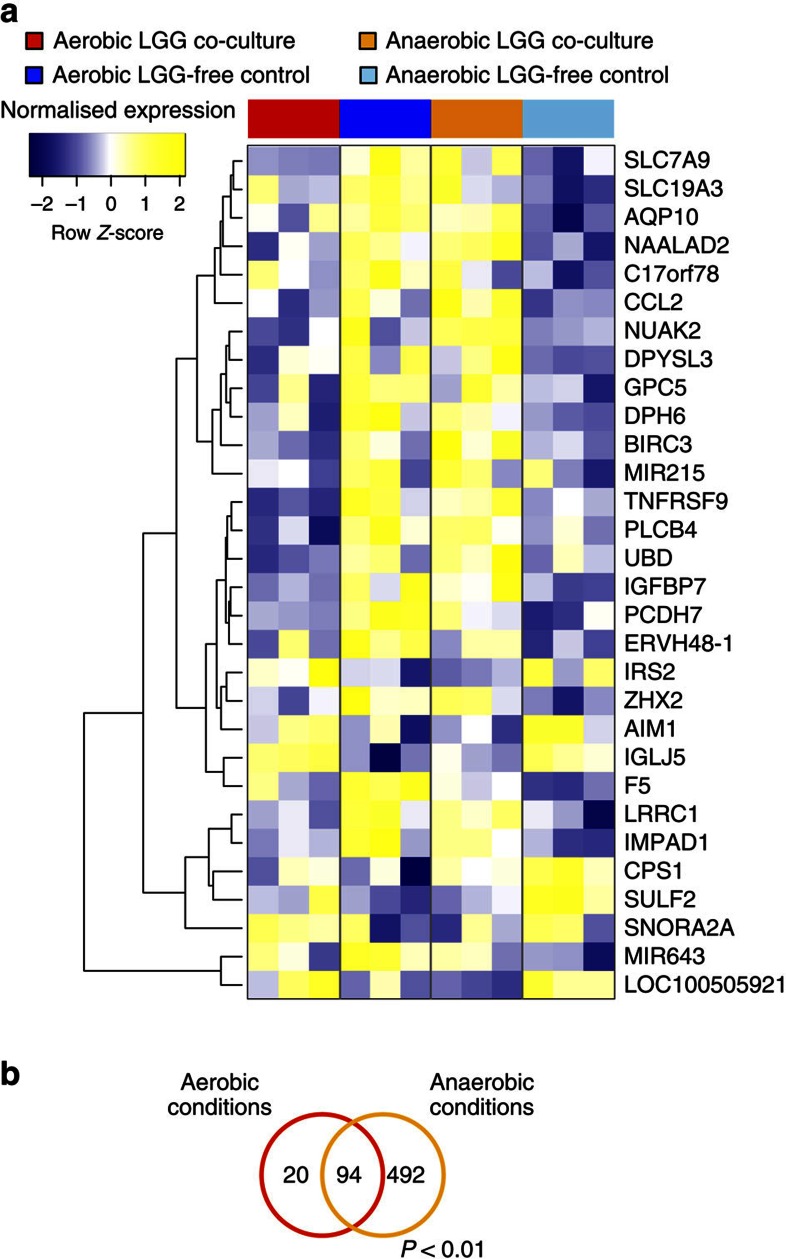
Anaerobic or aerobic bacterial culture differentially affects human transcriptional responses. (**a**) Heat map representing the top 30 genes and miRNAs that exhibit opposite expression patterns in Caco-2 cells when co-cultured with LGG growing under either anaerobic or aerobic conditions compared with their respective LGG-free controls. The ranking was based on the *π*-values calculated using log-fold changes and *P* values (BtS). An average linkage hierarchical clustering with the Euclidean distance metric was performed to determine the ordering of the genes. (**b**) Venn diagram comparing the numbers of genes differentially expressed by Caco-2 cells following their co-culture with LGG growing under anaerobic or aerobic conditions. The threshold parameters used were FC>1.5 and *P*<0.01 (BtS).

Since HuMiX offers the possibility to co-culture human cells with bacteria growing under anaerobic conditions (that is, mimicking the conditions in the GIT), we explored the potential benefits of such co-cultures in contrast to traditional co-culture approaches that maintain bacteria under aerobic conditions^10^, which are likely to induce non-representative changes in bacterial metabolism^31^ and consequential effects in human cells. For this, we compared the gene expression patterns of Caco-2 cells following 24 h of co-culture with LGG grown under anaerobic conditions (≤0.8% dissolved oxygen) or aerobic conditions (21%; Fig. 5a,b; Supplementary Fig. 2c, Supplementary Table 8).

The generic Caco-2 response to co-culture with LGG was first determined by focusing on the genes that exhibited similar expression patterns under both LGG culture conditions compared with their respective LGG-free controls (Supplementary Fig. 2a,c; Supplementary Fig. 7). Ninety-four genes exhibited differential expression under either of the two co-culture conditions (Fig. 5b; Supplementary Fig. 7; *P*<0.01, B*t*S). Conversely, genes that were differentially expressed under either condition were determined to be specific to one of the two conditions, that is, LGG grown under anaerobic conditions or aerobic conditions. Overall, we identified 492 human genes that exhibited differential expression specifically when Caco-2 cells were co-cultured with LGG growing under anaerobic conditions, whereas 20 genes were specifically expressed by Caco-2 cells co-cultured with aerobically growing LGG (Fig. 5a,b; Supplementary Fig. 8; *P*<0.01, B*t*S).

Among the top differentially expressed genes in Caco-2 cells when co-cultured with LGG grown under anaerobic conditions, we identified four human genes (*ccl2* (*P*<0.001), *egr1* (*P*<0.005), *ubd* (*P*<0.05) and *slc9a1* (*P*<0.05)) that exhibited expression patterns identical to those observed in mucosal biopsy samples obtained from healthy human subjects following the administration of the probiotic LGG (Fig. 5a; Supplementary Fig. 8; Table 1; Supplementary Table 8, all FC>1.5, B*t*S)^32^,^33^. Intriguingly, when the Caco-2 cells were co-cultured with LGG growing under aerobic conditions instead, these genes were either up- or downregulated in one co-culture versus control pair situation, and exhibited the opposite trend in the other scenario (Fig. 5a; Supplementary Fig. 8). Among the genes that presented such opposing expression patterns, we identified a number of genes that play important roles in the regulation of inflammatory responses, maintenance and regulation of epithelial barrier function, mediation of host–microbe interactions, and regulation of cancer-related pathways (Supplementary Fig. 8; Supplementary Note 6). In addition, we found four genes (*cxcr4*, *pim1*, *cyp1a1* and *mybl2*, *P*<0.05, B*t*S), which had previously been identified in human clinical trials to be differentially expressed in the presence of LGG^32^,^33^, to exhibit a more generic response to co-culture with LGG, that is, similar expression in Caco-2 cells when co-cultured with LGG under either condition (Supplementary Fig. 7; Table 1). The differential expression of cancer-related genes in cancer-derived Caco-2 cells following their co-culture with LGG is interesting and further investigations are required to determine whether this is a generic response by human epithelial cells or whether this is limited to cancer-derived cells. In all of the presented results, as the gene expression profiles of the co-cultured cells have been compared with mono-cultured Caco-2 cells, the effects observed are attributable to the influence of the co-cultured bacteria on the Caco-2 cells.

To further define the effects of LGG on Caco-2 cells when LGG was grown in two distinct oxygen conditions, a pathway enrichment analysis was conducted this time using only the Caco-2 genes that exhibited contrasting gene expression patterns (the threshold parameters used were FC>1.5 and *P*<0.05, B*t*S; Supplementary Table 9). The pathways that exhibited differential expression based on the contrasting gene expression patterns were linked to gut motility, immune response, cell cycle, cell adhesion, apoptosis, cytoskeleton remodelling, lipid metabolism regulation, signal transduction and developmental signalling pathways (Supplementary Table 9; Supplementary Note 6). An additional data-driven pathway analysis using the gene ontology database revealed that the top enriched pathways exhibiting contrasting gene expression patterns under anaerobic or aerobic conditions were related to metabolism (more specifically, lipid, protein and carbohydrate metabolism), cellular homeostasis, amino-acid transport and particularly adaptive immune responses (Supplementary Table 10; Supplementary Note 6).

Given the pivotal role of anaerobic conditions in the GIT for the maintenance of the GIT microbiota composition^47^, host–microbe mutualistic interactions^48^ and possibly dysbiosis^49^, the obtained results represent an important validation of the HuMiX approach for representative studies of host–microbe interactions. On the basis of these results, the existing models, which typically involve the co-culturing of bacteria and human cells under aerobic conditions, induce a partial and partly non-representative transcriptional response in Caco-2 cells and this highlights the importance of maintaining anaerobic culture conditions when co-culturing GIT bacteria with human cells. The ability to maintain bacteria under anaerobic conditions therefore represents an essential functionality of the HuMiX model.

### Discovery-driven investigations of host-microbe interactions

Although the primary purpose of our experimental work was to validate the HuMiX model in relation to already existing knowledge primarily from *in vivo* studies, our multi-omic data also potentially allow novel insights in the context of host–microbe molecular interactions. More specifically, the opportunity to comprehensively mimic and probe the individual cell contingents using high-resolution molecular analyses provides an unprecedented opportunity to study the effects of live bacterial cells growing under representative environmental conditions in close proximity to human cells. Here we describe interesting observations obtained following the co-culture of Caco-2 cells with LGG or with the LGG and *B. caccae* consortium when these were maintained under anaerobic conditions.

**Figure 6:**
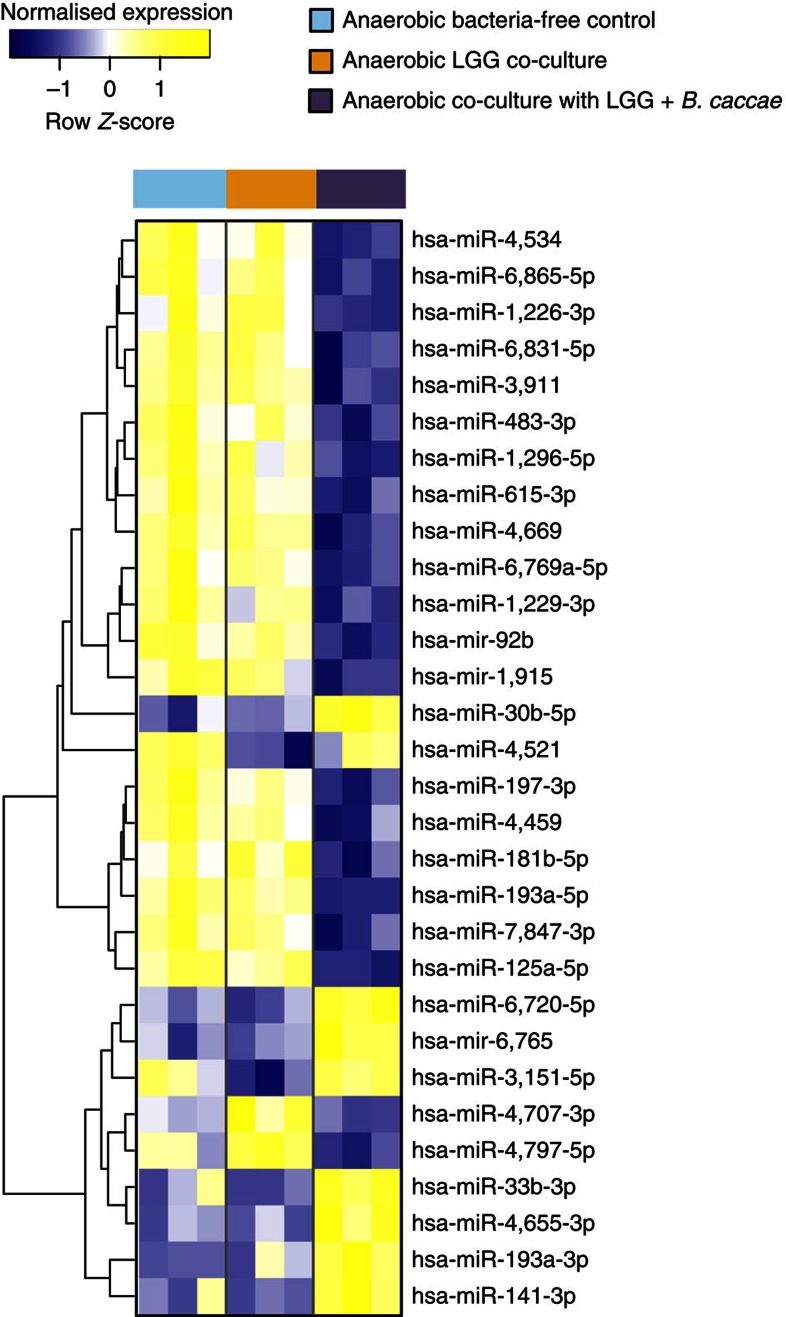
Co-culture regime-specific miRNA expression. Heat map of the top 30 statistically significant differentially expressed genes in Caco-2 cells when comparing their expression after co-culture with LGG, LGG and B. caccae and their corresponding bacteria-free controls. The threshold parameters used were FC>1.5 and *P*<0.05 (B*t*S). An average linkage hierarchical clustering with the Euclidean distance metric was performed to determine the ordering of the genes.

*Co-cultured microorganisms alter expression of miRNAs linked to colorectal cancer in Caco-2 cells*. Following co-culture with LGG or LGG with *B. caccae* grown under anaerobic conditions, miRNA profiling highlighted differential regulation of a vast number of miRNAs (mir483-3p, mir1229-3p, mir92b, mir1915, mir30b-5p, mir4521, mir193a-5p, mir125a-5p and mir141-3p) linked to colorectal cancer (Fig. 6). Notably, many of these have been recently added to the panels of biomarkers for diagnosis and prognosis of gastrointestinal cancers^50^,^51^,^52^,^53^,^54^. Many of these miRNAs were only differentially expressed in the presence of LGG, while the expression of others was altered by the presence of *B. caccae* in the consortium. Despite the fact that Caco-2 cells are cancer- derived, our results demonstrate that the presence of different bacteria leads to a differential regulation of the expression of these cancer-related miRNAs. These results underpin the notion that HuMiX may prove valuable as a screening tool for identifying and validating biomarker signatures (Supplementary Note 7) and for testing microbiome-based therapeutic interventions, for example, in the context of colorectal cancer.

*LGG induces the accumulation of GABA in epithelial cells*. The intracellular accumulation of GABA (4-aminobutanoic acid) in Caco-2 cells following co-culture with LGG grown under anaerobic conditions (Fig. 3c, FC=2.18, *P*<0.06, S*t*T; Supplementary Note 8) is similar to previous observations in pulmonary epithelial cells^55^, in which GABA was found to subsequently contribute to the relaxation of smooth muscle tone^56^. *ccl2* (FC>1.5, *P*<0.001, B*t*S), which was ranked among the top 10 differentially expressed genes in our co-culture experiments (Table 1) as well as *in vivo* transcriptomic data following LGG administration^32^,^33^, has been shown to interact with GABA and to contribute to the regulation of the GABAergic response in neurons^57^. In addition, the expression of the *gad1* gene was slightly upregulated following the co-culture of Caco-2 cells with LGG grown under anaerobic conditions (Supplementary Fig. 7; FC ∼1.2, *P*<0.05, B*t*S). *gad1* encodes one of several forms of glutamic acid decarboxylase that catalyses the conversion of L-glutamic acid into GABA and may thus be involved in the marked increase in the intracellular GABA concentrations in Caco-2 cells. The exact mechanism of GABA accumulation in the Caco-2 cells in our experiments and its potential local and systemic effects *in vivo* are important research questions for future HuMiX-based investigations but go beyond the scope of the present study. Given its modularity and flexibility for inclusion of additional cell types, the HuMiX model may allow detailed investigations of the molecular mechanisms governing the gut–brain axis in the future.

### Conclusion

Our detailed experimental results demonstrate that HuMiX is a representative model of the gastrointestinal human–microbe interface, as individual transcriptional responses from human epithelial cells co-cultured with LGG inside HuMiX are in agreement with *in vivo* data. HuMiX also allows discovery-based studies particularly in relation to proving causal relationships between gastrointestinal microbiota and human diseases. Although HuMiX was developed with a focus on host–microbe interactions, it may find applications in a number of other domains including drug screening, drug discovery, drug delivery as well as in pharmacokinetics and nutritional studie. The ability to co-culture human and microbial cells in a controlled manner and perform systematic investigations of such co-cultures opens up numerous avenues for basic and applied research in the context of the human microbiome in the future.

## Methods

### Fabrication and assembly of the HuMiX device

The HuMiX device is comprised of two polycarbonate (PC) enclosures, which sandwich silicone rubber gaskets, which are themselves attached to semi-permeable PC membranes (Fig. 1b; Supplementary Fig. 1a,b). PC was chosen as the material for the enclosures because it is easy to machine, can be sterilised by autoclaving and is gas impermeable, which is essential for controlling the oxygen concentrations within the device. The enclosures were fabricated by computer numerically controlled milling of 6.2-mm-thick PC sheets (Professional Plastics). Towards their inner side, the enclosures have machined pockets for inclusion of optical sensor spots (optodes) for oxygen sensing (Fig. 1b; Supplementary Fig. 1c) and perforations, which allow the bolting of the device together (Fig. 1b; Supplementary Fig. 1a). To delineate the individual spiral-shaped microchambers (Fig. 1c; Supplementary Fig. 1b), 0.79-mm-thick super-soft silicone sheets were laser-cut to form gaskets. Medical-grade double-sided adhesive tapes (Adhesives Research Inc.) were attached to the gaskets and to these were then affixed the semi-permeable PC membranes. The perfusion microchambers were assembled by attaching a 1-μm pore size PC membrane (Whatman, GE Healthcare) to the gasket, whereas a 50-nm pore size PC membrane (Whatman, GE Healthcare) was attached to the gasket delineating the human microchamber from the microbial microchamber (Fig. 1b). For assembly, the gaskets were precisely aligned and sandwiched between the top and bottom enclosures. The HuMiX devices were then bolted together using M2 screws and nuts (McMaster-Carr). A special version of the HuMiX device with additional side pockets was also fabricated to allow insertion of the industry standard chopstick style STX2 electrode (WPI Inc.) to characterise the growth of epithelial cells inside the device (Supplementary Fig. 1d). The gaskets with the attached membranes are disposable and cannot be reused. The PC enclosures can be reused after proper cleaning and sterilisation.

### Sterilisation of the HuMiX device

A robust sterilisation procedure was developed and optimised to completely avoid both fungal and bacterial contamination in any of the microchambers. All the parts (enclosures, gaskets, screws, nuts, syringe needles, peristaltic pump tubings, media bottles and so on) and materials (handling equipment, mucin and so on) used for the assembly and testing of the HuMiX device were autoclaved at 110 °C for 60 min. Before assembly of the tubings as well as the HuMiX device, the PC enclosures were thoroughly cleaned with 70% v/v ethanol followed by air drying under a laminar flow hood. Dead-ended (knotted) silicone tubings were attached to the inlet and outlet connectors (Supplementary Fig. 1c) during autoclaving to keep the connector vias sterile during handling and assembly. In addition, the PC enclosures were also exposed to ultraviolet for 10–20 min under a laminar flow bench before assembly.

### Coating of the membranes and permeability assays

After autoclaving, the 1-μm pore size PC membranes, affixed to the gaskets, were coated using 4 ml of 50 μl ml^−1^ rat tail collagen solution (Life Technologies; prepared in 0.02 M acetic acid) for 3 h. The collagen stock solution was sterilised by layering the solution over 10% (v/v) chloroform overnight at 4 °C, after which the top collagen layer was aseptically removed^58^. The 50-nm pore size PC membranes were covered with autoclaved 0.025 mg ml^−1^ porcine gastric mucin (Sigma-Aldrich) solution for 1 h. Excess solution was removed after the coating procedure and the membranes were air dried for 30 min under sterile conditions. The thickness of the mucin layer was characterised using confocal laser scanning microscopy by embedding 4 kDa fluorescein isothiocyanate-conjugated dextran (Sigma-Aldrich) in the mucin solution^23^. For the characterisation of the permeability of the HuMiX set-up, a HuMiX device was assembled with coated micro- and nanoporous membranes. A water solution containing 4 kDa fluorescein isothiocyanate-conjugated dextran was perfused into the microbial microchamber and samples were collected from perfusion microchamber at regular intervals to determine the permeability of the HuMiX device as described in Marzorati *et al*.^23^

### Maintenance of cells and microbial cultures

The human epithelial colorectal cell line Caco-2 (DSMZ: ACC169) and non-cancerous colonic cell line CCD-18Co (ATCC CRL-1459) were maintained at 37 °C in a 5% CO_2_ incubator in DMEM medium (Sigma-Aldrich) supplemented with 20% fetal bovine serum (FBS; Life Technologies) and 1% penicillin–streptomycin (Sigma-Aldrich) until use and passaged at 80–100% confluence. Only cells between passages 7 and 28 were used for this study. Primary CD4+T cells were purified from healthy blood donors (Red Cross Luxembourg) via magnetic labelling and subsequent separation on an LS column (Miltenyi Biotech). Before inoculation of the immune cells into HuMiX, they had been activated with IL-2 (10 ng ml^−1^), CD3 antibodies (5 μg ml^−1^) and CD28 antibodies (5 μg ml^−1^) for 24 h.

LGG (ATCC: 53103) was cultured in aerobic as well as anaerobic DMEM medium supplemented with 20% FBS without antibiotics in a shaking incubator at 37 °C and 200 r.p.m. *B. caccae* (DSMZ: 19024) was cultured in anoxic DMEM medium supplemented with 20% FBS and haeme or HBIB medium. Before inoculation in HuMiX device, the microbial suspensions were pelleted and washed with 0.9% NaCl before suspending them in 5 ml fresh anoxic DMEM medium. The microbial suspensions were mixed in equal proportions for consortium co-cultures with the Caco-2 cells in HuMiX. Fresh microbial cultures were started from glycerol stocks and cultured for 6–24 h before inoculation into the HuMiX device.

### HuMiX co-cultures using Caco-2 cells

Before inoculation of the Caco-2 cells into the HuMiX device, the tubings (peristaltic pump tubes as well as the tubes to be later connected to the HuMiX devices) were connected to 0.5-l serum bottles (Glasgerätebau Ochs) containing the culture media (Fig. 1d). The HuMiX device was then integrated into the tubing set-up by attaching the elastomeric tubing to the connectors of the HuMiX device. To prime the HuMiX device, DMEM medium supplemented with 20% of FBS was perfused at 0.65 ml min^−1^ through all the channels of the HuMiX device using a programmable 205CA peristaltic pump (Watson Marlow). After the priming of the HuMiX set-up to remove air bubbles, the entire set-up was moved into to a 5% CO_2_ incubator set to 37 °C. At this moment in time, the flow rate was reduced to 5 μl min^−1^ for at least 1−2 h to equilibrate the entire HuMiX device and to coat the chamber with medium constituents to improve subsequent cell adhesion. Following equilibration, the HuMiX device and tubing set-up was moved back to the laminar flow bench and inoculated with human epithelial cells (Caco-2) by injecting a 1-ml cell suspension (6 × 10^5^ cells per ml) into the human microchamber using a sterile syringe (Becton Dickinson) via a Discofix three-way adaptor (B. Braun). Subsequently, the HuMiX device was flipped and incubated at 37 °C without flow for 2 h to allow attachment of cells to the collagen-coated microporous membrane. After 2 h, the Caco-2 cells were perfused at their basal side via the perfusion microchamber with DMEM medium supplemented with 20% FBS at a flow rate of 25 μl min^−1^. Perfusion through the microbial microchamber was simultaneously initiated. If the Caco-2 cells were later to be co-cultured with LGG or LGG and *B. caccae* grown under anaerobic conditions, perfusion with anoxic DMEM medium was carried out at the same flow rate of 25 μl min^−1^. Anoxic DMEM medium was obtained by constantly bubbling the medium with dinitrogen gas through an aeration needle placed inside the media bottle (B. Braun). For each microbial culture condition (LGG growing either under anaerobic or aerobic conditions, Supplementary Fig. 2a,c), bacterial cells were introduced into the microbial microchamber on day 7 following initiation of the Caco-2 cell culture. At day 7, the Caco-2 cells were found to have formed tight junctions (Fig. 2b) and the cell number (∼1 × 10^6^) had reached a value allowing downstream molecular analyses. The LGG or LGG and *B. caccae* cell suspensions were then introduced into the microbial microchamber using a Discofix two-way adaptor in a 1-ml suspension (OD ∼1) and later perfused with anoxic or oxic DMEM medium at 25 μl min^−1^. Following 24 h of co-culture, the devices were disassembled and the human and microbial gaskets were separated. For further detailed analyses, the gaskets were divided into three parts, whereby half of the cell-covered membranes were used for extraction of the intracellular metabolites, DNA, RNA and proteins using a comprehensive biomolecular extraction protocol^36^. The other two quarters were used for live–dead staining (Calbiochem Millipore staining kit and L7007 BacLight microbial viability kit Molecular Probes) as well as immunostaining for subsequent fluorescence microscopy. As controls, Caco-2 and LGG were cultured individually on separate devices. The co-culture protocol is graphically depicted in Fig. 1e.

### HuMiX co-cultures involving CCD-18Co cells

After the assembly and priming of the HuMiX device, the CCD-18Co cells were inoculated in the human microchamber and supplied with fresh DMEM medium (supplemented with 20% FBS and 1% penicillin–streptomycin ) via simultaneous perfusion in the microbial and the perfusion chamber. After 7 days of culture, the device was disassembled, CCD-18Co cells attached to the membrane were stained with Alexa Flour 568 Phalloidin and 4,6-diamidino-2-phenylindole, and the cells on the membrane were subsequently visualised using fluorescence microscopy for evaluating the growth and viability (Supplementary Fig. 4a,b).

### HuMiX co-cultures involving primary immune cells and LGG

To initiate the co-culture, 5 × 10^6^ primary human CD4+ T cells were inoculated in the perfusion microchamber (Supplementary Fig. 4c). 24 h later, LGG (OD ∼1) was inoculated in the microbial microchamber (Supplementary Fig. 4e). Both cell types were then co-cultured for another 24 h. The perfusion and the microbial microchamber were perfused with either oxic or anoxic DMEM medium for 48 h, respectively. The viability of the CD4+ T cells either cultured solely (Supplementary Fig. 4c,d) or cultured in the presence of LGG (Supplementary Fig. 4e,f) was determined using the LIVE/DEAD fixable near IR cell kit (Molecular Probes) and flow cytometry.

### Oxygen sensing

The integrated optical sensors (optodes) allowed for continuous, non-invasive and non-cytotoxic detection of oxygen levels in the HuMiX device (Fig. 1a; Supplementary Fig. 1c). The 5-mm diameter pst3 optodes (sensitivity of up to 0.03% of O_2_; PreSens) were bonded into the 1.2-mm deep machined pockets by application of 4-μl silicone adhesive (PreSens) and cured overnight. Optodes were affixed to both PC enclosures 20 mm adjacent to the inlets and outlets of the perfusion microchamber and microbial microchamber, respectively (Fig. 1a; Supplementary Fig. 1a). On the outer surface, the enclosures housed 4.5-mm deep pockets for attachment of the polymer optical fibres used for transmitting the oxygen measurement signals to the recording device (Supplementary Fig. 1c). Importantly, the distance between the optode and sensing head of the optical fibre was 0.5 mm as per the manufacturer's recommendation (PreSens). The oxygen concentration was measured every 15 min using an OXY-4 trace oxygen transmitter/recorder (PreSens) and logged using the oxy4v2_41FB software (PreSens) using a connected personal computer.

### Epithelial barrier measurements

As detailed above, a dedicated HuMiX-TEER device was fabricated to allow measurement of TEER using a STX2 electrode connected to a Millicell ERS-2 Epithelial Volt-Ohm Meter during cell culture trials in HuMiX (Millipore; Supplementary Fig. 1d). For this, double-electrode pairs of the chopstick style STX2 electrodes (Millipore) were introduced into the perfusion and microbial microchambers, respectively. Before inoculation of the human cells, TEER values were recorded to determine the background resistance of the HuMiX device after all the channels were filled with DMEM medium. The background TEER was subtracted from the readings subsequently to infer the growth curve of Caco-2 cells by following the increase in resistance. As the insertion of chopstick electrodes through the side ports can lead to contamination of the experiment, the TEER measurements were conducted as an end point assay and related to epithelial cell barrier formation ascertained following immunostaining and fluorescence microscopic analyses. The HuMiX-TEER device was in particular used to determine the optimal point in time when the cells had differentiated for the subsequent inoculation of bacteria into the device. As a reference, Caco-2 cells were also inoculated in the standard Transwell systems and supplied with antibiotic-free DMEM medium analogous to the cultivation conditions in HuMiX. Here again, the background TEER was also measured in Transwell inserts before inoculation with Caco-2 cells and later subtracted from the actual TEER readings.

### Cytokine profiling of eluate samples

To assess possible immunological responses of the epithelial cells to the co-cultured bacteria, 150−200 μl of eluate samples were collected from the perfusion microchamber, flash-frozen and preserved at −80 °C until further analysis. A measure of 50 μl of the eluates collected from the perfusion microchamber before and after inoculation with LGG was analysed for eight different cytokines using a Human Premixed Multi-Analyte Kit (R&D Systems, Europe; UK) in combination with the multiplex reader MAGPIX (Luminex) according to the manufacturers' instructions. The kit allowed screening for the following human cytokines of interest: CXCL8/IL-8, CCL20/MIP-3 alpha, GM-CSF, IL-1 beta, IL-6, IL-10, IL-12p70 and TNF-alpha. Only CCL20 and IL-8 were detectable in the samples.

### Sampling of cellular material post culture

Twenty-four hours after the initiation of co-culture of Caco-2 cells with LGG or LGG and B. *caccae*, perfusion of the device was stopped and the device was disassembled using a manual screwdriver. The gasket–membrane assemblies bearing the cells (Caco-2 or LGG or LGG and B. *caccae*) were then cut into two halves: one half was used for comprehensive biomolecular extractions and the other half for staining and microscopic inspection.

### Live–dead analyses

Live–dead staining was performed for determining Caco-2 and bacterial cell viability. The Calbiochem kit (Millipore) was used on the microporous membranes containing the Caco-2 cells, whereas the BacLight microbial viability kit (Molecular Probes) was applied to the nanoporous membranes containing LGG or LGG and *B. caccae*. Before staining the membrane-bound Caco-2 cells, the membranes were separated from the gaskets using tweezers. A measure of 200 μl of staining buffer from the Calbiochem kit was then applied to one quarter of the membranes and incubated at 37 °C for 15 min. For microbial staining and visualisation, one quarter of the membranes was stained using the L7007 BacLight microbial viability kit (Molecular Probes). A measure of 200 μl of staining solution was prepared as per the manufacturer's recommendation and applied to the gaskets followed by incubation at 37 °C for 15 min. After removal of the staining buffer, the unbound stains were removed by washing of the membrane-bound human and microbial cells with 1 × PBS solution at pH 7.2. The stained cells on the membranes were then fixed in a 4% paraformaldehyde (PFA)/1 × PBS solution for 10 min at room temperature in the dark. After an additional wash in 1 × PBS, the fixed membranes were mounted on microscope slides using ProLong Gold anti-fading mounting medium (Life Technologies, Europe). The microscope slides were then allowed to dry overnight at room temperature in the dark and observed at appropriate excitation wavelengths using a Zeiss 710 Meta confocal laser scanning microscope to evaluate the morphology and viability of the cells.

### Fluorescence microscopic analysis of co-cultured cell contingents

Apart from live–dead staining, the membrane-bound human and microbial cells were also stained with specific dyes and analysed by fluorescence microscopy. For the detection of occludin, a quarter of the microporous membranes covered by the Caco-2 cells were fixed in a 4% (v/v) PFA/PBS solution for 10 min at room temperature, washed with 1 × PBS and then blocked for 15 min with 5% bovine serum albumin (BSA; Sigma-Aldrich) in 1 × PBS. The cells were then stained with anti-Occludin mAb Mouse Alexa Fluor 488 conjugate (Life Technologies, Europe) diluted 1/200 in 1% (v/v) BSA/PBS solution for 30 min at room temperature in the dark. Subsequently, the membrane-bound cells were washed three times with 1 × PBS to remove unbound fluorescently labelled antibodies. For staining the Caco-2 cell nuclei, 200 μl of Hoechst stain (Life Technologies, Europe) diluted 1:1,000 in 1% (v/v) BSA/PBS solution was applied to the cells and incubated for 2 min at room temperature in the dark. After three consecutive washes in 1 × PBS, excess solution was removed and the membranes were mounted on a microscope slide using ProLong Antifade Reagent (Life Technologies, Europe). The microscope slides were then allowed to dry overnight at room temperature in the dark and observed at appropriate excitation wavelengths using a Zeiss 710 Meta confocal laser scanning microscope to visualise cell nuclei and tight junctions.

The membrane-bound LGG or LGG and B. *caccae* cells were fixed by application of 4% (v/v) PFA/PBS solution at room temperature for 10 min. After three washes in 1 × PBS to remove excess fixing solution, the membranes were incubated in the dark for 5 min at room temperature in 1:1,000 Hoechst/PBS solution. After a wash in a 1 × PBS to remove excess staining solution, the membranes were mounted on microscope slides using the ProLong Antifade Reagent (Life Technologies, Europe) and the cells were visualised using a Zeiss 710 Meta confocal microscope.

### Microscopic image processing

Confocal image *z*-stacks were acquired using the Zeiss Zen Black software suite. *Z*-stacks were processed using Imaris 8 (Bitplane AG) coupled with the AutoQuant suite (x 3.0.0, Media Cybernetics) for background correction.

### Biomolecular extractions

For biomolecular extractions on the adherent Caco-2 cells, the gasket–membrane assemblies were first washed in a 0.9% (w/v) NaCl solution. The cells were then immediately treated with 800 μl of an ice-cold 1:1 methanol:water (v/v) solution. Subsequently, the cells were disrupted and detached from the membranes using a plastic scrapper (VWR). The methanol:water suspension containing cells was then placed into a 2-ml sample tube and 400 μl of ice-cold chloroform were added. The sample mixtures were briefly vortexed and centrifuged at 14,000 r.p.m. (21,475*g*) for 5 min at 4 °C to separate the polar and non-polar metabolite phases and to concentrate the interphase pellets that were subsequently used for the extraction of biomacromolecules (RNA, DNA and proteins)^36^. The Caco-2 interphase pellets were then processed using the Qiagen AllPrep DNA/RNA/Protein Mini Kit^36^. The fractions of DNA, RNA and proteins fractions were sequentially isolated and snap-frozen. All extracts (except DNA fractions) were preserved at −80 °C until further analysis.

For the biomolecular extractions on the microbial cell contingents, the microbial cells were immediately disrupted and detached from the semi-permeable membranes using a plastic scrapper (VWR) and placed in a 0.9% (w/v) NaCl solution. This solution was then placed in a 2-ml sample tube and centrifuged at 4,000 r.p.m. (1,753*g*) for 10 min at 4 °C to pellet the bacteria. The supernatant was discarded and the bacterial pellet was snap-frozen. The bacterial pellet was then lysed using a Precellys lysis kit, and polar and non-polar metabolites fractions were obtained^36^. The interphase pellet was subsequently used for the extraction of biomolecules (DNA, RNA and proteins)^36^. The bacterial consortium structures were resolved by amplifying the V4 region of the 16S rRNA gene using primers 515F and 805R (515F_ GTGBCAGCMGCCGCGGTAA ; 805R_ GACTACHVGGGTATCTAATCC )^59^,^60^ to quantify the relative abundances of the two bacterial species. Resultant sequences were analysed using the 16S Metagenomics app on the Illumina basespace.

The intracellular (Caco-2 and bacteria) polar metabolite fractions were prepared for gas chromatography–mass spectrometry (GC–MS) analyses by pipetting 2 × 300 μl of polar fractions into 0.5-ml glass vials (Chromatographie Zubehör Trott) and dried using a speedvac. All vials were capped and stored at −80 °C until GC–MS analyses.

### Metabolomic analyses

Metabolites derivatisation was performed using a multipurpose sampler (Gerstel). Dried polar metabolite extracts from the Caco-2 cells, LGG or LGG and B. *caccae* were dissolved in 15 μl pyridine, containing 20 mg ml^−1^ methoxyamine hydrochloride, at 40 °C for 60 min while shaking. Following the addition of 15 μl *N*-methyl-*N*-trimethylsilyl-triflouroacetamide, the samples were incubated at 40 °C for 30 min under continuous shaking. GC–MS analyses were performed using an Agilent 7890 A GC coupled to an Agilent 5975C mass selective detector. A sample volume of 1 μl was injected into a split/splitless inlet operating in split (3:1) or splitless mode at 270 °C. The gas chromatograph was equipped with a 30-m DB-35MS capillary column and a 5-m DuraGuard capillary in front of the analytical column. Helium was used as the carrier gas with a constant flow rate of 1.0 ml min^−1^. The GC oven temperature was kept constant at 80 °C for 6 min and then increased to 300 °C at 6 °C min^−1^. After 10 min, the temperature was increased at a rate of 10 °C min^−1^ followed by a constant temperature period at 325 °C for 4 min. The total run time was 60 min. The transfer line temperature was set to constant 280 °C. The mass selective detector was operating under electron ionisation at 70 eV. The MS source was held at 230 °C and the quadrupole at 150 °C. Full-scan mass spectra were acquired from *m*/*z* 70 to *m*/*z* 800. An alkane mix was run with every experimental sequence to provide retention index calibration for the experimental samples. All GC–MS chromatograms were processed using the MetaboliteDetector software^61^.

### Microarray analyses

RNA extracts were prepared using the Affymetrix WT PLUS Reagent Kit (Affymetrix). RNA quality and quantity were assessed using a 2100 Agilent Bioanalyzer (Agilent) and a NanoDrop instrument (Thermo Scientific), respectively. For gene expression analysis, 100 ng of total RNA was used in conjunction with the Affymetrix standard protocol for Human Transcriptome Arrays 2.0 (Affymetrix Inc.). For the miRNAs analysis, 1 μg of total RNA was analysed using the FlashTag Biotin HSR RNA labelling kit for the Affymetrix Genechips miRNA 4.0 microarrays (Affymetrix Inc.).

### RT–qPCR validation of selected differentially expressed genes

Total RNA was extracted from Caco-2 cells co-cultured with LGG grown under anaerobic conditions in the HuMiX device as well as from Caco-2 cells grown under control conditions (Supplementary Fig. 3) using the Qiagen AllPrep DNA/RNA/Protein Mini Kit (Qiagen). Complementary DNA (cDNA) synthesis was carried out on the extracted total RNA using the SuperScript III First-Strand Synthesis System (Life Technologies, Europe). Equal amounts of cDNA were amplified in combination with sequence-specific forward and reverse primers (Eurogentec). The amplification reaction took place in a total volume of 20 μl of reaction mixture, whereby primers (Supplementary Table 11) were added at a concentration of 10 μM, 10 μl of iQ SYBR Green Supermix (Bio-Rad) and the remaining volume was adjusted for cDNA amount with water. To normalise the messenger RNA expression for each analysed sample, the human house-keeping gene L27 was amplified using the same reaction conditions. Real-time PCR was carried out on a LightCycler 480 Real-Time PCR System (Roche) using a denaturation step of 95 °C for 3 min followed by 45 cycles of 95 °C for 30 s, 60 °C for 30 s and 72 °C for 30 s, respectively. *C*_t_ values were obtained using automatic baseline and threshold settings provided by the LightCycler 480 Software, Version 1.5. Data were analysed and normalised using the ‘advanced relative quantification' method. Individual targets were analysed in three biological replicates and represented as a mean. Statistical significance was calculated using a paired Student's *t*-test.

### Data analyses

The microarray gene expression data were pre-processed using the GC-RMA procedure for background correction, quantile normalisation and probe replicate summarisation implemented in the gcrma R-package^62^. All statistical analyses were performed in the R Statistical Programming Environment^63^ and all differential expression analyses were carried out using the R-package limma^64^. Differential expression of genes between anaerobic co-cultures, aerobic co-cultures and their corresponding control samples were analysed using the empirical Bayes moderated *t*-statistic^65^. To account both for significance and effect size, the final ranking of genes was determined using the *π*-value, a statistic combining the *P* value significance and logarithmic FC (log-FC) into a single score^66^. Only genes with known functional annotations were considered for further analysis. Genes with opposing alteration patterns between co-culture and control samples under the different experimental conditions (Supplementary Fig. 3) were identified and scored as follows: first, the *π*-value for differential expression between co-culture and control samples was determined for each gene as described above, once for anaerobic samples and once for aerobic samples. Next, the genes in the two *π*-value rankings were filtered, such that only genes for which the sign of the log-FC differed between anaerobic and aerobic samples were retained. The final ranking for these genes was obtained from the sum of ranks for the two *π*-value rankings (that is, sorting the genes by decreasing *π*-values for both anaerobic and aerobic samples and summing up the ranks for each gene). Genes with shared expression profiles were determined analogously, only changing the above procedure by filtering such that only genes were retained for which the sign of the log-FC was the same for anaerobic and aerobic samples.

For the analysis of altered cellular pathways, the GeneGo MetaCore software suite was used with the differential expression statistics from the empirical Bayes moderated *t*-statistic as input (see details above). The genes were pre-filtered using a *P* value significance threshold (*P*<0.05) before applying the GeneGO analysis. To investigate the global gene expression alterations in biological processes of the gene ontology (GO) database, we determined all GO processes covered by at least 10 genes in the microarray data set and computed the median gene expression levels across process members for each of these GO processes. Significantly altered processes were then determined by applying the empirical Bayes moderated *t*-statistic again.

Finally, to visualise the expression of the genes or levels of the metabolites in the different experimental conditions, heat maps and dendrogram visualisations were generated using the gplots R-package ( http://cran.r-project.org/web/packages/gplots) and an average linkage hierarchical clustering with the Euclidean distance metric to determine the ordering of the genes and metabolites. Gene expression measurements were converted to *Z*-scores and visualised by a colour gradient, where blue colours represent lower *Z*-scores and yellow colours stand for higher *Z*-scores (the colour darkness in each heat map is proportional to the absolute *Z*-score). A different colour scheme was used for the metabolite plots (light blue and pink). All box plots were created using the standard R boxplot function^67^,^68^,^69^.

## Additional information

**Accession codes:** The metabolomic data have been deposited in the MetaboLights database under the accession code MTBLS328. The transcriptomic data have been deposited in the Gene Expression Omnibus (GEO) database under accession code GSE79383.

**How to cite this article:** Shah, P. *et al*. A microfluidics-based *in vitro* model of the gastrointestinal human–microbe interface. *Nat. Commun.* 7:11535 doi: 10.1038/ncomms11535 (2016).

## Supplementary Material

Supplementary Information Supplementary figures 1-8, supplementary tables 1-12, supplementary notes 1-8, supplementary references

## Figures and Tables

**Table 1:** Differentially expressed genes in Caco-2 cells following their HuMiX-based co-culture with LGG in comparison with *in vivo* data.

**Gene**	***In vitro***** HuMiX-based co-cultures**				***In vivo*** **data**			**Function**
	**LGG culture conditions**	**Expression**	**logFC**	***P*** **value**	**Subject**	**Expression**	**Reference**	
EGR1	Only differentially expressed when LGG growing under anaerobic conditions	Down	−2.56	0.0012	Human	Down	33	Transcription regulation, transcription factor activity for the regulation of cell proliferation and apoptosis, anti-cancer effect and IL-8 suppression
CCL2		Up	+1.51	0.0005	Human	Up	32	Chemotactic factor that attracts monocytes and basophils and binds to the chemokine receptors CCR2 and CCR4
SLC9A1		Down	−0.29	0.0168	Human	Down	32	Signal transduction, regulation of pH homeostasis, cell migration, cell volume and anti-inflammatory effect
UBD		Up	+0.61	0.0261	Human	Up	33	Proteasomal degradation, cytokine response, antimicrobial response and apoptosis
								
ELF3	Differentially expressed when LGG growing under both anaerobic and aerobic conditions	Up	+0.55	0.0022	Human & GF Piglet	Down	33,34	*ets* family member, epithelial-specific function, transcriptional mediator of angiogenesis during inflammation and epithelial cell differentiation
CXCR4		Up	+0.94	0.0019	Human	Up	33	Chemotaxis, cell arrest, angiogenesis, cell survival, maintenance of the epithelial barrier function and HIV-1 co-receptor
MYBL2		Down	−0.26	0.0446	Human	Down	33	Anti-apoptopic function, regulation of cell cycle and transcription, and epithelial cell differentiation
PIM1		Up	+1.13	0.0039	Human	Up	32	Cell survival, cell proliferation, cell growth and signal transduction
								
CYP1A1		Up	+0.82	0.0090	Human	Up	33	Drug metabolism and xenobiotic transformation
GADD45B		Up	+0.60	0.0134	Human	Down	33	Cell growth and apoptosis
PILRB		Down	−0.45	0.0374	Human	Up	33	Receptors involved in the regulation of the immune system and cellular signalling
CDK9		Down	−0.22	0.0467	Human	Up	33	Cell proliferation, regulation of the cell cycle, and transcription elongation factor
								
SOX4	Only differentially expressed when LGG growing under anaerobic conditions	Down	−0.31	0.0295	GF piglet	Unspecified	34	Transcription factor, regulation of cell fate and apoptosis pathway, and prognostic marker in colon and gastric cancer
CEBPA		Up	+0.55	0.0172	GF piglet	Unspecified	34	Transcription factor, cell cycle regulation and regulation of metallothioneins
PTGS2		Up	+1.13	0.0145	GF piglet	Down	34	Prostaglandin biosynthesis and metabolism, inflammation, and mitogenesis
IGFBP2		Up	+0.5	0.015	GF piglet	Up	34	Regulation of IGF-mediated growth and developmental rates
GSTA1		Down	−0.5	0.005	GF piglet	Down	34	Detoxification of carcinogens, drugs, environmental toxins and products of oxidative stress
CTNNB1		Up	+0.54	0.003	GF piglet	Up	34	Regulation of cell growth in addition to the creation and maintenance of epithelial layers
TPD52		Up	+0.42	0.008	GF piglet	Up	34	Molecular marker in human cancer and target for immunotherapy

FC, fold change; GF, germ free; LGG, *Lactobacillus rhamnosus* GG; IL-8, interleukin-8; IGF, insulin-like growth factor

References indicating the functions of the highlighted genes are provided in Supplementary Table 12.
