# Supplementary material for: A microfluidics-based in vitro model of the gastrointestinal human–microbe interface
Source: Nat Commun. 2016 May 11;7:11535. doi: 10.1038/ncomms11535 (PMC4865890; doi:10.1038/ncomms11535)
Supplement: Supplementary Information — Supplementary figures 1-8, supplementary tables 1-12, supplementary notes 1-8, supplementary references [file ncomms11535-s1.pdf]

Supplementary Information

Supplementary Figures

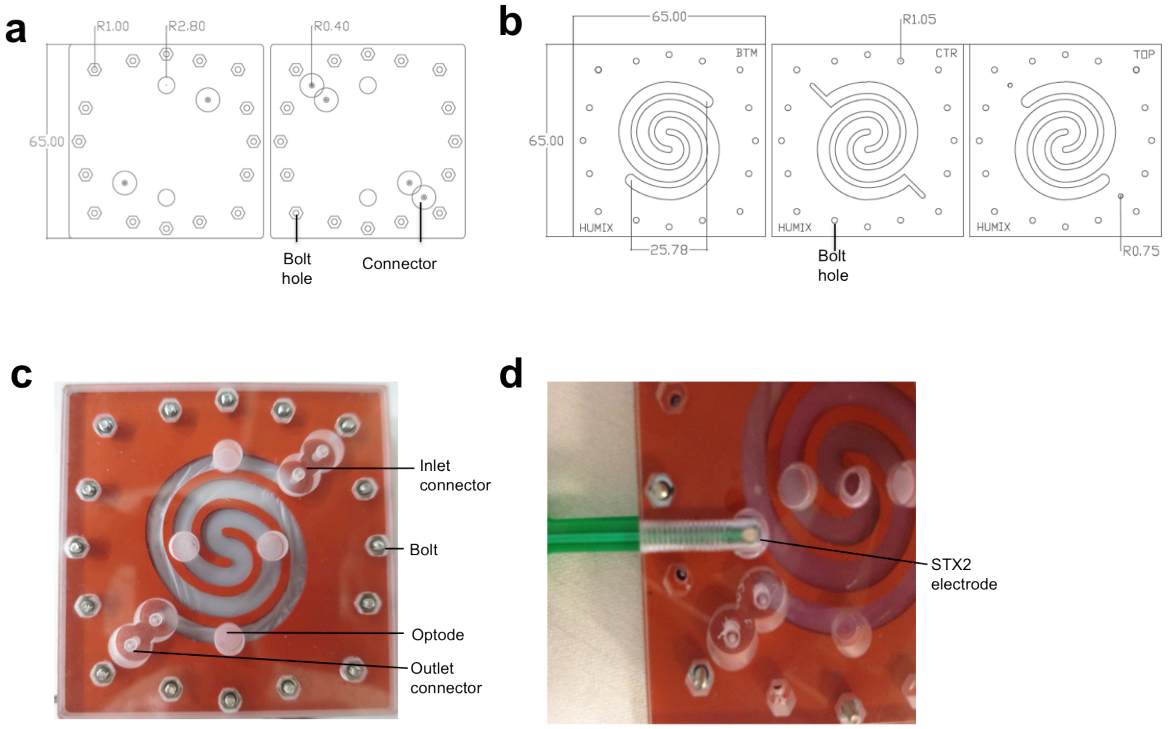

Supplementary Figure 1 │ Physical design parameters of the HuMiX device. (a) Polycarbonate enclosures (dimensions in mm). **(b)** Silicone rubber gaskets (dimensions in mm). **(c)** Top view of the HuMiX device showing optodes and connectors. **(d)** Measurement of transepithelial electrical resistance (TEER) in the HuMiX device using a specialized version of HuMiX, which allows the insertion of the standard STX2 chopstick electrode connected to the EVOM2 epithelial voltohmmeter.

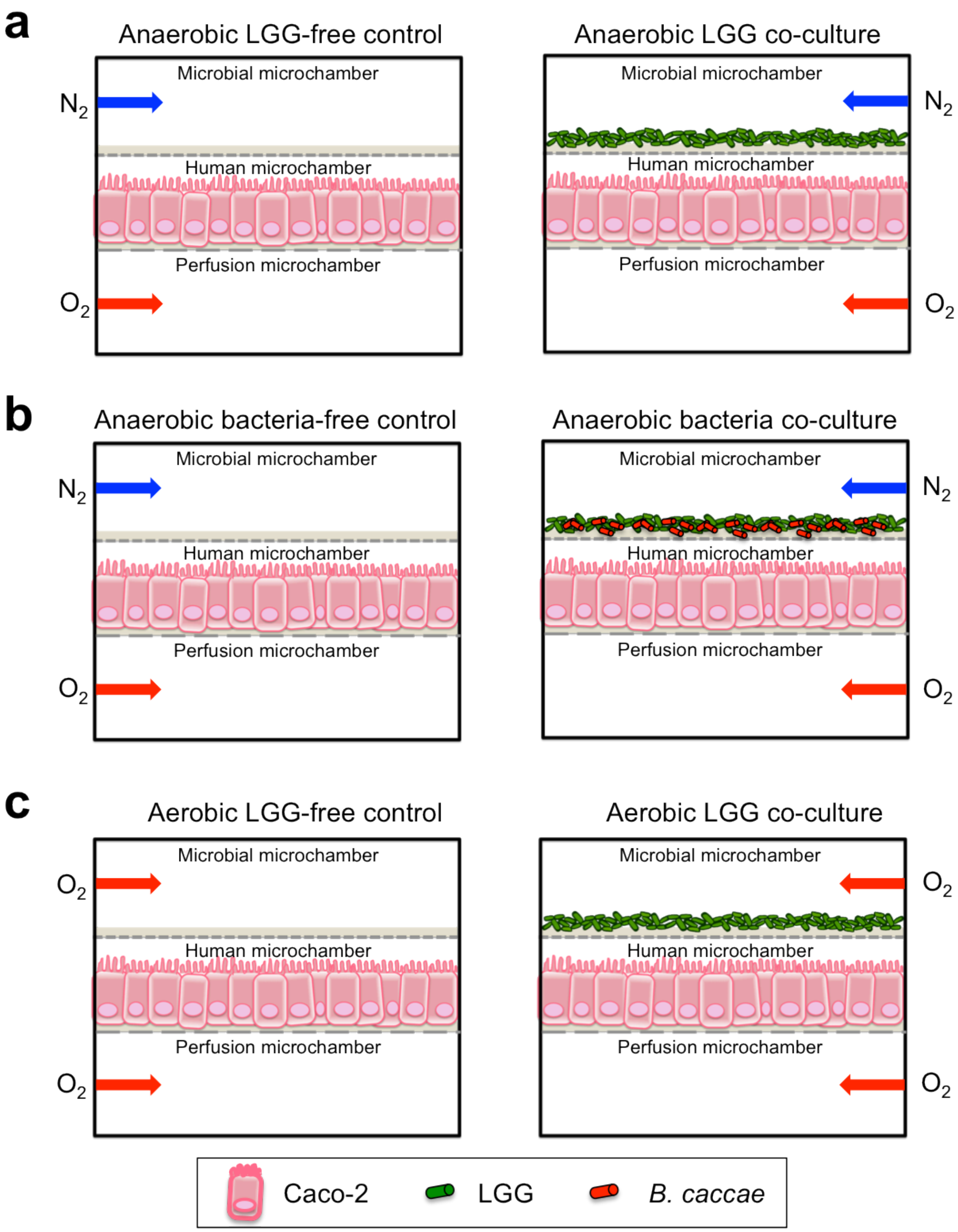

Supplementary Figure 2 │ Diagrammatic representation of the different co-culture experiments undertaken and the corresponding controls. (a) Caco-2 cells co-cultured with LGG grown under anaerobic conditions and the corresponding control. **(b)** Caco-2 cells co-cultured with LGG and *B. caccae* grown under anaerobic conditions and the corresponding control. **(c)** Caco-2 cells co-cultured with LGG grown under aerobic conditions and the corresponding control.

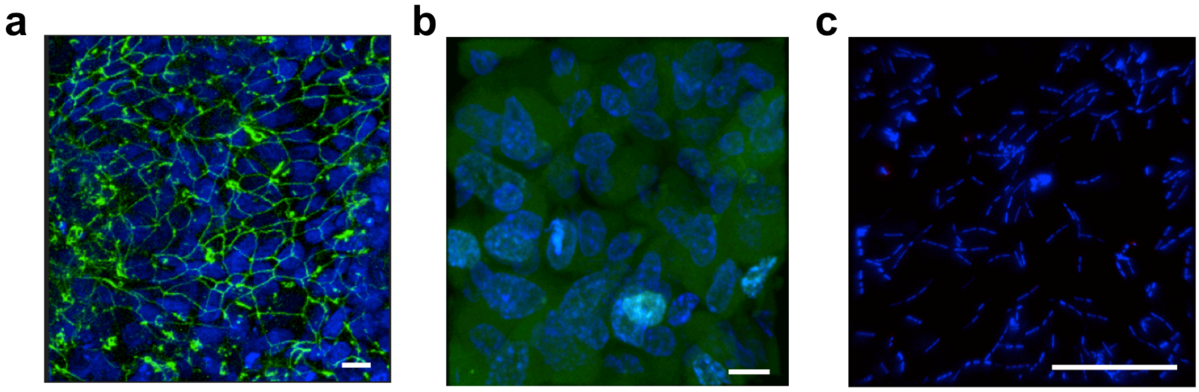

Supplementary Figure 3 │ *In vitro* co-culture of human epithelial cells (Caco-2) with a microbial consortium (LGG & *B. caccae*) inside the HuMiX device. (a) Immunofluorescence micrographs of the tight-junction protein occludin (green) in Caco-2 cells following 24 h of co-culture with LGG and *B. caccae* grown under anaerobic conditions. The cell nuclei are stained with DAPI and appear in blue. **(b)** Viability assessment of Caco-2 cells at 24 h post co-culture. The Caco-2 cells were stained using a live-dead stain and observed using a fluorescence microscope. The live cells appear in green, the dead cells appear in red and the nuclei in blue. **(c)** Viability assessment of microbial consortium (LGG & *B. caccae*) at 24 h post co-culture. All bacterial cells were stained with DAPI (blue) and dead bacteria were also stained positive with propidium iodide (red). (Scale bars in **(a)**, **(b)** and **(c)**: 10 µm)

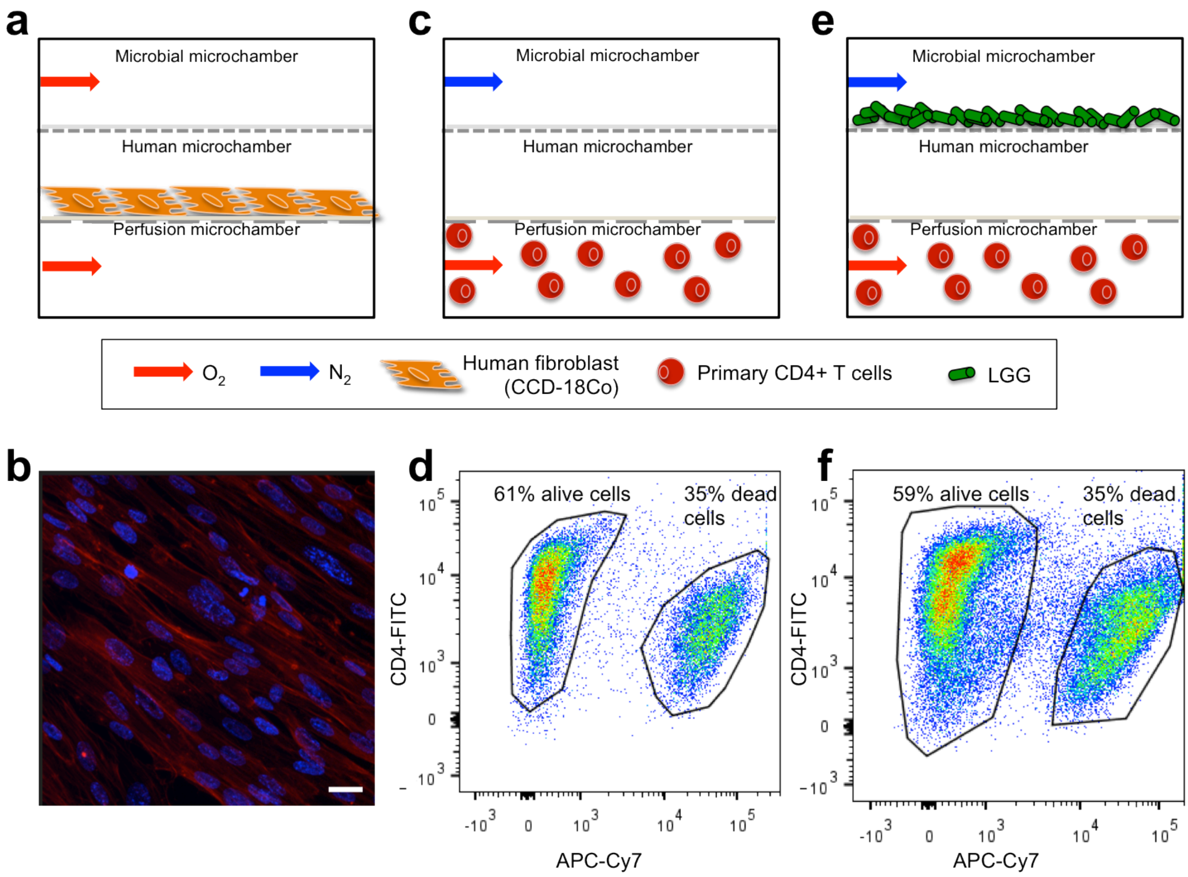

Supplementary Figure 4 | Growth of distinct human cell types in different microchambers of HuMiX. (a) Diagrammatic representation of the HuMiX model involving the culture of human colonic myofibroblasts in the human microchamber. **(b)** Immunofluorescence micrographs of the non-cancerous colonic cell line, CCD-18Co, cultured for 7 days in HuMiX. Filamentous actin was stained by Alexa Flour 568-Phalloidin (red) and the nuclei of the CCD-18Co are visualized in blue (DAPI). **(c)** Diagrammatic representation of the HuMiX model involving the culture of primary human CD4+ immune cells in the perfusion chamber. **(d)** Flow cytometry dot plot for the CD4+ T cells stained by using a FITC conjugated antibody directed against CD4 and using a LIVE/DEAD fixable Near IR (APC-Cy7) cell kit. **(e)** Diagrammatic representation of the HuMiX model involving the co-culture of primary human CD4+ immune cells in the perfusion chamber with LGG growing under anaerobic conditions. **(f)** Flow cytometry dot plot of CD4+ T cells, co-cultured for 24 h with LGG, stained by using a FITC conjugated antibody directed against CD4 and using a Live/Dead fixable Near IR (APC-Cy7) cell kit.

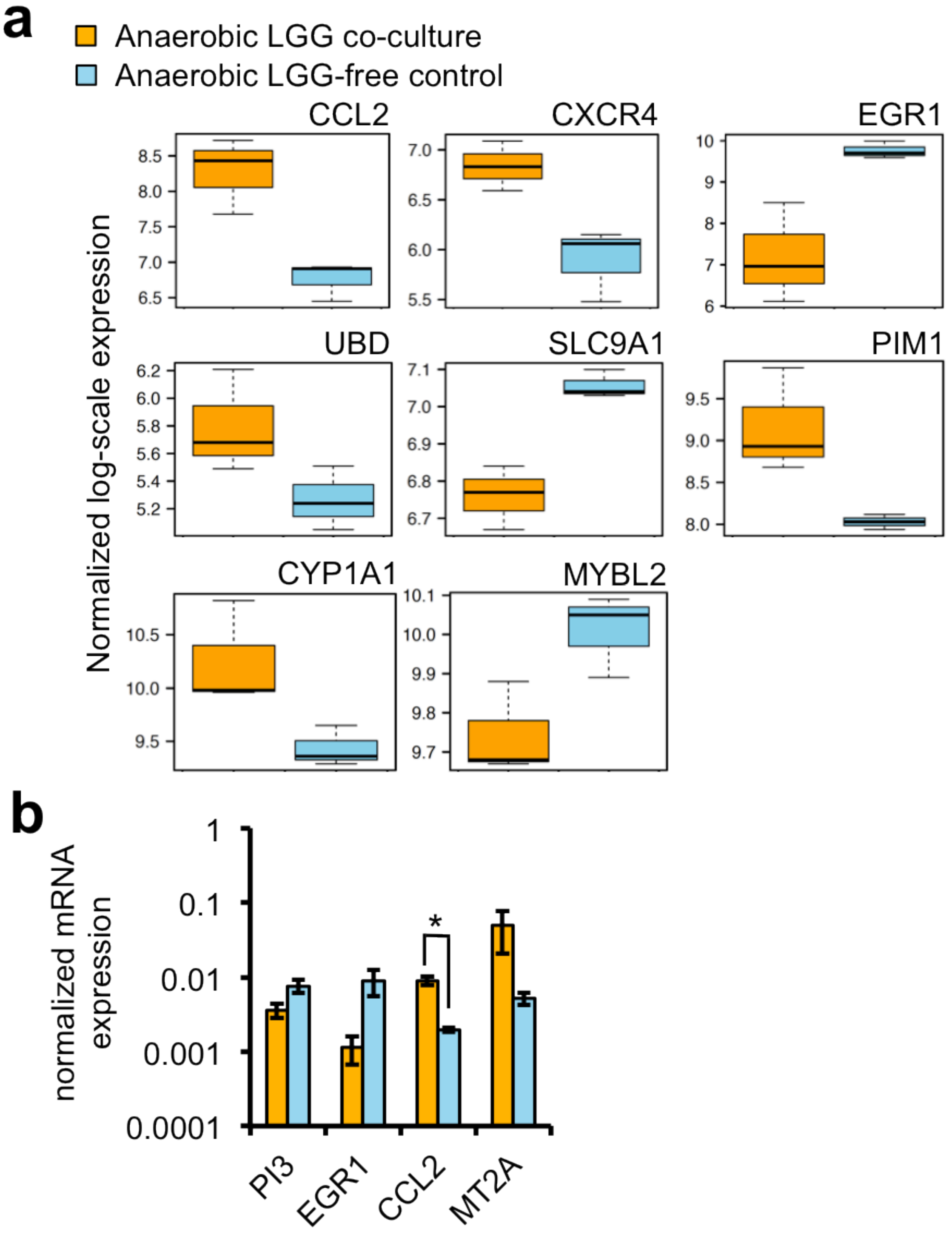

Supplementary Figure 5 | Differentially expressed genes in Caco-2 cells following their co-culture with LGG grown under anaerobic conditions. (a) Boxplots highlighting the differential expression of specific human genes of interest, i.e., previously described to be differentially expressed *in vivo* following the administration of LGG (n = 3). **(b)** Validation of four differentially expressed genes (the top two up- and downregulated genes) using RT-qPCR (n = 3). * indicates a statistically significant difference (paired Student's t-test; P = 0.029).

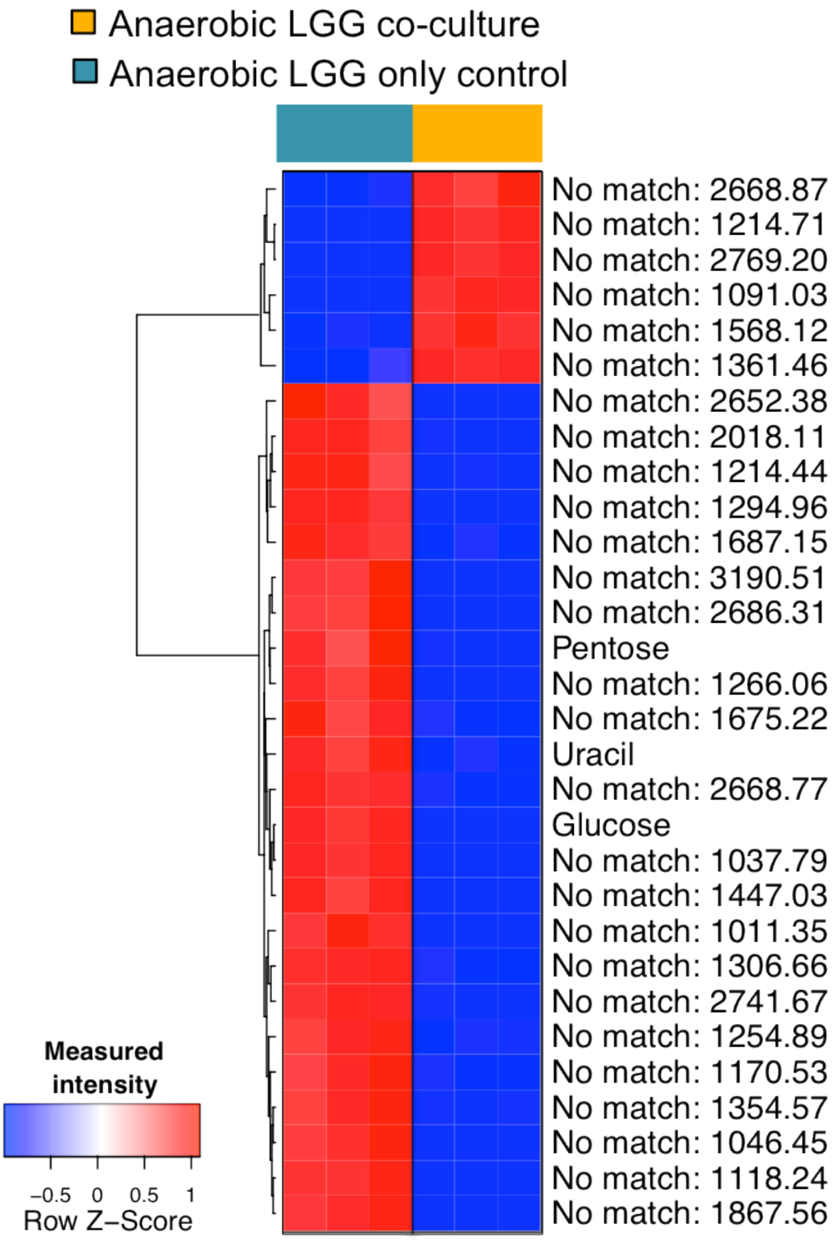

Supplementary Figure 6 │ Metabolomic profiling of the intracellular metabolites of LGG co-cultured with Caco-2 cells. Heatmap of top 30 statistically significant intracellular metabolites from LGG grown under anaerobic conditions when co-cultured with Caco-2 cells compared to mono-culture LGG controls (n = 3).

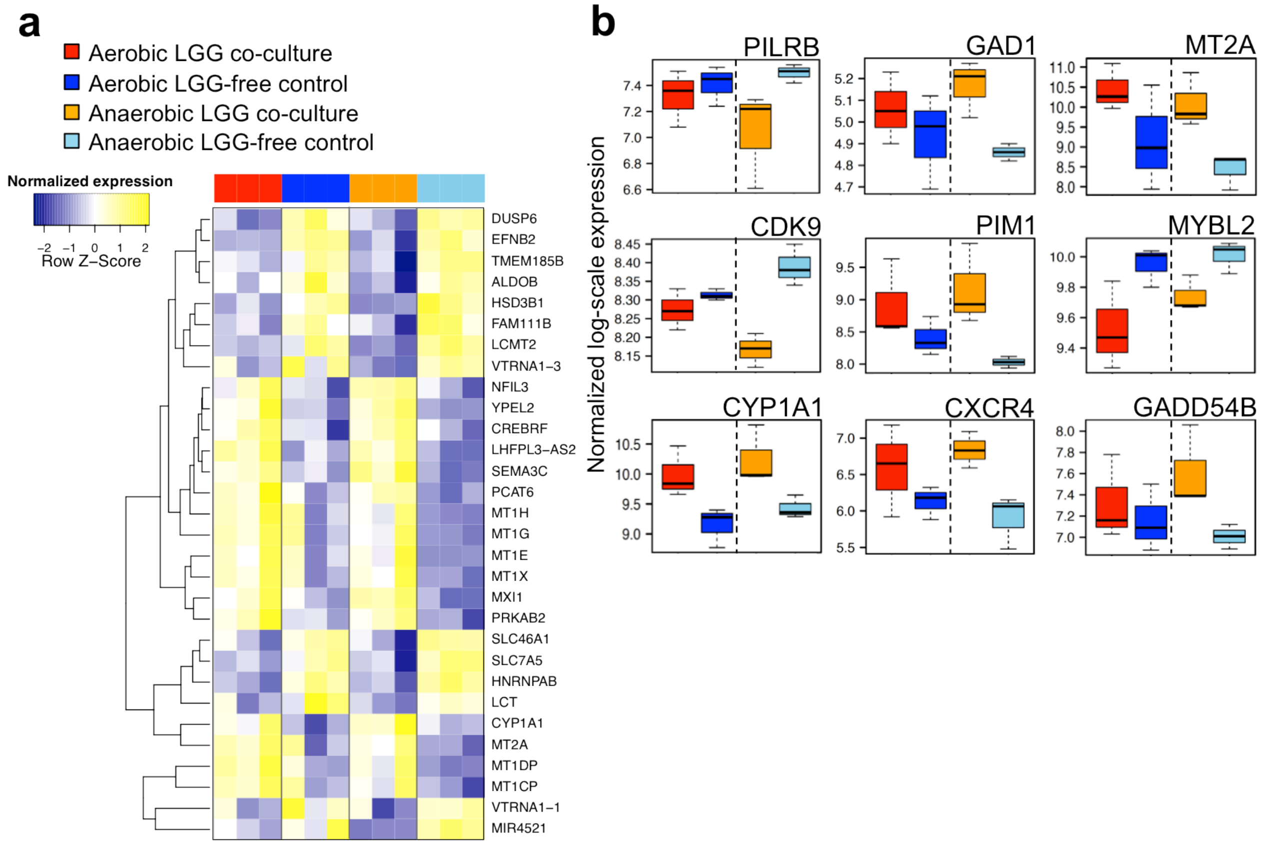

Supplementary Figure 7 │ Generic transcriptional response of Caco-2 cells co-cultured with LGG. (a) Heatmap of the top 30 differentially expressed genes showing a similar expression pattern (up- or down-regulation) when Caco-2 cells were co-cultured with LGG grown under either aerobic or anaerobic conditions. The threshold parameters used were FC > 1.5 and P < 0.05 (BtS), and the ranking is based on the pi-values. **(b)** Boxplots of physiologically relevant genes that exhibited analogous expression in Caco-2 cells following co-culture with LGG grown under either aerobic or anaerobic conditions.

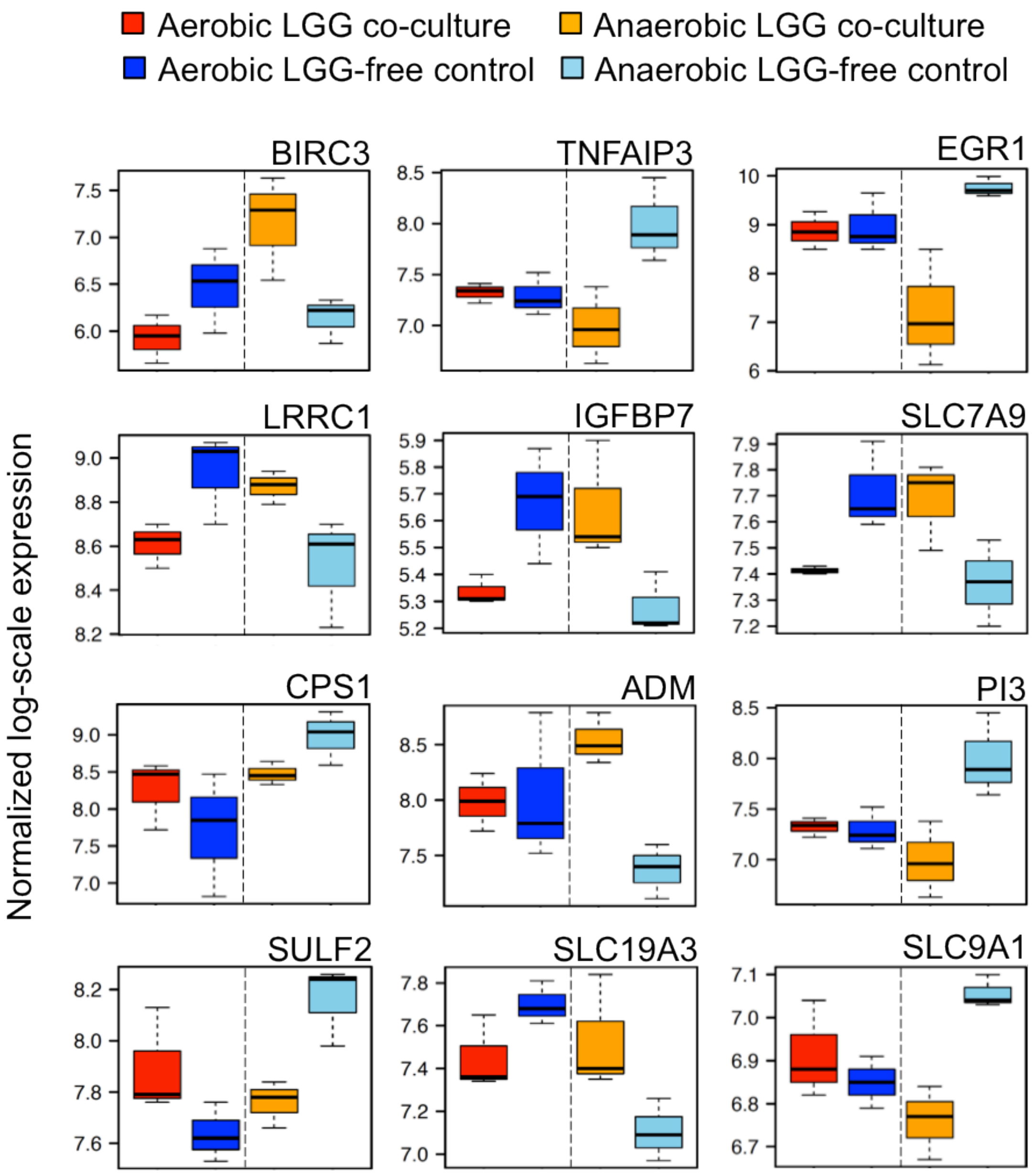

Supplementary Figure 8 │ Box plots of differentially expressed genes, highlighting the expression changes of the different Caco-2 cell genes following their co-culture with LGG grown under either anaerobic or aerobic conditions. Genes that are known to play physiologically relevant functions in relation to gastrointestinal health and disease are displayed. The ranking was based on the pi-values calculated using log-fold changes and *p*-values (B*t*S).

Supplementary Tables

Supplementary Table 1. Gene expression analysis of the genes exhibiting differential expression in Caco-2 cells co-cultured with LGG grown under anaerobic conditions versus their corresponding LGG-free controls. The table highlights the top 100 differentially expressed genes ordered according to their pi-values. The threshold parameters used were FC > 1.5 and $P < 0.05$ (B*t*S).

Rank	Symbol	logFC	P.Value
**1**	MIR4521	-1.810	1.97E-06
**2**	EGR1	-2.560	1.22E-03
**3**	MIR4668	1.833	3.16E-04
**4**	VTRNA1-3	-1.360	2.55E-05
**5**	CCL2	1.513	5.25E-04
**6**	MT2A	1.657	1.61E-03
**7**	LINC00641	-1.903	4.07E-03
**8**	ADM	1.170	1.30E-04
**9**	MT1X	1.787	3.19E-03
**10**	ANKRD37	1.170	1.62E-04
**11**	OTTHUMG00000019035	1.113	1.21E-04
**12**	PTGS2	1.130	1.46E-02
**13**	FGA	-1.413	8.84E-04
**14**	TNFAIP3	1.093	1.37E-04
**15**	MIR3941	1.040	1.00E-04
**16**	TRNAI6	-1.083	1.52E-04
**17**	HILPDA	1.030	1.03E-04
**18**	EFNA1	1.113	2.63E-04
**19**	MXI1	0.980	1.62E-04
**20**	OTTHUMG00000032445	-1.133	5.66E-04
**21**	OTTHUMG00000162597	0.940	1.41E-04
**22**	SLC17A1	0.990	1.17E-02
**23**	OTTHUMG00000162349	1.043	3.46E-04
**24**	MT1G	1.520	4.59E-03
**25**	MIR3143	-0.980	2.53E-04
**26**	ARRDC3	1.277	1.76E-03
**27**	HSD3B1	-0.913	1.98E-04
**28**	OTTHUMG00000162284	0.953	2.17E-02
**29**	ARHGAP19	0.833	8.84E-05
**30**	MIR4434	0.997	4.16E-04
**31**	SEMA3C	0.980	4.43E-04
**32**	HTR1D	-1.093	1.07E-03
**33**	SNORD14E	0.860	4.67E-02
**34**	HIST1H4H	-1.207	2.11E-03
**35**	YPEL2	0.883	2.91E-04
**36**	TIPARP	1.167	2.11E-03
**37**	LCMT2	-0.777	1.30E-04
**38**	DDIT4	0.837	2.68E-04
**39**	PDE10A	0.983	9.34E-04

**40**	STC1	0.827	1.75E-02
**41**	SAT1	0.980	9.21E-04
**42**	MT1JP	1.283	5.18E-03
**43**	ATMIN	-0.987	1.13E-03
**44**	ALDOB	-1.070	2.04E-03
**45**	HIST4H4	-0.710	1.09E-04
**46**	UGT2B17	-0.833	4.26E-04
**47**	OTTHUMG00000160261	1.163	3.96E-03
**48**	ZSCAN12P1	-1.203	4.77E-03
**49**	MIR3115	0.990	1.58E-03
**50**	MT1CP	1.383	1.25E-02
**51**	RBM3	0.777	3.12E-04
**52**	PIM1	1.130	3.95E-03
**53**	MT1E	1.247	6.71E-03
**54**	ABCA5	0.803	1.72E-02
**55**	NCOA5	-1.123	4.04E-03
**56**	HAL	-0.740	3.17E-04
**57**	HIST2H3D	-0.727	2.77E-04
**58**	OTTHUMG00000032525	-1.250	8.64E-03
**59**	CXCR4	0.940	1.93E-03
**60**	SNAI3-AS1	-1.020	3.36E-03
**61**	MT1H	1.053	4.16E-03
**62**	TFAP2C	0.873	1.57E-03
**63**	TMEM185B	-1.053	4.79E-03
**64**	PPP1R10	-1.327	1.46E-02
**65**	PPP1R10	-1.327	1.46E-02
**66**	PPP1R10	-1.327	1.46E-02
**67**	PI3	-1.003	4.15E-03
**68**	PTP4A1P7	0.750	2.33E-02
**69**	HIST2H4B	-0.983	3.93E-03
**70**	LHFPL3-AS2	0.753	7.37E-04
**71**	NAALAD2	0.767	8.72E-04
**72**	PCAT6	0.733	6.34E-04
**73**	MIR3136	0.737	1.73E-02
**74**	MT1F	0.970	3.85E-03
**75**	SNAR-A3	-0.717	5.55E-04
**76**	SNAR-A1	-0.713	5.59E-04
**77**	MIR22HG	-0.680	4.20E-04
**78**	METTL7B	-0.677	4.06E-04
**79**	HBP1	0.810	1.49E-03
**80**	MXD1	0.717	6.62E-04
**81**	WNK1	0.717	1.14E-02
**82**	BIRC3	1.013	5.77E-03
**83**	SERTAD2	0.763	1.09E-03
**84**	TMEM199	-0.687	5.29E-04
**85**	MIR4443	0.710	1.91E-02
**86**	LCT	-0.777	1.34E-03
**87**	HIST2H2BE	0.700	2.80E-02
**88**	SELRC1	-1.010	6.21E-03

**89**	KDM3A	0.697	1.11E-02
**90**	TNFRSF11B	-0.740	9.98E-04
**91**	CLK1	0.810	1.88E-03
**92**	C1orf116	-1.073	9.63E-03
**93**	NFIL3	0.800	2.02E-03
**94**	STK17B	0.743	1.26E-03
**95**	MT1L	0.687	3.25E-02
**96**	LOX	0.683	1.34E-02
**97**	SLC7A5	-0.917	4.60E-03
**98**	SNAR-A12	-0.647	5.11E-04
**99**	DKFZP667F0711	0.673	2.25E-02
**100**	TC01003017.hg.1	0.673	2.71E-02

Supplementary Table 2. Global pathway analysis (GeneGo MetaCore™ pathway analysis) of the genes that were differentially expressed in Caco-2 cells co-cultured with LGG under anaerobic conditions versus their corresponding LGG-free controls. The highlighted pathways are the top 50 altered pathways that are differentially expressed. The threshold parameters used were FC > 1.5 and $P < 0.01$ (B*t*S). 'Total' depicts the total number of genes that are linked to the indicated pathway, 'In data' highlights the number of genes linked to the indicated pathway that were found to be differentially expressed, and 'FDR' presents the False Discovery Rate.

Rank	Enriched Pathways	Total	In Data	FDR
**1**	Cytoskeleton remodeling_TGF, WNT and cytoskeletal remodeling	111	106	8.127E-21
**2**	Cytoskeleton remodeling_Cytoskeleton remodeling	102	95	1.667E-16
**3**	Development_Regulation of cytoskeleton proteins in oligodendrocyte differentiation and myelination	58	58	2.105E-14
**4**	Development_Regulation of epithelial-to-mesenchymal transition (EMT)	64	63	2.105E-14
**5**	Cell adhesion_Chemokines and adhesion	100	91	2.110E-14
**6**	Transport_Clathrin-coated vesicle cycle	71	68	1.169E-13
**7**	Development_WNT signaling pathway. Part 2	53	53	2.402E-13
**8**	Immune response_HSP60 and HSP70/ TLR signaling pathway	54	53	4.877E-12
**9**	Neurophysiological process_Dynein-dynactin motor complex in axonal transport in neurons	54	53	4.877E-12
**10**	Development_Differentiation of white adipocytes	53	52	8.188E-12
**11**	Development_TGF-beta receptor signaling	50	49	4.819E-11
**12**	Development_EGFR signaling pathway	71	65	1.135E-10
**13**	Signal transduction_JNK pathway	42	42	1.499E-10
**14**	Development_Cytokine-mediated regulation of megakaryopoiesis	57	54	1.951E-10
**15**	Immune response_Role of PKR in stress-induced antiviral cell response	57	54	1.951E-10
**16**	Development_TGF-beta-dependent induction of EMT via MAPK	47	46	2.135E-10
**17**	Breast cancer (general schema)	41	41	2.175E-10
**18**	Immune response_Naive CD4+ T cell differentiation	46	45	3.529E-10
**19**	Ovarian cancer (main signaling cascades)	64	59	3.763E-10
**20**	Neurophysiological process_Receptor-mediated axon growth repulsion	45	44	5.901E-10
**21**	Development_BMP7 in brown adipocyte differentiation	39	39	6.054E-10
**22**	Regulation of degradation of deltaF508-CFTR in CF	39	39	6.054E-10
**23**	Immune response_CCL2 signaling	54	51	7.417E-10
**24**	Transcription_N-CoR/ SMRT complex-mediated epigenetic gene silencing	49	47	8.766E-10
**25**	Transcription_Sin3 and NuRD in transcription regulation	44	43	8.766E-10
**26**	Apoptosis and survival_Role of PKR in stress-induced apoptosis	53	50	1.135E-09
**27**	Cell cycle_Influence of Ras and Rho proteins on G1/S Transition	53	50	1.135E-09
**28**	Immune response_Oncostatin M signaling via MAPK in human cells	37	37	1.653E-09
**29**	Cell adhesion_Role of tetraspanins in the integrin-mediated cell adhesion	37	37	1.653E-09
**30**	Transcription_Sirtuin6 regulation and functions	64	58	2.157E-09
**31**	Apoptosis and survival_Anti-apoptotic TNFs/NF-kB/Bcl-2 pathway	42	41	2.433E-09
**32**	Cytoskeleton remodeling_Keratin filaments	36	36	2.599E-09
**33**	Immune response_IL-9 signaling pathway	36	36	2.599E-09
**34**	Upregulation of MITF in melanoma	36	36	2.599E-09
**35**	G-protein signaling_Regulation of RAC1 activity	36	36	2.599E-09
**36**	Development_Oligodendrocyte differentiation from adult stem cells	51	48	2.634E-09

**37**	Some pathways of EMT in cancer cells	51	48	2.634E-09
**38**	Ligand-independent activation of Androgen receptor in Prostate Cancer	67	60	2.634E-09
**39**	Development_Regulation of lung epithelial progenitor cell differentiation	41	40	3.585E-09
**40**	Development_EPO-induced Jak-STAT pathway	35	35	4.097E-09
**41**	Immune response_Oncostatin M signaling via MAPK in mouse cells	35	35	4.097E-09
**42**	Development_TGF-beta-dependent induction of EMT via SMADs	35	35	4.097E-09
**43**	Development_GM-CSF signaling	50	47	4.097E-09
**44**	Immune response_Th1 and Th2 cell differentiation	40	39	5.756E-09
**45**	Development_Astrocyte differentiation from adult stem cells	40	39	5.756E-09
**46**	NF-AT signaling in cardiac hypertrophy	65	58	6.316E-09
**47**	Immune response_IL-2 activation and signaling pathway	49	46	6.454E-09
**48**	G-protein signaling_RhoA regulation pathway	34	34	6.454E-09
**49**	Chemotaxis_CXCR4 signaling pathway	34	34	6.454E-09
**50**	Development_Epigenetic and transcriptional regulation of oligodendrocyte precursor cell differentiation and myelination	34	34	6.454E-09

Supplementary Table 3. Gene Ontology (GO)-based pathway analysis highlighting altered biological processes in Caco-2 cells co-cultured with LGG growing under anaerobic conditions compared to LGG-free anaerobic controls. In addition to the top 100 altered GO processes, the statistically significantly altered processes that exhibit altered expression in our data compared to the available *in vivo* data from human subjects following the administration of LGG are highlighted. For our investigation, we only included the GO processes covered by at least 10 genes in the microarray dataset, and we then computed the median gene expression levels across the process members for each of the identified GO processes. The statistical significance was ascertained using the empirical Bayes t statistic (B*t*S).

Rank	Pathway	logFC	P.Value
**1**	GO:0042149~cellular response to glucose starvation	0.550	1.58E-05
**2**	GO:0071276~cellular response to cadmium ion	0.570	2.71E-04
**3**	GO:0032026~response to magnesium ion	-0.653	4.43E-04
**4**	GO:0021756~striatum development	0.430	5.80E-04
**5**	GO:0006754~ATP biosynthetic process	0.443	6.37E-04
**6**	GO:0019216~regulation of lipid metabolic process	0.363	7.49E-04
**7**	GO:0048617~embryonic foregut morphogenesis	0.400	8.87E-04
**8**	GO:0035924~cellular response to vascular endothelial growth factor stimulus	0.410	1.01E-03
**9**	GO:0008334~histone mRNA metabolic process	0.305	1.07E-03
**10**	GO:0045725~positive regulation of glycogen biosynthetic process	-0.523	1.42E-03
**11**	GO:0045070~positive regulation of viral genome replication	0.353	1.66E-03
**12**	GO:0045907~positive regulation of vasoconstriction	0.530	1.71E-03
**13**	GO:1900740~positive regulation of protein insertion into mitochondrial membrane involved in apoptotic signaling pathway	-0.297	1.89E-03
**14**	GO:0032259~methylation	-0.420	2.03E-03
**15**	GO:0002026~regulation of the force of heart contraction	-0.297	2.20E-03
**16**	GO:0005666~DNA-directed RNA polymerase III complex	-0.353	2.63E-03
**17**	GO:0008831~dTDP-4-dehydrorhamnose reductase activity	-0.295	2.68E-03
**18**	GO:0019725~cellular homeostasis	0.340	2.80E-03
**19**	GO:0070411~I-SMAD binding	0.330	2.86E-03
**20**	GO:0045921~positive regulation of exocytosis	-0.303	2.91E-03
**21**	GO:0030214~hyaluronan catabolic process	-0.277	2.97E-03
**22**	GO:0005916~fascia adherens	0.400	3.37E-03
**23**	GO:0008235~metalloexopeptidase activity	0.265	3.38E-03
**24**	GO:0003333~amino acid transmembrane transport	-0.257	3.64E-03
**25**	GO:0043252~sodium-independent organic anion transport	-0.300	3.89E-03
**26**	GO:0005605~basal lamina	0.267	3.99E-03
**27**	GO:0046627~negative regulation of insulin receptor signaling pathway	-0.350	4.49E-03
**28**	GO:0004016~adenylate cyclase activity	-0.267	4.50E-03
**29**	GO:0009950~dorsal/ventral axis specification	0.330	4.51E-03
**30**	GO:0048715~negative regulation of oligodendrocyte differentiation	0.350	4.94E-03
**31**	GO:0051393~alpha-actinin binding	-0.323	5.09E-03
**32**	GO:0008239~dipeptidyl-peptidase activity	0.385	5.14E-03
**33**	GO:0051019~mitogen-activated protein kinase binding	0.293	5.25E-03
**34**	GO:0004526~ribonuclease P activity	-0.313	5.34E-03

**35**	GO:0045792~negative regulation of cell size	-0.300	5.37E-03
**36**	GO:0010842~retina layer formation	-0.260	5.86E-03
**37**	GO:0016779~nucleotidyltransferase activity	-0.240	5.96E-03
**38**	GO:0000178~exosome (RNase complex)	-0.308	6.19E-03
**39**	GO:0007040~lysosome organization	-0.227	6.31E-03
**40**	GO:0006520~cellular amino acid metabolic process	-0.228	6.65E-03
**41**	GO:0001106~RNA polymerase II transcription corepressor activity	-0.233	7.29E-03
**42**	GO:0050982~detection of mechanical stimulus	-0.235	7.44E-03
**43**	GO:0006183~GTP biosynthetic process	-0.212	7.49E-03
**44**	GO:0090307~spindle assembly involved in mitosis	0.392	7.59E-03
**45**	GO:0006261~DNA-dependent DNA replication	-0.253	8.38E-03
**46**	GO:0045926~negative regulation of growth	0.807	8.48E-03
**47**	GO:0042254~ribosome biogenesis	-0.223	9.11E-03
**48**	GO:0050427~3'-phosphoadenosine 5'-phosphosulfate metabolic process	-0.237	9.37E-03
**49**	GO:0005123~death receptor binding	-0.250	9.41E-03
**50**	GO:0070530~K63-linked polyubiquitin binding	0.327	9.65E-03
**51**	GO:0004177~aminopeptidase activity	0.200	1.05E-02
**52**	GO:0007623~circadian rhythm	0.220	1.05E-02
**53**	GO:0046415~urate metabolic process	0.287	1.05E-02
**54**	GO:0006000~fructose metabolic process	-0.353	1.10E-02
**55**	GO:0032012~regulation of ARF protein signal transduction	0.233	1.10E-02
**56**	GO:0048666~neuron development	0.210	1.14E-02
**57**	GO:0004712~protein serine/threonine/tyrosine kinase activity	0.310	1.25E-02
**58**	GO:0006695~cholesterol biosynthetic process	-0.227	1.26E-02
**59**	GO:0030014~CCR4-NOT complex	0.247	1.30E-02
**60**	GO:0032147~activation of protein kinase activity	-0.383	1.30E-02
**61**	GO:0042059~negative regulation of epidermal growth factor receptor signaling pathway	0.193	1.31E-02
**62**	GO:0048487~beta-tubulin binding	0.240	1.31E-02
**63**	GO:0007141~male meiosis I	-0.235	1.33E-02
**64**	GO:0061077~chaperone-mediated protein folding	-0.320	1.45E-02
**65**	GO:0051272~positive regulation of cellular component movement	0.340	1.47E-02
**66**	GO:0044257~cellular protein catabolic process	-0.353	1.48E-02
**67**	GO:0001618~virus receptor activity	0.207	1.49E-02
**68**	GO:0051258~protein polymerization	-0.367	1.50E-02
**69**	GO:0060716~labyrinthine layer blood vessel development	0.213	1.53E-02
**70**	GO:0030057~desmosome	0.207	1.55E-02
**71**	GO:0030971~receptor tyrosine kinase binding	-0.273	1.60E-02
**72**	GO:0060968~regulation of gene silencing	-0.333	1.61E-02
**73**	GO:0006379~mRNA cleavage	-0.373	1.69E-02
**74**	GO:0015804~neutral amino acid transport	0.317	1.74E-02
**75**	GO:0015175~neutral amino acid transmembrane transporter activity	0.317	1.74E-02
**76**	GO:0031122~cytoplasmic microtubule organization	0.187	1.80E-02
**77**	GO:0009312~oligosaccharide biosynthetic process	-0.260	1.85E-02
**78**	GO:0005913~cell-cell adherens junction	0.237	1.92E-02
**79**	GO:0030259~lipid glycosylation	-0.288	1.92E-02
**80**	GO:0015020~glucuronosyltransferase activity	-0.288	1.92E-02
**81**	GO:0016558~protein import into peroxisome matrix	0.193	1.97E-02
**82**	GO:0071407~cellular response to organic cyclic compound	0.185	1.99E-02
**83**	GO:0048268~clathrin coat assembly	0.278	2.14E-02

**84**	GO:0034237~protein kinase A regulatory subunit binding	0.285	2.17E-02
**85**	GO:0031648~protein destabilization	0.210	2.17E-02
**86**	GO:0006493~protein O-linked glycosylation	-0.232	2.18E-02
**87**	GO:0034612~response to tumor necrosis factor	-0.363	2.20E-02
**88**	GO:0017127~cholesterol transporter activity	-0.350	2.20E-02
**89**	GO:0071222~cellular response to lipopolysaccharide	-0.260	2.27E-02
**90**	GO:0010508~positive regulation of autophagy	0.207	2.27E-02
**91**	GO:0005978~glycogen biosynthetic process	0.323	2.29E-02
**92**	GO:0043200~response to amino acid stimulus	0.187	2.29E-02
**93**	GO:0015721~bile acid and bile salt transport	-0.372	2.35E-02
**94**	GO:0032479~regulation of type I interferon production	-0.197	2.36E-02
**95**	GO:0008385~IkappaB kinase complex	0.170	2.39E-02
**96**	GO:0008080~N-acetyltransferase activity	0.252	2.41E-02
**97**	GO:0001824~blastocyst development	0.187	2.41E-02
**98**	GO:0043984~histone H4-K16 acetylation	-0.187	2.44E-02
**99**	GO:0040015~negative regulation of multicellular organism growth	0.270	2.49E-02
**100**	GO:0043162~ubiquitin-dependent protein catabolic process via the multivesicular body sorting pathway	0.167	2.52E-02
**116**	GO:0032481~positive regulation of type I interferon production	-0.183	3.23E-02
**117**	GO:0051592~response to calcium ion	-0.163	3.26E-02
**118**	GO:0006878~cellular copper ion homeostasis	-0.31	3.29E-02
**224**	GO:0051924~regulation of calcium ion transport	-0.133	7.15E-02
**259**	GO:0055072~iron ion homeostasis	0.126	8.56E-02

Supplementary Table 4. Metabolomic analysis of the intracellular metabolites of Caco-2 cells following 24 h of co-culture with LGG grown under anaerobic conditions (n=3). The table compares the concentration of the top 100 metabolites in control and co-culture experiments ordered according to their p-values. The threshold parameters used were $P < 0.05$ (S*t*T).

Metabolite	LGG O2-deficient co-culture			O2-deficient control			p-value
**No match: 2394.62**	3.11E-04	3.35E-04	3.56E-04	2.11E-04	2.47E-04	2.37E-04	7.79E-03
**No match: 1885.71**	2.39E-03	2.29E-03	3.08E-03	6.62E-04	1.01E-03	7.66E-04	2.71E-02
**Citric acid**	3.60E-03	3.39E-03	4.71E-03	8.60E-04	6.46E-04	2.25E-04	2.93E-02
**Fructose**	1.32E-02	5.50E-03	8.98E-03	3.37E-02	3.23E-02	2.33E-02	2.97E-02
**Fumaric acid**	1.38E-03	1.75E-03	1.43E-03	6.99E-04	4.51E-04	3.55E-04	3.07E-02
**Ornithine**	1.58E-03	7.74E-04	1.28E-03	2.15E-03	1.47E-03	1.63E-03	3.25E-02
**Phosphoric acid**	8.78E-02	1.37E-01	1.06E-01	3.11E-02	4.47E-02	5.30E-02	3.25E-02
**No match: 1600.67**	6.73E-04	7.45E-04	4.53E-04	1.16E-04	8.42E-05	1.18E-04	3.29E-02
**Unknown#sst cgl 090**	3.96E-04	6.65E-04	4.59E-04	3.18E-05	8.95E-05	1.55E-04	3.74E-02
**Adipic acid**	2.48E-04	2.45E-04	1.60E-04	1.40E-04	5.00E-05	4.58E-05	3.81E-02
**No match: 2326.37**	1.38E-04	1.14E-04	1.30E-04	3.62E-05	6.91E-05	5.03E-05	4.52E-02
**Unknown#sst cgl 008**	3.86E-03	8.08E-03	5.32E-03	4.84E-04	1.06E-03	1.93E-03	6.32E-02
**Isocitric acid**	3.60E-03	3.12E-03	5.52E-03	9.88E-04	6.46E-04	2.25E-04	6.37E-02
**No match: 1235.34**	9.05E-05	5.81E-05	3.74E-05	4.23E-05	2.32E-05	2.08E-05	6.80E-02
**No match: 1640.24**	2.86E-03	4.46E-03	1.78E-03	2.17E-04	3.96E-04	4.68E-04	7.78E-02
**No match: 1907.90**	4.88E-04	1.75E-04	3.87E-04	1.05E-03	6.05E-04	5.46E-04	8.38E-02
**4-Aminobutanoic acid**	8.22E-05	1.11E-04	5.29E-05	3.51E-05	4.33E-05	3.44E-05	9.05E-02
**Gluconic acid**	6.22E-04	2.14E-04	4.08E-04	9.52E-04	7.98E-04	5.88E-04	9.09E-02
**Erythronate**	4.73E-04	8.60E-04	5.95E-04	6.17E-04	9.04E-04	7.79E-04	9.66E-02
**Phosphoethanolamine**	1.76E-03	1.63E-03	9.85E-04	5.08E-04	1.01E-03	6.05E-04	1.02E-01
**Ribitol-Lu**	4.91E-04	3.58E-04	4.81E-04	6.59E-04	6.91E-04	5.77E-04	1.05E-01
**No match: 1756.24**	1.88E-05	1.05E-05	4.62E-05	4.65E-05	7.66E-05	6.88E-05	1.06E-01
**Aspartic acid**	2.93E-03	2.34E-03	3.28E-03	4.69E-04	1.70E-03	1.93E-03	1.07E-01
**Glycerol-3-phosphoric acid**	1.12E-02	2.46E-02	1.41E-02	4.20E-03	6.61E-03	8.09E-03	1.16E-01
**Creatinine**	2.50E-03	6.67E-03	6.00E-03	1.64E-03	1.64E-03	1.91E-03	1.19E-01
**Urea**	2.66E-03	4.63E-03	7.08E-03	1.17E-02	9.89E-03	8.98E-03	1.21E-01
**No match: 2035.50**	5.43E-05	3.99E-04	3.79E-04	6.58E-07	3.95E-05	9.39E-05	1.27E-01
**beta-Alanine**	1.44E-04	2.22E-04	1.21E-04	9.55E-05	9.90E-05	8.59E-05	1.28E-01
**Erythritol**	2.18E-03	1.92E-03	2.55E-03	3.56E-03	3.47E-03	2.76E-03	1.33E-01
**Isoleucine**	4.99E-04	2.66E-04	5.24E-04	9.39E-04	6.16E-04	5.80E-04	1.36E-01
**Galactose**	1.32E-03	1.47E-03	2.45E-03	1.09E-03	8.97E-04	1.30E-03	1.36E-01
**Cholesterol**	7.43E-04	2.71E-03	1.54E-03	3.90E-04	5.98E-04	3.33E-04	1.37E-01
**Pyrophosphoric acid**	3.72E-03	1.57E-02	9.29E-03	2.24E-04	2.58E-03	4.61E-03	1.44E-01
**Unknown#bth pae 011**	2.89E-04	1.39E-04	3.01E-04	5.59E-04	3.41E-04	3.25E-04	1.52E-01
**Unknown#bth pae 024**	2.79E-04	5.72E-04	3.43E-04	1.72E-04	1.87E-04	2.28E-04	1.57E-01
**Glucono lactone**	2.68E-03	1.31E-03	2.41E-03	3.53E-03	4.06E-03	3.11E-03	1.63E-01
**No match: 1560.73**	4.74E-05	1.84E-05	6.21E-05	7.50E-05	2.56E-04	1.69E-04	1.80E-01
**Uracil**	4.26E-05	1.99E-04	1.63E-04	7.19E-06	6.94E-05	1.35E-04	1.89E-01
**Norleucine**	9.99E-04	1.09E-03	1.06E-03	5.70E-04	9.01E-04	1.03E-03	1.96E-01
**No match: 2526.09**	2.48E-03	1.07E-03	1.14E-03	2.23E-04	7.00E-04	4.41E-04	1.97E-01
**Malic acid**	1.29E-03	1.92E-03	1.14E-03	1.07E-03	1.27E-03	1.07E-03	2.08E-01
**Unknown#bth pae 065**	2.65E-04	4.05E-06	1.29E-04	1.40E-04	1.78E-03	2.22E-03	2.13E-01
**Threonine**	8.43E-03	1.48E-02	1.25E-02	7.60E-03	8.75E-03	1.08E-02	2.17E-01
**Taurine**	3.87E-03	2.40E-02	1.01E-02	1.35E-04	9.14E-05	3.90E-03	2.17E-01
**No match: 2823.69**	1.25E-04	3.89E-04	7.07E-05	0.00E+00	2.15E-05	3.75E-05	2.21E-01
**Mannose**	7.33E-02	1.95E-02	7.63E-02	1.42E-01	9.55E-02	7.01E-02	2.21E-01
**Proline**	6.99E-04	5.63E-03	4.80E-03	8.90E-04	3.19E-03	2.91E-03	2.27E-01
**No match: 1155.89**	8.97E-04	2.29E-04	1.74E-04	2.76E-04	9.00E-05	7.48E-05	2.30E-01
**No match: 1957.19**	1.20E-04	1.48E-05	7.00E-05	1.25E-04	5.62E-05	1.79E-04	2.32E-01

**No match: 2245.13**	2.11E-04	2.06E-04	4.03E-04	2.39E-04	6.60E-04	5.58E-04	2.34E-01
**Serine**	4.15E-03	1.20E-02	1.29E-02	4.61E-03	6.94E-03	9.27E-03	2.39E-01
**No match: 2534.24**	4.42E-05	4.19E-05	2.23E-05	1.48E-05	1.38E-05	2.61E-05	2.42E-01
**No match: 1562.46**	1.68E-04	1.00E-03	6.57E-04	1.68E-04	1.99E-04	3.51E-04	2.55E-01
**Glucose**	4.26E-02	1.35E-02	3.69E-02	3.04E-02	9.05E-02	9.63E-02	2.68E-01
**Leucine**	4.44E-03	7.56E-03	9.48E-03	7.82E-03	1.21E-02	8.79E-03	2.69E-01
**5-Oxoproline**	5.93E-02	6.23E-02	8.19E-02	1.04E-01	8.96E-02	7.62E-02	2.74E-01
**Unknown#sst cgl D05**	1.20E-03	2.76E-04	2.86E-04	2.40E-04	1.48E-04	1.50E-04	2.77E-01
**Uridine**	6.88E-03	2.55E-03	6.99E-04	2.74E-05	6.10E-04	7.14E-04	2.89E-01
**Unknown#sst cgl 011**	4.43E-05	4.43E-05	3.50E-05	2.47E-04	2.18E-05	6.63E-04	2.93E-01
**Phosp(1gs)monomethylester**	2.74E-03	5.41E-03	5.00E-03	3.67E-03	1.32E-03	1.36E-03	2.93E-01
**Adenosine-5'-monophosp(1gs)**	0.00E+00	1.19E-04	7.26E-05	9.79E-06	1.87E-05	2.97E-05	2.97E-01
**Unknown#bth pae 026**	4.26E-05	4.36E-04	3.45E-05	5.72E-05	6.17E-04	8.25E-04	2.98E-01
**Benzoic acid**	1.12E-03	2.17E-04	2.31E-04	1.58E-03	2.72E-04	2.78E-04	3.04E-01
**Unknown#sst cgl 027**	1.40E-04	1.06E-04	4.07E-05	4.92E-05	3.99E-04	4.70E-04	3.09E-01
**Phosphoenolpyruvic acid**	1.91E-04	2.58E-04	7.74E-05	6.49E-05	1.12E-04	1.18E-04	3.20E-01
**No match: 1965.52**	2.81E-04	2.42E-03	4.20E-04	2.22E-04	1.43E-04	1.00E-04	3.33E-01
**Unknown#sst cgl 035**	3.94E-05	1.23E-04	1.17E-04	9.88E-05	1.19E-04	1.33E-04	3.35E-01
**No match: 1251.35**	9.50E-04	1.51E-04	1.39E-03	8.72E-04	5.19E-05	1.60E-04	3.43E-01
**Pantothenic acid**	9.44E-04	2.70E-03	1.77E-03	1.12E-03	1.28E-03	1.30E-03	3.44E-01
**Unknown#bth pae 046**	9.45E-05	3.26E-04	7.10E-05	5.24E-05	3.60E-05	7.11E-05	3.46E-01
**No match: 1988.71**	3.15E-03	5.87E-04	2.15E-03	2.56E-03	4.49E-03	3.45E-03	3.59E-01
**No match: 1714.34**	8.19E-05	1.94E-05	7.70E-05	2.67E-05	1.66E-04	2.18E-04	3.63E-01
**Inositol**	6.03E-02	1.49E-01	1.10E-01	7.80E-02	6.98E-02	7.43E-02	3.67E-01
**No match: 3280.72**	3.12E-05	6.54E-05	1.53E-04	3.01E-05	5.28E-05	1.03E-05	3.70E-01
**Phenylalanine**	7.50E-03	6.73E-03	9.05E-03	8.65E-03	9.91E-03	8.45E-03	3.72E-01
**2-Ketoglutaric acid**	2.55E-04	5.25E-04	3.37E-04	3.52E-04	2.17E-04	1.45E-04	3.80E-01
**Valine**	5.40E-03	8.86E-03	1.15E-02	8.35E-03	1.38E-02	9.96E-03	3.82E-01
**No match: 1940.12**	1.02E-04	5.78E-06	2.88E-05	4.40E-05	2.33E-04	1.35E-04	3.83E-01
**3-Phosphoglyceric acid**	3.94E-03	3.56E-03	1.32E-03	2.71E-03	1.42E-03	1.97E-03	3.85E-01
**Oxalic acid**	2.82E-02	3.26E-04	3.65E-04	5.29E-04	1.62E-04	2.76E-04	4.17E-01
**Unknown#sst cgl 082**	1.31E-03	1.46E-03	1.04E-03	1.47E-03	8.08E-04	8.33E-04	4.24E-01
**Carbonic acid**	1.18E-02	7.15E-03	9.50E-03	8.99E-03	1.64E-02	1.36E-02	4.24E-01
**Unknown#sst cgl 010a**	7.60E-05	7.76E-05	3.63E-05	6.10E-04	7.07E-05	4.06E-05	4.26E-01
**3-Hydroxybutyric acid**	1.09E-04	1.26E-04	1.48E-04	2.65E-04	1.49E-04	1.25E-04	4.36E-01
**No match: 1029.42**	3.40E-04	3.27E-06	6.95E-05	3.78E-05	4.70E-05	3.66E-05	4.52E-01
**No match: 2188.21**	2.33E-04	5.47E-05	2.67E-04	1.72E-04	4.61E-04	3.13E-04	4.52E-01
**Unknown#sst cgl 011**	2.03E-05	3.06E-05	2.15E-05	3.09E-04	2.21E-05	1.50E-05	4.53E-01
**Hexadecanoic acid**	2.34E-04	1.26E-03	1.02E-03	5.85E-04	5.59E-04	4.51E-04	4.54E-01
**Lysine**	3.97E-03	5.09E-04	1.92E-03	2.89E-03	3.30E-03	3.23E-03	4.67E-01
**No match: 1567.88**	9.64E-04	1.04E-04	3.11E-04	9.54E-05	2.39E-04	2.62E-04	4.87E-01
**No match: 1684.74**	9.27E-05	3.14E-05	3.31E-05	3.32E-05	1.04E-04	1.60E-04	4.89E-01
**Glycine**	7.80E-05	1.01E-02	5.96E-03	2.04E-03	3.81E-03	4.37E-03	4.96E-01
**Glutamic acid**	9.31E-03	5.12E-02	3.48E-02	2.24E-02	2.16E-02	2.22E-02	5.18E-01
**Unknown#bth pae 050**	7.01E-05	5.92E-05	9.83E-05	6.68E-05	7.35E-05	2.98E-05	5.27E-01
**Glycolic acid**	3.84E-03	2.29E-04	2.56E-04	4.67E-04	9.14E-04	2.12E-04	5.42E-01
**Glycerol**	3.30E-03	7.20E-02	5.22E-03	7.74E-03	5.64E-02	6.30E-02	5.52E-01
**Pyruvic acid**	1.12E-03	6.57E-04	2.63E-03	1.46E-03	2.32E-03	1.99E-03	5.64E-01
**No match: 1300.45**	8.11E-05	9.47E-05	8.87E-05	2.45E-04	7.66E-05	6.78E-05	5.66E-01
**Unknown#bth pae 013**	5.65E-04	8.57E-04	9.24E-04	1.81E-03	8.71E-04	6.14E-04	5.73E-01
**Sarcosine**	1.87E-04	3.68E-04	5.73E-05	1.70E-04	1.10E-04	1.40E-04	5.91E-01

Supplementary Table 5. Metabolomic analysis of the intracellular metabolites of LGG grown under anaerobic conditions following 24 h co-culture with Caco-2. The table compares the concentration of the top 100 metabolites in control and co-culture experiments ordered according to their p-values. The threshold parameters used were $P < 0.05$ (S*t*T).

Metabolite	Caco-2-free LGG control			LGG co-cultured with Caco-2			p-value
**No match: 2668.77**	5.01E+04	4.78E+04	4.85E+04	3.53E+03	1.83E+03	1.82E+03	2.85E-05
**No match: 2769.20**	0.00E+00	0.00E+00	0.00E+00	5.72E+04	5.56E+04	5.73E+04	9.75E-05
**No match: 1037.79**	3.14E+04	3.06E+04	3.17E+04	0.00E+00	0.00E+00	0.00E+00	1.23E-04
**No match: 1568.12**	6.69E+03	9.32E+03	7.85E+03	6.47E+04	6.87E+04	6.49E+04	1.35E-04
**Unknown#cgl**	2.11E+03	4.27E+03	1.94E+02	1.04E+05	1.02E+05	1.00E+05	1.48E-04
**No match: 1214.71**	0.00E+00	0.00E+00	0.00E+00	5.27E+04	5.09E+04	5.32E+04	1.74E-04
**No match: 1091.03**	3.39E+03	3.72E+03	3.85E+03	1.24E+05	1.30E+05	1.29E+05	2.03E-04
**Mannose**	2.74E+08	2.62E+08	2.75E+08	6.58E+05	4.42E+04	1.12E+05	2.39E-04
**No match: 1294.96**	1.13E+04	1.13E+04	1.08E+04	0.00E+00	0.00E+00	0.00E+00	2.77E-04
**No match: 1254.89**	1.73E+05	1.82E+05	1.86E+05	3.08E+04	3.86E+04	3.56E+04	3.60E-04
**No match: 2741.67**	4.75E+05	4.96E+05	4.89E+05	1.28E+04	2.14E+03	1.76E+03	4.05E-04
**No match: 2668.87**	5.25E+02	7.92E+02	3.89E+03	7.00E+04	6.68E+04	7.50E+04	4.62E-04
**No match: 1118.24**	8.99E+05	9.01E+05	9.61E+05	0.00E+00	0.00E+00	0.00E+00	4.86E-04
**No match: 1867.56**	2.22E+04	2.28E+04	2.39E+04	0.00E+00	0.00E+00	0.00E+00	5.19E-04
**No match: 2018.11**	1.09E+06	1.08E+06	1.00E+06	3.38E+04	2.96E+04	1.94E+04	5.54E-04
**No match: 1447.03**	2.26E+04	2.10E+04	2.27E+04	0.00E+00	0.00E+00	0.00E+00	6.60E-04
**No match: 1011.35**	2.71E+05	2.96E+05	2.78E+05	0.00E+00	0.00E+00	0.00E+00	6.77E-04
**No match: 1306.66**	1.66E+05	1.68E+05	1.70E+05	3.28E+04	2.58E+04	2.43E+04	7.59E-04
**No match: 1675.22**	1.84E+05	1.63E+05	1.76E+05	1.19E+04	3.25E+03	0.00E+00	8.84E-04
**No match: 1354.57**	9.57E+04	1.01E+05	1.06E+05	1.31E+03	0.00E+00	1.07E+03	9.59E-04
**No match: 1046.45**	2.19E+06	2.28E+06	2.44E+06	3.05E+03	0.00E+00	5.33E+03	9.82E-04
**No match: 1266.06**	7.85E+06	7.50E+06	8.37E+06	7.50E+03	1.72E+03	3.98E+03	1.03E-03
**No match: 1687.15**	1.10E+06	1.05E+06	1.01E+06	3.03E+04	9.43E+04	2.90E+04	1.16E-03
**No match: 1214.44**	8.62E+04	8.61E+04	7.76E+04	6.71E+03	7.45E+03	6.85E+03	1.34E-03
**No match: 3190.51**	4.49E+05	4.43E+05	5.00E+05	0.00E+00	0.00E+00	0.00E+00	1.55E-03
**No match: 1170.53**	8.30E+04	8.84E+04	9.26E+04	3.69E+03	0.00E+00	0.00E+00	2.02E-03
**No match: 1361.46**	2.27E+04	2.23E+04	7.86E+04	4.47E+05	4.38E+05	4.44E+05	2.09E-03
**Mannose**	1.21E+08	1.22E+08	1.40E+08	1.47E+05	1.16E+04	2.50E+04	2.28E-03
**Uracil**	2.32E+05	2.21E+05	2.42E+05	0.00E+00	1.48E+04	0.00E+00	2.31E-03
**No match: 2652.38**	2.07E+04	1.91E+04	1.74E+04	0.00E+00	0.00E+00	0.00E+00	2.44E-03
**Arabinose**	4.75E+05	4.35E+05	5.17E+05	1.59E+03	0.00E+00	0.00E+00	2.48E-03
**No match: 2686.31**	2.60E+04	2.58E+04	2.99E+04	0.00E+00	0.00E+00	0.00E+00	2.50E-03
**No match: 1047.90**	1.76E+06	1.73E+06	2.02E+06	0.00E+00	0.00E+00	0.00E+00	2.51E-03
**No match: 2172.80**	1.98E+05	1.76E+05	1.28E+05	5.42E+04	2.44E+04	0.00E+00	2.55E-03
**Lyxose**	9.09E+05	8.45E+05	1.01E+06	0.00E+00	9.40E+02	0.00E+00	2.61E-03
**No match: 1973.19**	3.90E+06	3.50E+06	4.17E+06	1.57E+04	3.09E+04	7.16E+03	2.79E-03
**No match: 2443.22**	2.82E+04	2.54E+04	3.08E+04	0.00E+00	0.00E+00	0.00E+00	3.02E-03
**Lyxose**	2.60E+05	2.52E+05	3.02E+05	0.00E+00	0.00E+00	0.00E+00	3.16E-03
**No match: 1622.53**	3.03E+05	3.08E+05	3.56E+05	3.81E+03	1.08E+04	0.00E+00	3.63E-03

**No match: 1203.72**	7.91E+03	7.83E+03	1.27E+04	3.35E+04	3.43E+04	3.43E+04	3.64E-03
**No match: 1495.37**	2.36E+04	2.72E+04	2.19E+04	0.00E+00	0.00E+00	0.00E+00	4.10E-03
**No match: 1002.51**	1.40E+07	1.57E+07	1.75E+07	0.00E+00	0.00E+00	0.00E+00	4.10E-03
**No match: 1505.32**	2.24E+04	2.80E+04	2.47E+04	0.00E+00	0.00E+00	0.00E+00	4.20E-03
**No match: 1936.61**	2.43E+05	2.32E+05	2.87E+05	0.00E+00	0.00E+00	0.00E+00	4.24E-03
**Fructose**	3.69E+07	4.22E+07	4.66E+07	4.02E+04	6.43E+03	2.82E+04	4.39E-03
**No match: 1882.04**	3.31E+05	3.37E+05	2.44E+05	1.06E+05	1.16E+05	6.47E+04	4.95E-03
**No match: 1445.53**	7.37E+04	6.89E+04	8.72E+04	0.00E+00	0.00E+00	0.00E+00	5.07E-03
**No match: 1810.06**	2.94E+07	3.50E+07	3.76E+07	3.21E+04	0.00E+00	2.55E+04	5.09E-03
**Fructose**	2.52E+07	3.02E+07	3.25E+07	1.98E+04	0.00E+00	1.87E+04	5.27E-03
**No match: 1667.90**	4.47E+04	3.64E+04	4.49E+04	0.00E+00	8.41E+02	0.00E+00	5.45E-03
**No match: 1125.27**	2.17E+06	1.96E+06	2.54E+06	4.07E+03	0.00E+00	8.38E+02	5.82E-03
**No match: 1301.30**	4.14E+04	4.96E+04	4.17E+04	5.02E+03	2.32E+03	1.76E+03	6.02E-03
**No match: 3381.26**	1.30E+05	1.54E+05	1.75E+05	0.00E+00	0.00E+00	3.71E+03	6.19E-03
**No match: 2482.09**	2.88E+04	2.46E+04	2.19E+04	0.00E+00	0.00E+00	0.00E+00	6.21E-03
**No match: 1657.58**	8.16E+05	9.92E+05	1.06E+06	5.77E+04	7.31E+04	5.11E+04	6.60E-03
**No match: 3987.10**	0.00E+00	0.00E+00	0.00E+00	3.78E+03	4.00E+03	3.03E+03	6.67E-03
**No match: 1437.24**	1.20E+06	1.15E+06	8.71E+05	1.40E+05	0.00E+00	5.89E+03	6.76E-03
**No match: 1705.97**	8.73E+04	8.24E+04	1.07E+05	0.00E+00	0.00E+00	0.00E+00	6.76E-03
**No match: 1560.78**	8.80E+04	9.91E+04	1.04E+05	2.16E+04	1.25E+04	1.80E+04	6.99E-03
**No match: 1570.57**	1.96E+04	1.90E+04	1.54E+04	1.78E+03	2.17E+03	2.11E+03	7.39E-03
**No match: 1658.13**	8.55E+05	7.61E+05	9.43E+05	0.00E+00	6.78E+04	0.00E+00	7.69E-03
**No match: 1396.71**	2.13E+05	2.25E+05	2.70E+05	2.79E+04	2.30E+04	1.89E+04	8.59E-03
**No match: 1389.34**	4.70E+04	5.81E+04	6.44E+04	6.59E+02	0.00E+00	0.00E+00	8.69E-03
**No match: 1366.52**	3.32E+04	3.48E+04	4.40E+04	0.00E+00	1.02E+03	0.00E+00	8.86E-03
**No match: 1003.87**	3.68E+05	5.09E+05	4.23E+05	0.00E+00	0.00E+00	0.00E+00	8.88E-03
**No match: 1604.75**	3.03E+05	3.06E+05	4.02E+05	6.48E+02	0.00E+00	2.09E+03	8.94E-03
**No match: 2028.75**	9.52E+04	8.13E+04	1.14E+05	0.00E+00	0.00E+00	0.00E+00	9.28E-03
**No match: 2022.86**	1.06E+06	1.27E+06	1.47E+06	3.20E+04	8.39E+04	2.98E+04	9.46E-03
**No match: 1888.53**	3.33E+04	3.97E+04	4.85E+04	6.36E+03	3.34E+03	1.11E+04	9.75E-03
**No match: 1693.92**	5.40E+06	5.32E+06	7.08E+06	4.68E+03	8.37E+04	0.00E+00	9.83E-03
**No match: 1054.48**	4.55E+05	6.01E+05	6.48E+05	0.00E+00	0.00E+00	0.00E+00	1.03E-02
**Glycerol**	3.46E+08	2.51E+08	2.64E+08	1.13E+06	1.48E+05	1.16E+05	1.03E-02
**No match: 1350.74**	1.72E+05	1.95E+05	1.45E+05	7.75E+03	0.00E+00	9.00E+03	1.04E-02
**No match: 2520.76**	4.03E+05	2.95E+05	4.22E+05	6.65E+03	0.00E+00	0.00E+00	1.07E-02
**No match: 1249.16**	2.51E+05	2.05E+05	2.84E+05	1.72E+04	2.31E+04	2.17E+04	1.08E-02
**No match: 1308.63**	1.79E+05	2.05E+05	1.82E+05	3.67E+04	4.07E+03	2.21E+04	1.09E-02
**Leucine**	1.84E+05	1.58E+05	2.25E+05	0.00E+00	1.91E+03	0.00E+00	1.10E-02
**No match: 1289.60**	1.20E+05	1.17E+05	1.75E+05	0.00E+00	2.02E+03	1.44E+04	1.19E-02
**Isoleucine**	2.03E+05	1.77E+05	2.57E+05	7.14E+03	2.68E+03	6.16E+03	1.19E-02
**No match: 2629.75**	2.70E+04	2.60E+04	3.63E+04	0.00E+00	0.00E+00	0.00E+00	1.19E-02
**No match: 2590.17**	2.28E+05	2.81E+05	3.36E+05	0.00E+00	0.00E+00	0.00E+00	1.21E-02
**Galactose**	4.09E+05	5.04E+05	6.07E+05	3.74E+03	0.00E+00	3.82E+03	1.26E-02
**No match: 2510.90**	5.72E+05	3.92E+05	4.44E+05	0.00E+00	0.00E+00	0.00E+00	1.27E-02
**No match: 1898.87**	2.54E+04	3.97E+04	2.68E+04	0.00E+00	3.41E+03	0.00E+00	1.31E-02
**No match: 1909.62**	8.12E+04	8.86E+04	1.02E+05	0.00E+00	2.36E+04	3.29E+03	1.37E-02

**Lactic acid**	1.96E+07	1.84E+07	2.66E+07	1.26E+05	1.99E+04	5.91E+04	1.39E-02
**Glycolic acid**	2.71E+06	2.54E+06	3.69E+06	5.85E+04	9.79E+03	3.84E+04	1.43E-02
**No match: 2578.39**	2.20E+03	2.16E+03	1.43E+03	1.33E+04	1.49E+04	9.71E+03	1.46E-02
**No match: 1155.19**	7.68E+04	7.25E+04	8.54E+04	2.77E+04	3.70E+04	3.06E+04	1.50E-02
**No match: 2937.35**	3.32E+04	2.17E+04	3.16E+04	0.00E+00	0.00E+00	0.00E+00	1.52E-02
**No match: 2058.53**	1.38E+04	1.09E+04	1.70E+04	0.00E+00	0.00E+00	0.00E+00	1.57E-02
**No match: 2577.97**	1.54E+05	1.25E+05	1.93E+05	0.00E+00	1.05E+03	6.76E+02	1.58E-02
**No match: 1725.47**	1.40E+05	1.61E+05	2.13E+05	0.00E+00	0.00E+00	0.00E+00	1.59E-02
**No match: 2560.29**	5.24E+05	8.18E+05	6.48E+05	0.00E+00	0.00E+00	0.00E+00	1.61E-02
**No match: 2955.81**	0.00E+00	0.00E+00	1.28E+03	1.09E+04	1.07E+04	8.37E+03	1.62E-02
**Valine**	9.01E+04	8.08E+04	1.23E+05	0.00E+00	0.00E+00	0.00E+00	1.70E-02
**No match: 1419.85**	2.52E+04	3.46E+04	4.23E+04	0.00E+00	6.62E+02	1.66E+03	1.75E-02
**No match: 2824.20**	1.37E+04	1.71E+04	2.20E+04	0.00E+00	0.00E+00	0.00E+00	1.79E-02
**Sucrose**	1.92E+05	2.56E+05	2.64E+05	4.98E+04	3.61E+04	3.68E+04	1.85E-02
**No match: 1030.08**	4.03E+05	3.27E+05	5.16E+05	2.14E+04	1.96E+04	2.03E+04	1.87E-02
**Fumaric acid**	5.96E+04	4.85E+04	8.24E+04	0.00E+00	0.00E+00	0.00E+00	2.39E-02

Supplementary Table 6. Gene expression analysis of the genes exhibiting differential expression in Caco-2 cells co-cultured with a consortium of LGG and *B. caccae* grown under anaerobic conditions in comparison with their corresponding controls. The table highlights the top 100 differentially expressed genes ordered according to their pi-values. Additionally, 3 genes which exhibit altered transcriptional response in line with earlier findings *in vivo* are also included. The threshold parameters used were FC > 1.5 and $P < 0.05$ (B*t*S).

Rank	Symbol	logFC	P.Value
**1**	SNORA31	2.370	1.56E-04
**2**	SNORA38	2.800	6.05E-04
**3**	SNORA38	2.800	6.05E-04
**4**	SNORA71B	1.658	5.42E-06
**5**	SCARNA4	2.506	4.11E-04
**6**	SNORA38	2.596	1.27E-03
**7**	SNORA38	2.596	1.27E-03
**8**	SNORA38	2.596	1.27E-03
**9**	SNORA38	2.596	1.27E-03
**10**	SNORA38	2.596	1.27E-03
**11**	SNORA38B	2.458	1.12E-03
**12**	SNORA46	2.202	5.68E-04
**13**	SNORA14B	2.408	1.08E-03
**14**	SCARNA8	2.576	2.31E-03
**15**	SNORA74A	1.972	9.48E-04
**16**	SNORD1A	1.937	9.07E-04
**17**	CPS1	-1.399	7.43E-05
**18**	SNORA34	1.258	6.69E-05
**19**	SNORA28	2.190	4.44E-03
**20**	SNORD62B	1.985	2.70E-03
**21**	SNORD62B	1.985	2.70E-03
**22**	RNU6-61	0.905	4.54E-06
**23**	ALDH6A1	-0.907	5.65E-06
**24**	RNU6-32	1.186	1.36E-04
**25**	RNU6-13	1.186	1.36E-04
**26**	RNU6-12	1.186	1.36E-04
**27**	RNU6-59P	1.449	7.11E-04
**28**	SNORA53	1.516	1.13E-03
**29**	CEACAM5	-1.096	1.16E-04
**30**	SNORA11	1.799	4.94E-03
**31**	RNU6-18	1.123	2.05E-04
**32**	RNU6-17	1.123	2.05E-04
**33**	MIR4737	-0.889	2.21E-05
**34**	LCT	-1.089	1.74E-04
**35**	SNORA10	1.541	2.28E-03
**36**	RNU6-39P	1.091	1.94E-04

**37**	SNORA72	1.745	4.81E-03
**38**	RNU6-1	1.051	1.86E-04
**39**	RNU6-37	1.053	2.12E-04
**40**	RNU6-1	1.053	2.12E-04
**41**	RNU6-1	1.053	2.12E-04
**42**	RNU6-11	1.053	2.12E-04
**43**	RNU6-1	1.053	2.12E-04
**44**	RNU6-1	1.053	2.12E-04
**45**	RNU6-1	1.053	2.12E-04
**46**	RNU6-1	1.053	2.12E-04
**47**	RNU6-1	1.053	2.12E-04
**48**	RNU6-1	1.053	2.12E-04
**49**	RNU6-33P	1.052	2.10E-04
**50**	MIR1184-2	-0.989	1.33E-04
**51**	MIR1184-1	-0.989	1.33E-04
**52**	MIR1184-2	-0.989	1.33E-04
**53**	GPC3	-0.885	4.71E-05
**54**	RNU6-24	1.072	2.68E-04
**55**	RNU6-30P	0.938	8.57E-05
**56**	RNU6-19P	0.952	9.87E-05
**57**	RNU1-13P	1.143	4.59E-04
**58**	RNU1-12P	1.143	4.59E-04
**59**	BACE1-AS	-1.255	1.06E-03
**60**	PLTP	-0.925	9.15E-05
**61**	PNPO	-1.018	2.26E-04
**62**	HPS3	-0.764	1.40E-05
**63**	RNU6-15P	0.967	1.78E-04
**64**	SNORA26	1.668	6.83E-03
**65**	ITIH2	-0.966	1.83E-04
**66**	VPS52	-0.800	3.08E-05
**67**	RNU1-18P	0.949	1.61E-04
**68**	RNU6-80	0.809	3.55E-05
**69**	SNORD83A	1.697	7.57E-03
**70**	SCARNA18	1.449	3.37E-03
**71**	OTTHUMG00000032525	-1.189	1.15E-03
**72**	RNU6-42P	0.948	2.12E-04
**73**	SNORD66	1.729	9.73E-03
**74**	RNU6-34P	1.081	6.15E-04
**75**	RNU6-4	1.012	3.96E-04
**76**	SNORD97	1.001	3.90E-04
**77**	IFI30	-0.967	3.09E-04
**78**	SNORA14A	1.548	6.51E-03
**79**	SNORD51	1.336	2.97E-03
**80**	RNU6V	0.905	2.36E-04
**81**	FMOD	-0.808	8.84E-05
**82**	RNU6-36P	0.954	3.71E-04

**83**	LOC100129034	-0.991	5.88E-04
**84**	SNORA52	1.500	7.97E-03
**85**	SNORA42	1.451	6.83E-03
**86**	RNU6-23P	0.911	3.78E-04
**87**	SCARNA7	0.994	7.83E-04
**88**	MIR3140	1.144	2.01E-03
**89**	RNU4ATAC	1.067	1.29E-03
**90**	SNORD17	1.157	2.34E-03
**91**	PTPLAD1	-0.881	3.52E-04
**92**	RNU6-45P	0.891	4.30E-04
**93**	SNORA5A	1.436	8.16E-03
**94**	PAH	-0.878	3.86E-04
**95**	RNA5SP440	0.950	7.09E-04
**96**	MIR520E	0.736	9.37E-05
**97**	SNORD94	0.998	1.08E-03
**98**	FAM127B	-0.798	2.21E-04
**99**	NOX1	-0.925	7.11E-04
**100**	RNU6-16P	0.877	4.98E-04
**101**	NDRG3	-0.611	1.34E-03
**102**	HMGCS2	-0.751	1.81E-02
**103**	CYR61	1.081	7.54E-02

Supplementary Table 7. Metabolomic analysis of the intracellular metabolites of LGG and *B. caccae* grown under anaerobic conditions following 24 h co-culture with Caco-2. The table compares the concentration of the top 100 metabolites in control and co-culture experiments ordered according to their p-values. The threshold parameters used were $P < 0.05$ (S*t*T).

Metabolite	Anaerobic co-culture with LGG and *B. caccae*			Bacteria- free Controls			p-value
**No match: 1092.01**	0.00E+00	1.47E+04	1.10E+04	1.32E+05	1.44E+05	1.42E+05	4.73E-05
**No match: 1411.30**	1.94E+05	2.12E+05	2.16E+05	2.30E+03	3.18E+03	1.75E+03	1.09E-03
**Succinic acid**	2.08E+06	2.78E+06	2.34E+06	2.97E+05	7.82E+05	6.11E+05	2.01E-03
**Lactic acid**	1.28E+08	1.15E+08	1.10E+08	7.17E+04	1.02E+06	1.55E+06	2.33E-03
**No match: 1962.10**	0.00E+00	0.00E+00	0.00E+00	9.40E+04	1.08E+05	8.76E+04	4.06E-03
**No match: 1465.76**	1.60E+06	1.03E+06	1.34E+06	2.42E+05	5.57E+04	6.63E+04	9.33E-03
**No match: 1204.70**	9.00E+03	3.11E+03	4.54E+03	2.04E+04	1.94E+04	2.01E+04	1.10E-02
**No match: 1046.09**	1.95E+06	1.47E+06	1.40E+06	2.24E+04	1.93E+03	5.09E+03	1.11E-02
**No match: 1252.85**	2.51E+05	2.97E+05	3.06E+05	7.48E+04	5.46E+04	3.06E+04	1.54E-02
**2-Hydroxybutyric acid**	1.95E+06	1.59E+06	2.29E+06	3.12E+05	1.69E+05	1.31E+05	1.55E-02
**No match: 1361.77**	3.24E+04	4.41E+04	6.56E+04	1.07E+05	1.12E+05	1.10E+05	2.13E-02
**No match: 2702.32**	0.00E+00	0.00E+00	0.00E+00	1.02E+05	1.58E+05	1.90E+05	2.84E-02
**No match: 1170.08**	0.00E+00	0.00E+00	0.00E+00	5.14E+04	9.35E+04	9.07E+04	2.86E-02
**No match: 2578.85**	0.00E+00	2.35E+04	0.00E+00	9.38E+04	1.19E+05	1.58E+05	3.19E-02
**2-Oxoglutaric acid**	4.28E+05	8.58E+05	6.57E+05	7.80E+04	1.76E+05	1.11E+05	3.19E-02
**No match: 2769.43**	0.00E+00	0.00E+00	0.00E+00	4.33E+04	6.57E+04	8.63E+04	3.45E-02
**No match: 2534.86**	6.44E+05	3.49E+05	4.26E+05	7.11E+03	2.66E+04	5.08E+04	4.48E-02
**No match: 1435.90**	1.51E+05	5.34E+04	8.83E+04	4.24E+04	5.60E+03	6.79E+03	4.53E-02
**No match: 2321.82**	1.52E+06	6.90E+05	1.51E+06	9.14E+03	1.54E+04	7.43E+04	4.54E-02
**No match: 2548.16**	4.52E+05	2.08E+05	3.26E+05	3.13E+03	7.82E+03	1.19E+04	4.68E-02
**No match: 2299.29**	9.67E+05	5.30E+05	1.24E+06	1.63E+03	3.43E+03	1.15E+04	4.76E-02
**Glutaric acid**	0.00E+00	0.00E+00	0.00E+00	1.74E+04	2.77E+04	1.27E+04	4.89E-02
**Pyruvic acid**	6.47E+06	2.65E+06	5.85E+06	0.00E+00	2.21E+03	3.16E+03	5.21E-02
**No match: 2677.37**	1.80E+05	7.92E+04	1.83E+05	1.38E+03	3.13E+03	7.88E+02	5.27E-02
**No match: 2269.56**	1.92E+06	1.12E+06	2.66E+06	2.24E+03	8.82E+04	1.09E+05	5.28E-02
**No match: 1067.87**	5.82E+05	1.81E+05	4.01E+05	2.75E+07	6.91E+07	6.07E+07	5.54E-02
**No match: 1349.38**	1.10E+06	6.43E+05	7.74E+05	2.28E+05	2.50E+05	2.87E+05	5.66E-02
**Uracil**	7.75E+05	4.34E+05	4.61E+05	4.45E+04	1.03E+05	7.33E+04	6.08E-02
**No match: 2076.04**	4.22E+05	1.27E+06	1.06E+06	3.12E+03	0.00E+00	6.38E+03	7.02E-02
**No match: 2149.22**	4.76E+05	2.96E+05	7.21E+05	4.74E+03	1.22E+05	1.78E+05	7.30E-02
**No match: 2198.96**	2.53E+06	8.50E+05	2.39E+06	8.11E+02	5.14E+04	7.85E+04	7.44E-02
**No match: 2261.30**	4.79E+05	2.62E+05	2.60E+05	6.79E+03	6.00E+04	6.03E+04	8.40E-02
**No match: 1657.04**	2.33E+05	3.21E+05	2.27E+05	1.25E+05	3.60E+04	1.02E+05	9.19E-02
**No match: 1054.38**	9.93E+05	2.97E+05	5.63E+05	6.93E+03	7.73E+03	0.00E+00	9.42E-02
**Glucose**	1.37E+08	1.09E+08	1.86E+08	4.84E+06	7.87E+07	8.37E+07	9.91E-02
**No match: 1764.11**	6.92E+04	3.62E+04	1.65E+05	1.34E+03	8.22E+03	5.14E+04	1.06E-01
**No match: 2249.63**	7.93E+06	3.06E+06	3.94E+06	8.16E+04	4.87E+05	5.97E+05	1.08E-01
**No match: 1435.97**	2.51E+05	8.92E+04	1.63E+05	5.69E+04	3.05E+04	7.30E+04	1.08E-01
**No match: 2470.72**	9.23E+03	0.00E+00	0.00E+00	1.97E+04	2.46E+03	1.27E+04	1.11E-01
**No match: 1029.50**	1.67E+03	0.00E+00	0.00E+00	1.01E+04	3.14E+04	4.29E+04	1.12E-01
**No match: 1299.34**	1.92E+05	8.31E+04	9.93E+04	3.66E+04	3.92E+04	3.25E+04	1.22E-01
**Gluconic acid**	1.61E+06	9.68E+05	1.25E+06	2.53E+05	6.75E+05	5.56E+05	1.29E-01
**No match: 1389.40**	3.00E+05	8.58E+04	1.77E+05	9.88E+03	2.63E+04	2.75E+04	1.32E-01
**Glycine**	2.27E+04	8.81E+02	2.88E+03	5.24E+05	3.35E+06	3.85E+06	1.33E-01
**No match: 2186.78**	3.13E+05	2.43E+05	7.78E+05	3.87E+03	5.09E+04	2.61E+04	1.34E-01
**Serotonin**	3.06E+06	2.47E+06	8.92E+05	6.63E+04	6.58E+05	4.17E+05	1.37E-01
**Arginine**	1.31E+06	2.47E+05	6.82E+05	5.58E+04	1.08E+04	2.98E+04	1.37E-01
**No match: 2684.72**	0.00E+00	0.00E+00	0.00E+00	1.66E+04	4.21E+04	8.65E+04	1.41E-01
**No match: 1570.86**	1.40E+05	9.45E+04	7.34E+04	3.60E+04	5.87E+04	4.38E+04	1.42E-01
**No match: 1604.62**	1.73E+07	6.15E+06	7.39E+06	2.82E+05	1.37E+06	1.58E+06	1.43E-01
**No match: 1301.25**	2.65E+05	1.13E+05	9.22E+04	4.61E+04	3.77E+04	3.39E+04	1.47E-01
**No match: 2314.40**	9.17E+04	2.28E+04	3.92E+04	1.60E+03	2.45E+03	3.52E+03	1.48E-01

**No match: 2116.43**	2.32E+05	9.81E+04	2.30E+05	1.72E+03	7.53E+04	6.34E+04	1.50E-01
**No match: 2823.82**	6.26E+04	1.70E+04	6.74E+04	1.31E+03	1.19E+04	1.06E+04	1.50E-01
**No match: 2183.05**	6.39E+05	2.31E+05	5.62E+05	2.15E+04	1.62E+05	1.77E+05	1.54E-01
**Mannose**	1.86E+08	1.46E+08	2.36E+08	7.24E+07	1.38E+08	1.17E+08	1.57E-01
**No match: 1417.79**	9.87E+04	2.79E+04	8.86E+04	1.16E+04	2.28E+04	1.40E+04	1.61E-01
**No match: 2096.70**	8.87E+05	3.24E+05	6.46E+05	1.97E+04	2.14E+05	1.95E+05	1.62E-01
**No match: 1997.70**	1.15E+06	2.81E+05	6.82E+05	1.01E+04	1.23E+05	1.24E+05	1.62E-01
**Mannose**	6.46E+06	2.16E+06	1.85E+06	4.51E+04	1.25E+05	3.14E+05	1.65E-01
**Ornithine**	9.53E+06	2.66E+06	5.50E+06	4.58E+05	1.29E+06	1.44E+06	1.66E-01
**No match: 2631.39**	3.91E+05	3.41E+04	2.72E+05	6.45E+04	2.36E+04	1.67E+04	1.75E-01
**No match: 1660.45**	6.58E+05	2.47E+05	1.71E+05	1.15E+04	3.50E+04	4.48E+04	1.78E-01
**No match: 1426.83**	4.53E+06	1.07E+06	1.55E+06	3.30E+04	1.52E+05	1.58E+05	1.81E-01
**Mannitol**	4.13E+07	1.42E+06	3.47E+07	1.11E+06	9.95E+05	2.16E+06	1.83E-01
**No match: 1754.64**	0.00E+00	0.00E+00	0.00E+00	0.00E+00	1.70E+04	1.73E+04	1.84E-01
**Unknown cgl**	0.00E+00	0.00E+00	0.00E+00	0.00E+00	1.25E+04	1.27E+04	1.84E-01
**Octadecanoic acid**	0.00E+00	0.00E+00	0.00E+00	0.00E+00	4.58E+05	4.48E+05	1.84E-01
**No match: 2025.63**	3.87E+05	7.96E+04	3.86E+05	1.66E+04	7.89E+04	5.06E+04	1.84E-01
**No match: 1252.89**	1.41E+05	7.23E+05	2.42E+05	8.59E+03	1.44E+04	1.56E+04	1.85E-01
**No match: 2897.91**	0.00E+00	0.00E+00	0.00E+00	1.42E+04	0.00E+00	1.18E+04	1.87E-01
**No match: 1426.51**	0.00E+00	0.00E+00	0.00E+00	0.00E+00	1.50E+05	1.80E+05	1.87E-01
**No match: 1609.31**	2.73E+06	4.22E+05	1.25E+06	7.91E+03	8.21E+04	1.57E+05	1.87E-01
**No match: 2338.37**	0.00E+00	0.00E+00	0.00E+00	0.00E+00	4.10E+04	5.25E+04	1.90E-01
**No match: 2579.19**	3.77E+06	3.11E+04	3.19E+06	9.38E+03	6.66E+04	2.85E+04	1.91E-01
**Glycolic acid**	0.00E+00	0.00E+00	0.00E+00	1.26E+05	1.66E+05	0.00E+00	1.91E-01
**No match: 2143.79**	3.05E+05	1.60E+05	4.18E+05	3.99E+03	1.66E+05	1.37E+05	1.91E-01
**Sarcosine**	1.40E+06	4.95E+05	5.46E+05	1.09E+06	1.42E+07	1.45E+07	1.93E-01
**No match: 1648.19**	0.00E+00	0.00E+00	0.00E+00	0.00E+00	2.24E+04	3.07E+04	1.93E-01
**2-Hydroxyglutaric acid**	1.68E+06	6.82E+06	1.65E+06	9.67E+04	3.65E+05	1.96E+05	1.95E-01
**No match: 1562.05**	3.24E+06	9.17E+05	1.14E+06	5.06E+04	2.59E+05	3.08E+05	1.96E-01
**Fumaric acid**	3.53E+06	1.26E+06	9.89E+05	1.63E+05	3.80E+05	3.06E+05	1.98E-01
**No match: 1412.98**	0.00E+00	0.00E+00	0.00E+00	0.00E+00	5.05E+03	7.48E+03	1.98E-01
**No match: 2048.48**	4.91E+05	8.30E+04	7.11E+05	5.27E+03	9.79E+04	1.18E+05	1.99E-01
**No match: 1868.21**	3.49E+05	1.30E+05	1.56E+05	7.09E+03	7.23E+04	5.70E+04	2.01E-01
**Glutamic acid**	1.13E+08	3.52E+07	4.29E+07	3.37E+06	1.40E+07	1.47E+07	2.03E-01
**Isoleucine**	2.77E+06	8.91E+05	1.39E+06	2.74E+05	5.87E+05	5.30E+05	2.05E-01
**No match: 1536.91**	1.31E+06	1.71E+05	4.50E+05	1.14E+04	1.12E+04	1.25E+04	2.06E-01
**No match: 2409.55**	2.42E+06	1.36E+06	1.57E+06	2.42E+05	1.09E+06	8.81E+05	2.13E-01
**No match: 2281.55**	9.49E+05	2.39E+05	1.78E+05	4.62E+04	1.09E+04	2.39E+04	2.14E-01
**N-Carboxy-L-valine**	2.13E+06	3.63E+05	1.04E+06	1.84E+04	2.02E+05	2.30E+05	2.15E-01
**No match: 2136.19**	3.57E+05	9.03E+04	5.31E+05	6.64E+04	1.12E+05	1.79E+05	2.16E-01
**No match: 1030.38**	6.69E+06	1.29E+06	1.47E+06	8.80E+03	1.90E+04	1.50E+04	2.19E-01
**Galactose**	5.86E+07	1.68E+06	5.48E+07	3.62E+05	6.29E+06	6.61E+05	2.19E-01
**Leucine**	2.40E+06	7.64E+05	1.19E+06	2.36E+05	5.34E+05	4.99E+05	2.20E-01
**No match: 1940.35**	6.39E+05	1.53E+05	6.05E+05	9.50E+03	1.94E+05	1.14E+05	2.21E-01
**Valine**	1.32E+07	2.60E+06	2.69E+06	3.96E+03	4.96E+03	3.90E+03	2.22E-01
**Threonine**	1.42E+07	6.29E+06	9.84E+06	2.31E+06	5.99E+06	4.53E+06	2.24E-01
**Serine**	1.29E+07	5.06E+06	6.18E+06	1.17E+06	3.50E+06	3.07E+06	2.26E-01
**Tyrosine**	4.76E+07	1.31E+07	2.25E+07	1.47E+06	9.29E+06	7.08E+06	2.26E-01

Supplementary Table 8. Gene expression analysis of Caco-2 genes that exhibited opposite expression patterns when co-cultured with LGG grown under either anaerobic or aerobic conditions in comparison to their respective controls. The table highlights the top 100 differentially expressed genes ordered according to the expression pi-values. The threshold parameters used were FC > 1.5 and *P* < 0.05 (B*t*S).

Rank	Symbol	logFC	P.Value
1	CCL2	1.05E+00	2.26E-03
2	TNFRSF9	6.92E-01	3.94E-04
3	NAALAD2	5.78E-01	2.22E-04
4	EGR1	-1.23E+00	2.60E-02
5	BIRC3	7.75E-01	4.61E-03
6	SNORA2A	-1.21E+00	4.32E-02
7	PLCB4	5.35E-01	9.87E-04
8	OTTHUMG00000086917	4.80E-01	6.66E-04
9	HILPDA	6.17E-01	3.44E-03
10	AQP10	5.45E-01	2.26E-03
11	OTTHUMG00000162284	6.97E-01	1.02E-02
12	IGLJ5	-4.27E-01	5.70E-04
13	ABCG2	5.43E-01	3.65E-03
14	OTTHUMG00000041039	4.42E-01	1.05E-03
15	OTTHUMG00000160893	-3.67E-01	3.06E-04
16	TNFAIP3	6.60E-01	1.68E-02
17	GPC5	4.75E-01	4.55E-03
18	UBD	5.02E-01	6.41E-03
19	UBD	5.02E-01	6.41E-03
20	UBD	5.02E-01	6.41E-03
21	PCDH7	4.10E-01	2.08E-03
22	UBD	5.17E-01	8.20E-03
23	UBD	4.80E-01	5.99E-03
24	ERVH48-1	5.02E-01	7.82E-03
25	UBD	4.95E-01	7.71E-03
26	UBD	4.60E-01	5.56E-03
27	ADM	6.10E-01	2.23E-02
28	LIN7A	6.15E-01	2.31E-02
29	IGFBP7	3.48E-01	1.42E-03
30	LOC100505921	-3.38E-01	1.25E-03
31	OLR1	7.10E-01	4.17E-02
32	ANKRD37	6.08E-01	2.64E-02
33	SLC26A3	-5.67E-01	2.38E-02
34	OTTHUMG00000161670	4.75E-01	1.16E-02
35	AIM1	-4.27E-01	7.57E-03
36	CA9	4.32E-01	8.68E-03
37	DPYSL3	4.25E-01	8.11E-03
38	LRRC1	3.40E-01	2.44E-03
39	OTTHUMG00000158756	3.68E-01	4.03E-03
40	EIF5	-4.18E-01	7.80E-03

**41**	OTTHUMG00000152731	-4.75E-01	1.50E-02
**42**	PI3	-5.18E-01	2.13E-02
**43**	NUAK2	4.28E-01	9.65E-03
**44**	SLC7A9	3.10E-01	1.74E-03
**45**	DPH6	3.58E-01	4.39E-03
**46**	GBE1	4.77E-01	1.69E-02
**47**	OTTHUMG00000041437	3.77E-01	5.90E-03
**48**	SERPINB1	4.00E-01	8.13E-03
**49**	RNU4ATAC3P	3.23E-01	2.99E-03
**50**	LINC00622	-3.43E-01	4.70E-03
**51**	IMPAD1	3.37E-01	4.28E-03
**52**	IRS2	-3.58E-01	6.23E-03
**53**	ABCC1	2.48E-01	6.76E-04
**54**	MIR1278	3.83E-01	9.71E-03
**55**	MIR3941	5.40E-01	3.75E-02
**56**	SULF2	-3.28E-01	4.86E-03
**57**	SCARNA8	4.15E-01	1.49E-02
**58**	OTTHUMG00000015555	-2.70E-01	1.58E-03
**59**	IFNAR2	3.48E-01	7.68E-03
**60**	NFE2L3	4.02E-01	1.48E-02
**61**	MBNL3	2.90E-01	3.25E-03
**62**	SCGN	-3.57E-01	9.64E-03
**63**	OR4C46	2.57E-01	1.60E-03
**64**	PARL	2.68E-01	2.22E-03
**65**	ZHX2	2.92E-01	3.73E-03
**66**	OTTHUMG00000016629	-4.98E-01	4.07E-02
**67**	TTTY12	2.52E-01	1.85E-03
**68**	INSIG2	4.35E-01	2.69E-02
**69**	TMSB4XP8	3.12E-01	6.45E-03
**70**	HIST2H4B	-4.67E-01	3.46E-02
**71**	VCL	2.98E-01	5.29E-03
**72**	OTTHUMG00000016953	-2.40E-01	1.52E-03
**73**	ENKUR	2.97E-01	5.32E-03
**74**	PLB1	3.35E-01	9.98E-03
**75**	PNMA1	-3.07E-01	6.53E-03
**76**	TCEAL3-AS1	-4.97E-01	4.57E-02
**77**	MIR604	4.37E-01	3.04E-02
**78**	HCG21	3.83E-01	1.88E-02
**79**	MIR3671	-3.23E-01	9.48E-03
**80**	F5	3.07E-01	7.37E-03
**81**	PPP1R3D	-3.00E-01	6.67E-03
**82**	CHAC1	-3.25E-01	9.99E-03
**83**	RBP4	-4.07E-01	2.64E-02
**84**	MIR643	4.18E-01	2.94E-02
**85**	SLC2A1	2.43E-01	2.50E-03
**86**	UGT2B17	-3.93E-01	2.47E-02
**87**	SLC19A3	3.37E-01	1.33E-02
**88**	ELMO2	-3.62E-01	1.81E-02
**89**	RBM3	3.82E-01	2.27E-02

**90**	CSRP1	3.08E-01	9.53E-03
**91**	LOC100379224	-3.58E-01	1.90E-02
**92**	HIST2H4B	-4.48E-01	4.27E-02
**93**	OTTHUMG00000013747	-3.18E-01	1.24E-02
**94**	SLC6A6	3.30E-01	1.52E-02
**95**	CKMT1A	2.82E-01	7.50E-03
**96**	MIR4778	3.03E-01	1.11E-02
**97**	OTTHUMG00000015407	-2.32E-01	2.76E-03
**98**	OTTHUMG00000058962	2.87E-01	8.58E-03
**99**	EXPH5	-3.20E-01	1.46E-02
**100**	DEPDC7	3.80E-01	2.87E-02

Supplementary Table 9. Global altered pathway analysis (GeneGo MetaCore™ pathway analysis) of Caco-2 genes that exhibited opposite expression patterns when co-cultured with LGG grown under anaerobic or aerobic conditions in comparison to their respective controls. The highlighted pathways are the top 50 altered pathways the present opposite expression trends in response to the oxygen conditions in the microbial microchamber during co-culture. The threshold parameters used were FC > 1.5 and $P < 0.01$ (B*t*S). 'Total' depicts the total number of genes that are linked to the indicated pathway, 'In data' highlights the number of genes linked to the indicated pathway that were found to be differentially expressed, and 'FDR' presents the False Discovery Rate.

Rank	Enriched pathways	Total	In Data	FDR
1	Muscle contraction_Regulation of eNOS activity in endothelial cells	65	6	1.99E-02
2	Immune response_Substance P-stimulated expression of proinflammatory cytokines via MAPKs	43	5	1.99E-02
3	Development_IGF-1 receptor signaling	52	5	2.98E-02
4	Development_VEGF signaling via VEGFR2 - generic cascades	84	6	2.98E-02
5	IGF family signaling in colorectal cancer	60	5	3.92E-02
6	Immune response_IL-1 signaling pathway	44	4	9.21E-02
7	Development_EDNRB signaling	50	4	9.92E-02
8	Immune response_C5a signaling	50	4	9.92E-02
9	Immune response_IL-13 signaling via PI3K-ERK	50	4	9.92E-02
10	G-protein signaling_Proinsulin C-peptide signaling	52	4	1.01E-01
11	Development_Insulin, IGF-1 and TNF-alpha in brown adipocyte differentiation	53	4	1.01E-01
12	Aberrant B-Raf signaling in melanoma progression	55	4	1.04E-01
13	Regulation of lipid metabolism_Insulin regulation of glycogen metabolism	56	4	1.04E-01
14	Immune response_CD137 signaling in immune cell	29	3	1.11E-01
15	Development_Transcription factors in segregation of hepatocytic lineage	30	3	1.11E-01
16	Immune response_IL-4 - antiapoptotic action	30	3	1.11E-01
17	Cell adhesion_Chemokines and adhesion	100	5	1.11E-01
18	Influence of low doses of Arsenite on glucose uptake in adipocytes	31	3	1.11E-01
19	Cell cycle_Role of APC in cell cycle regulation	32	3	1.15E-01
20	Immune response_CD40 signaling	65	4	1.16E-01
21	Cytoskeleton remodeling_TGF, WNT and cytoskeletal remodeling	111	5	1.30E-01
22	Immune response_IL-9 signaling pathway	36	3	1.30E-01
23	Development_Role of nicotinamide in G-CSF-induced granulopoiesis	12	2	1.30E-01
24	Immune response_IL-7 signaling in T lymphocytes	38	3	1.30E-01
25	Role of Endothelin-1 in inflammation and vasoconstriction in Sickle cell disease	38	3	1.30E-01
26	Transcription_Receptor-mediated HIF regulation	39	3	1.30E-01
27	Translation _Regulation of EIF2 activity	39	3	1.30E-01
28	Blood coagulation_Blood coagulation	39	3	1.30E-01
29	Main growth factor signaling cascades in multiple myeloma cells	41	3	1.30E-01
30	Translation_Insulin regulation of translation	42	3	1.30E-01
31	Apoptosis and survival_BAD phosphorylation	42	3	1.30E-01
32	Development_Growth hormone signaling via PI3K/AKT and MAPK cascades	42	3	1.30E-01
33	Signal transduction_AKT signaling	43	3	1.30E-01
34	Chemotaxis_C5a-induced chemotaxis	43	3	1.30E-01
35	Immune response_IL-4 signaling pathway	44	3	1.30E-01

**36**	Development_Adiponectin signaling	45	3	1.30E-01
**37**	Immune response_TNF-R2 signaling pathways	45	3	1.30E-01
**38**	Development_Endothelin-1/EDNRA transactivation of EGFR	46	3	1.30E-01
**39**	Signal transduction_PTEN pathway	46	3	1.30E-01
**40**	Development_Leptin signaling via PI3K-dependent pathway	47	3	1.30E-01
**41**	Immune response_PGE2 common pathways	47	3	1.30E-01
**42**	Regulation of lipid metabolism_Insulin signaling:generic cascades	47	3	1.30E-01
**43**	Development_TGF-beta-dependent induction of EMT via MAPK	47	3	1.30E-01
**44**	Development_HGF signaling pathway	47	3	1.30E-01
**45**	Stimulation of TGF-beta signaling in lung cancer	48	3	1.30E-01
**46**	Chemotaxis_CCR1 signaling	48	3	1.30E-01
**47**	Immune response_C3a signaling	48	3	1.30E-01
**48**	Regulation of metabolism_Triiodothyronine and Thyroxine signaling	48	3	1.30E-01
**49**	Muscle contraction_Relaxin signaling pathway	48	3	1.30E-01
**50**	Regulation of lipid metabolism_Insulin regulation of fatty acid metabolism	89	4	1.30E-01

Supplementary Table 10. Gene Ontology (GO)-based pathway analysis highlighting the altered biological processes in Caco-2 cells that present opposite expression patterns when co-cultured with LGG grown under anaerobic or aerobic conditions in comparison to their respective controls. For our investigation, we only included the GO processes covered by at least 10 genes in the microarray dataset, and we then computed the median gene expression levels across the process members for each of these GO processes. The significance was ascertained using the empirical Bayes t statistic (B*t*S).

Rank	Pathway	logFC	P.Value
1	GO:0015804~neutral amino acid transport	0.310	5.27E-04
2	GO:0015175~neutral amino acid transmembrane transporter activity	0.310	5.27E-04
3	GO:0008239~dipeptidyl-peptidase activity	0.322	2.00E-03
4	GO:1902176~negative regulation of intrinsic apoptotic signaling pathway in response to oxidative stress	-0.156	3.93E-03
5	GO:0002026~regulation of the force of heart contraction	-0.180	5.54E-03
6	GO:0016805~dipeptidase activity	0.210	5.83E-03
7	GO:0042149~cellular response to glucose starvation	0.422	6.22E-03
8	GO:0019725~cellular homeostasis	0.275	6.82E-03
9	GO:0004629~phospholipase C activity	0.185	7.11E-03
10	GO:0034394~protein localization to cell surface	0.129	7.12E-03
11	GO:0030214~hyaluronan catabolic process	-0.165	8.81E-03
12	GO:0043200~response to amino acid stimulus	0.163	8.96E-03
13	GO:0070411~I-SMAD binding	0.190	9.04E-03
14	GO:0048066~developmental pigmentation	-0.128	9.89E-03
15	GO:0034122~negative regulation of toll-like receptor signaling pathway	-0.130	1.12E-02
16	GO:0043014~alpha-tubulin binding	0.177	1.21E-02
17	GO:0021756~striatum development	0.245	1.54E-02
18	GO:0006754~ATP biosynthetic process	0.227	1.59E-02
19	GO:0005719~nuclear euchromatin	-0.177	1.62E-02
20	GO:0006400~tRNA modification	0.125	1.68E-02
21	GO:0030864~cortical actin cytoskeleton	0.117	1.83E-02
22	GO:0048821~erythrocyte development	-0.163	1.91E-02
23	GO:0045579~positive regulation of B cell differentiation	-0.160	1.93E-02
24	GO:0042169~SH2 domain binding	-0.183	2.03E-02
25	GO:0060348~bone development	-0.121	2.06E-02
26	GO:0045907~positive regulation of vasoconstriction	0.303	2.11E-02
27	GO:0019955~cytokine binding	0.139	2.13E-02
28	GO:0005496~steroid binding	-0.240	2.24E-02
29	GO:0042359~vitamin D metabolic process	-0.172	2.26E-02
30	GO:0019216~regulation of lipid metabolic process	0.200	2.27E-02
31	GO:0048617~embryonic foregut morphogenesis	0.202	2.28E-02
32	GO:0051491~positive regulation of filopodium assembly	0.212	2.29E-02
33	GO:0060349~bone morphogenesis	-0.110	2.39E-02
34	GO:0045445~myoblast differentiation	0.110	2.41E-02
35	GO:0046627~negative regulation of insulin receptor signaling pathway	-0.195	2.47E-02
36	GO:0050427~3'-phosphoadenosine 5'-phosphosulfate metabolic process	-0.137	2.47E-02
37	GO:0045725~positive regulation of glycogen biosynthetic process	-0.267	2.87E-02
38	GO:0009950~dorsal/ventral axis specification	0.183	2.92E-02

**39**	GO:0070412~R-SMAD binding	0.163	3.11E-02
**40**	GO:0014912~negative regulation of smooth muscle cell migration	0.293	3.37E-02
**41**	GO:0030117~membrane coat	-0.113	3.55E-02
**42**	GO:0016831~carboxy-lyase activity	0.125	3.67E-02
**43**	GO:0006069~ethanol oxidation	-0.103	3.73E-02
**44**	GO:0044262~cellular carbohydrate metabolic process	0.133	3.75E-02
**45**	GO:0055072~iron ion homeostasis	0.105	3.88E-02
**46**	GO:0051593~response to folic acid	-0.083	3.97E-02
**47**	GO:0030014~CCR4-NOT complex	0.163	4.05E-02
**48**	GO:0032270~positive regulation of cellular protein metabolic process	-0.098	4.07E-02
**49**	GO:0010875~positive regulation of cholesterol efflux	-0.098	4.07E-02
**50**	GO:0045765~regulation of angiogenesis	0.107	4.08E-02
**51**	GO:0006865~amino acid transport	-0.188	4.11E-02
**52**	GO:0001618~virus receptor activity	0.130	4.16E-02
**53**	GO:0006654~phosphatidic acid biosynthetic process	-0.106	4.22E-02
**54**	GO:0010842~retina layer formation	-0.150	4.30E-02
**55**	GO:0048015~phosphatidylinositol-mediated signaling	-0.092	4.54E-02
**56**	GO:0045638~negative regulation of myeloid cell differentiation	0.121	4.58E-02
**57**	GO:0030890~positive regulation of B cell proliferation	-0.107	4.68E-02
**58**	GO:0032008~positive regulation of TOR signaling cascade	-0.075	4.83E-02
**59**	GO:0030971~receptor tyrosine kinase binding	-0.133	5.07E-02
**60**	GO:0003333~amino acid transmembrane transport	-0.147	5.08E-02
**61**	GO:0032436~positive regulation of proteasomal ubiquitin-dependent protein catabolic process	0.100	5.12E-02
**62**	GO:0009987~cellular process	0.093	5.14E-02
**63**	GO:0004016~adenylate cyclase activity	-0.123	5.21E-02
**64**	GO:0008080~N-acetyltransferase activity	0.130	5.26E-02
**65**	GO:0036151~phosphatidylcholine acyl-chain remodeling	-0.095	5.62E-02
**66**	GO:0042809~vitamin D receptor binding	-0.088	5.75E-02
**67**	GO:0045746~negative regulation of Notch signaling pathway	-0.114	5.84E-02
**68**	GO:0007026~negative regulation of microtubule depolymerization	-0.120	5.88E-02
**69**	GO:0001967~suckling behavior	-0.128	5.95E-02
**70**	GO:0006809~nitric oxide biosynthetic process	-0.177	6.02E-02
**71**	GO:0045930~negative regulation of mitotic cell cycle	-0.073	6.11E-02
**72**	GO:0035264~multicellular organism growth	-0.067	6.20E-02
**73**	GO:0003906~DNA-(apurinic or apyrimidinic site) lyase activity	0.092	6.26E-02
**74**	GO:0035035~histone acetyltransferase binding	-0.153	6.29E-02
**75**	GO:0016779~nucleotidyltransferase activity	-0.118	6.41E-02
**76**	GO:0019433~triglyceride catabolic process	-0.113	6.46E-02
**77**	GO:0043984~histone H4-K16 acetylation	-0.097	6.46E-02
**78**	GO:0048568~embryonic organ development	-0.097	6.48E-02
**79**	GO:0071285~cellular response to lithium ion	0.125	6.63E-02
**80**	GO:0019079~viral genome replication	0.151	6.64E-02
**81**	GO:0000976~transcription regulatory region sequence-specific DNA binding	-0.098	6.75E-02
**82**	GO:0002250~adaptive immune response	-0.110	6.76E-02
**83**	GO:0032438~melanosome organization	-0.130	6.80E-02
**84**	GO:0007159~leukocyte cell-cell adhesion	0.102	6.85E-02
**85**	GO:0044295~axonal growth cone	-0.092	6.85E-02
**86**	GO:0005978~glycogen biosynthetic process	0.188	6.88E-02
**87**	GO:0007566~embryo implantation	0.125	6.95E-02

**88**	GO:0030282~bone mineralization	-0.105	7.01E-02
**89**	GO:0046620~regulation of organ growth	-0.128	7.10E-02
**90**	GO:0050661~NADP binding	0.108	7.16E-02
**91**	GO:0008610~lipid biosynthetic process	0.098	7.18E-02
**92**	GO:0003756~protein disulfide isomerase activity	0.103	7.22E-02
**93**	GO:0010575~positive regulation vascular endothelial growth factor production	0.345	7.27E-02
**94**	GO:0048872~homeostasis of number of cells	-0.103	7.33E-02
**95**	GO:0015643~toxic substance binding	-0.231	7.42E-02
**96**	GO:0015171~amino acid transmembrane transporter activity	-0.143	7.42E-02
**97**	GO:0031122~cytoplasmic microtubule organization	0.100	7.55E-02
**98**	GO:0070059~intrinsic apoptotic signaling pathway in response to endoplasmic reticulum stress	-0.088	7.63E-02
**99**	GO:0070207~protein homotrimerization	0.165	7.67E-02
**100**	GO:0045599~negative regulation of fat cell differentiation	-0.114	7.71E-02

Supplementary Table 11. Primer sequences used for the validation of the gene expression patterns inferred from the microarray data by RT-qPCR.

Gene	Primer sequence	Gene reference number
**PI3**	Fw: tgatcgtggtggtgttcct<br>Rev: acggcctttgacagtgtctt	NM_002638.3
**EGR1**	Fw: agccctacgagcacctgac<br>Rev: ggtttggctggggtaactg	ENST00000239938.4|ENSG00000120738.6
**MT2A**	Fw: caacctgtcccgactctagc<br>Rev: catttgcactctttgcatttg	ENST00000245185.5|ENSG00000125148.5
**CCL2**	Fw: agtctctgccgcccttct<br>Rev: gtgactggggcattgattg	ENST00000225831.3|ENSG00000108691.3
**L27**	Fw: acattgacgatggcacctc<br>Rev:gcttggcgatcttcttcttg	NM_000988.3

Supplementary Table 12. Functional description of genes differentially expressed in Caco-2 cells following their HuMiX-based co-culture with LGG in comparison to *in vivo* data.

Gene	Function
**EGR1**	Transcription regulation, transcription factor activity for the regulation of cell proliferation and apoptosis[1,2], anti-cancer effect[3–5] and IL-8 suppression[6]
**CCL2**	Chemotactic factor that attracts monocytes and basophils and binds to the chemokine receptors CCR2 and CCR4[7]
**SLC9A1**	Signal transduction, regulation of pH homeostasis, cell migration, cell volume[8], and anti-inflammatory effect[9]
**UBD**	Proteasomal degradation[10], cytokine response, antimicrobial response, and apoptosis[11]
**ELF3**	*ets* family member, epithelial-specific function[12], transcriptional mediator of angiogenesis during inflammation[13], and epithelial cell differentiation[14]
**CXCR4**	Chemotaxis, cell arrest, angiogenesis, cell survival, maintenance of the epithelial barrier function[15,16], and HIV-1 co-receptor[17]
**MYBL2**	Anti-apoptopic function[18], regulation of cell cycle and transcription[19], and epithelial cell differentiation[19]
**PIM1**	Cell survival, cell proliferation, cell growth, and signal transduction[20]
**CYP1A1**	Drug metabolism[21] and xenobiotic transformation[22]
**GADD45B**	Cell growth and apoptosis[23]
**PILRB**	Receptors involved in the regulation of the immune system and cellular signaling[24,25]
**CDK9**	Cell proliferation, regulation of the cell cycle, and transcription elongation factor[26,27]
**SOX4**	Transcription factor, regulation of cell fate and apoptosis pathway, and prognostic marker in colon and gastric cancer[28,29]
**CEBPA**	Transcription factor, cell cycle regulation, and regulation of metallothioneins[30,31]
**PTGS2**	Prostaglandin biosynthesis and metabolism, inflammation, and mitogenesis[32]
**IGFBP2**	Regulation of IGF-mediated growth and developmental rates[33]
**GSTA1**	Detoxification of carcinogens, drugs, environmental toxins, and products of oxidative stress[34,35]
**CTNNB1**	Regulation of cell growth in addition to the creation and maintenance of epithelial layers[36–38]
**TPD52**	Molecular marker in human cancer, and target for immunotherapy[39,40]

Supplementary Notes:

Supplementary Note 1: Design considerations for the HuMiX device. The design of the HuMiX device was optimized for the intended functionality. The spiral channel design was chosen to ensure a representative relatively large cell culture surface area (~8 cm^2) in order to provide ample material for subsequent microscopic as well as high-throughput multi-omic analyses. The dimensions of the microchambers, the membrane pore sizes (micro- and nano-porous; Figure 1b) and the perfusion flow rates were optimized such that they allowed optimal growth conditions for human and bacterial cells. In order to provide a representative model of the gastrointestinal human-microbe interface, the distance between the human and microbial cells was maintained at 700 μm analogous to the situation in the human colon[41]. The modular design of the device allows the application of specific coatings comprising extracellular matrices to the individual membranes *prior* to device assembly in order to establish *in vivo*-like conditions in each microchamber. A collagen coating was applied to the microporous membrane to support the attachment of human epithelial cells. Similarly, the nanoporous membrane was coated with mucin (~ 38 μm thickness) to facilitate the attachment and growth of enteric microorganisms. The permeability coefficient for the diffusion of 4 kDa FITC-dextran solution from the microbial microchamber to the perfusion microchamber was 5.41 x 10^{-6} cm/s which is in the same range as measured coefficients in other studies[42]. The modular architecture of the HuMiX model allows flexibility in relation to experimental design. For example the thickness of the mucin layer can be adjusted by coating the membrane with more or less mucin depending on the section of the human gut which is to be modelled or which particular experimental question is to be addressed.

Supplementary Note 2: HuMiX-based co-cultures recapitulate *in vivo* transcriptional responses of human epithelial cells following their exposure to *Lactobacillus* spp. Following the HuMiX-based co-culture with LGG, we identified a number of genes which exhibited differential expression in the Caco-2 cells in line with earlier *in vivo* data from clinical trials human clinical trials involving the administration of LGG to healthy individuals. Of particular interest is *ccl2* (FC > 1.5, P < 0.005, B*t*S), which exhibited the most pronounced upregulation among the mRNAs identified in our experiments,

and this finding is analogous to the previous independent *in vivo* data[43] (Figure 3a and S5a,b and Table 1). Our RT-qPCR results corroborated this finding (Supplementary Figure 5b, $P = 0.029$, Student's t test, n = 3). *ccl2* has previously been found to be differentially expressed following the exposure of Caco-2 cells to live and heat-killed LGG[44] as well as after the stimulation of Caco-2 cells with another probiotic strain, i.e., *Lactobacillus acidophilus* NCFM[45,46]. Furthermore, the upregulation of *ccl2* in bladder cells after the administration of LGG has also been identified in murine studies[47]. Intriguingly, concomitant to the upregulation of *ccl2*, we also found an increase in the expression of the metallothioneins *mt2a*, *mt1x*, *mt1g*, *mt1e*, *mt1jp*, *mt1cp* and *mt1h* (all FC > 2, $P < 0.01$, B*t*S) among the top 50 differentially expressed genes identified after co-culture with LGG grown under anaerobic conditions (Figure 3a). Metallothioneins are highly conserved, low-molecular-weight, cysteine-rich proteins[48], and their expression is co-regulated by many agents, including metals, hormones, cytokines, and alkylating agents[49,50]. Among, the stimulated metallothioneins, *mt2a* has been previously associated with the negative regulation of *ccl2*-mediated inflammation and tissue injury induced by LPS stimulation or direct host-pathogen interaction in mice[51,52]. Our RT-qPCR results also corroborate the observed differences in the expression of *mt2a* (Supplementary Figure 5b). Given the known anti-inflammatory effects of LGG on Caco-2 cells[53], the apparent concomitant upregulation of both *ccl2* and *mt2a* supports the notion that the negative regulation of *ccl2* by *mt2a* may indeed be involved in the inhibition of inflammatory responses following exposure of human cells to LGG[51,52].

The overexpression of *cxcr4* (FC > 1.9, $P < 0.005$, B*t*S), which was observed in our study as well as in human clinical trials following the administration of LGG, plays an active role in the maintenance and renewal of the epithelial barrier in normal and inflamed human intestinal mucosal tissues[16,54] (Supplementary Figure 5a, Table 1 and Supplementary Table 1). However, because *cxcr4* plays a dual role in regulating immune cell activation and migration to the site of inflammation[55], further investigations are required to establish the functional significance of the upregulation of *cxcr4* in epithelial cells and to identify the exact LGG-dependent molecular cues that trigger this overexpression. In this context, we also found that *mybl2* was downregulated (FC > 0.25, $P < 0.05$, B*t*S) after the co-culture of Caco-2 with LGG, which is analogous to the expression patterns observed in human clinical trial data[54]. *mybl2* is involved in cell proliferation and the differentiation of colonic

crypt cells (Supplementary Figure 5a, Table 1)[19]. Therefore, our HuMiX-based results underpin the notion that molecular factors secreted by probiotic bacteria play beneficial roles in the regulation of immune system responses and the maintenance of the epithelial barrier.

egr1, a gene that was found to be downregulated *in vivo* following LGG administration[54], was also among the most significantly downregulated genes following the co-culture of Caco-2 cells with LGG grown under anaerobic conditions in the HuMiX model (Figure 3a and Supplementary Figure 5a, c, Table 1 and Supplementary Table 1, $FC > 2.5$, $P < 0.001$, B*t*S). The RT-qPCR results for *egr1* expression support the microarray data (Supplementary Figure 5b). Reduced *egr1* expression has been found to suppress tumor growth, proliferation and differentiation in colorectal carcinoma[56], and LGG administration has been shown to reduce the incidence of chemically induced tumors in the large bowel of rodents[57]. Interestingly, reduced expression of IL-8, which was also corroborated by cytokine profiling (Figure 3b), has been previously linked to a negative regulation of *egr1*[6,58]. *egr1* has been shown to bind to NF-κβ to form a protein complex which competitively binds to the IL-8 promoter and thereby leads to the suppression of IL-8 expression[6]. Our transcriptomic results, which highlight the downregulation of *egr1* in the colonic adenocarcinoma cell line Caco-2, support the notion that LGG exerts anti-cancer effects[3–5]. However, the exact molecular mechanisms have yet to be established. In addition, the *ubd* gene (Supplementary Figure 5a, $FC > 0.6$, $P < 0.05$, B*t*S), has been found to play key roles in antigen presentation, cytokine response, apoptosis and mitosis in epithelial cells[11]. Similar to *egr1*, *ubd* expression is positively correlated with colon and gastric cancer progression and hence widely used as a predictor of recurrence following surgery[59]. The *slc9a1* gene ($FC > 0.25$, $P < 0.05$, B*t*S), which exhibited a slightly reduced expression in our study, was previously found to exhibit a transcriptional response analogous to human mucosal tissues after the administration of LGG[43]. *slc9a1* maintains the intracellular pH and cell homeostasis through ion exchange[60,61], regulates the cell cycle[62] and exhibits anti-apoptotic properties[63]. Interestingly, *slc9a1* has been shown to promote cell survival by two mechanisms: by defending the cell volume and pH through Na^+/H^+ exchange[64] and by functioning as a scaffold in colon cancer cells for the recruitment of a signal complex that links the *slc9a1*-regulated intracellular pH and Wnt signaling[65]. Because the activation of the Wnt signaling pathway is a critical event in the development of colon cancer, *slc9a1*

has become a key target gene for anti-cancerogenic therapeutic compounds in colon cancer cells[66]. Finally, *pim1*, another gene that was identified to be upregulated after LGG administration to human subjects[43] and Caco-2 cells (FC > 1.5, $P < 0.005$, B*t*S), is a proto-oncogene used as a prognosis marker for colon cancer[67]. It has been described to regulate pathways related to cell growth[20], cell cycle progression[68] and apoptosis[69] (Supplementary Figure 5a *and* Supplementary Table 1). The upregulation of *pim1* following co-culture with LGG is interesting and further investigations are required to determine whether this is a generic response by human epithelial cells or whether this is limited to cancer-derived cells. In all of the presented results, as the gene expression profiles of the co-cultured cells were compared to mono-cultured Caco-2 cells as controls, the effects observed are attributable to the influence of the co-cultured bacteria on the Caco-2 cells. Overall, given the fact that Caco-2 cells are cancer-derived, it is important to highlight that the above results highlight the ability of the HuMiX model to facilitate studies aimed at comprehensively studying the molecular mechanisms behind suggested microbiome-cancer links.

In addition to the upregulation of genes linked to inflammatory and oncogenic processes, our transcriptomic results further highlight the induction of *cyp1a1* (Supplementary Figure 5a, FC > 1.75, $P < 0.01$, B*t*S) which was also found to be upregulated in human subjects following LGG administration[43] (Table 1). This gene is known to be activated through bacterial surface structures, such as lipoteichoic acid, or *via* Toll-like receptor 2 signaling[70]. It also plays a significant role in xenobiotic uptake and drug metabolism[22]. These results indicate that soluble bacterial factors may be involved in the governing pharmacokinetics in the GIT.

Apart from the highlighted metabolites (Supplementary Figure 5a and Table 1), we also identified an increase in the intracellular concentration of creatinine in Caco-2 cells (FC > 2.9, $P < 0.2$, S*t*T; Supplementary Table 9) when these were co-cultured with LGG grown under anaerobic conditions. Earlier reports have highlighted a decrease in the luminal as well as fecal concentrations of creatinine following the conventionalization of germ-free mice[71,72], providing further evidence that intracellular creatinine levels reflect host-microbe interactions.

Supplementary Note 3: HuMiX-based co-cultures recapitulate *in vivo* global transcriptional responses of human epithelial cells following their exposure to *Lactobacillus* spp. To interprete the effects of the co-culture between LGG growing under anaerobic conditions and epithelial cells cultured under aerobic conditions on human transcriptional pathways, we applied two *in silico* analysis approaches to our microarray datasets. First, we used the GeneGo MetaCore™ integrated software suite, which includes high-quality manually curated pathway maps and networks for cellular processes, to analyze the enrichment of differentially expressed genes in specific pathways from this up-to-date knowledge base. Using this approach we found that the top-ranked cellular processes include regulation of the cell cycle, developmental processes, cytoskeleton remodeling, immune responses and signal transduction (Supplementary Table 2, $P < 0.05$, BtS). Second, we used the Gene Ontology database to conduct a data-driven analysis of the major alterations in biological processes and pathways in the Caco-2 cells following co-culture with LGG. Using this, we isolated the pathways that exhibited significant changes in the median expression levels of the pathway members under co-culture and LGG-free conditions using only the pathways with a minimum of 10 mappable genes (Supplementary Table 3, $P < 0.05$, B*t*S). Analogous to the global pathway expression changes found within healthy human mucosa exposed to LGG[43], we also found alterations in pathways related to the interferon response, calcium signaling and ion homeostasis. Among the top-ranked pathways, we also identified pathways linked to cellular homeostasis, aminopeptidase activity, desmosomes and tight-junction complexes, which together provide support for a beneficial role of LGG in enhancing epithelial barrier function[73]. Furthermore, a decrease in the expression of genes related to inflammatory pathways (response to tumor necrosis factor and LPS) further reinforce the potential anti-inflammatory effects of LGG when co-cultured with human epithelial cells (Supplementary Table 3). In addition to the LGG strain tested in human clinical trials, the effect of the administration of three distinct *Lactobacilli* probiotic strains, namely *Lactobacilli acidophilus* L10, *Lactobacilli casei* CRL-431 and *Lactobacilli plantarum*, to human subjects has also been investigated previously[43,74]. Interestingly, the comparison of the results of our global pathway analysis (Supplementary Table 2) with the global pathways that were found to be altered in human intestinal biopsies before and after exposure to probiotic strains revealed a consistent overlap of specific pathways. Specifically, pathways

involved in cytoskeletal remodeling, regulation of immune response, apoptosis and cellular differentiation were found to be influenced by all the aforementioned *Lactobacilli*[43,54,74]. Thus, there is a clear concordance in responsive pathways in Caco-2 cells following co-culture with LGG grown under anaerobic conditions and the available *in vivo* mucosal transcriptomic data generated following the administration of different probiotic *Lactobacillus* strains to human subjects[43,54,74].

Supplementary Note 4: HuMiX-based co-cultures recapitulate transcriptional responses within the epithelium of gnotobiotic piglets. As the co-culture of Caco-2 cells with LGG grown under anaerobic conditions is analogous to primocolonization of the GIT in germ-free animals, we compared our transcriptomic results with the reported gene expression differences in gnotobiotic piglet mucosal samples after the inoculation of the piglets with LGG[75]. We identified differential expression of 8 genes in our transcriptomic data which matched the gene expression patterns in germ-free piglets following the administration of LGG (Table 1). Interestingly, *cebpa* ($P < 0.03$), which has been directly linked to the regulation of *mt2a* (Figure 3a and Supplementary Table 1, FC > 1.65, $P < 0.001$), was among the top 10 differentially expressed genes following the co-culture of Caco-2 cells with LGG grown under anaerobic conditions. Moreover, *elf3* ($P < 0.03$), which plays a role in epithelial cell differentiation, has also been linked to the regulation of CCL20 in Caco-2 cells, which further corroborates our results (Figure 3b)[76]. *elf3* is also a known direct positive regulator of *ptgs2* (FC > 1.3, $P < 0.05$, B*tS*), which has been previously shown to be induced by LGG in colonic epithelial cells[77]. Both *ptgs2* and *elf3* were found to be upregulated in our data, but the expression of *ptgs2* was distinct from the transcriptional response reported in the gnotobiotic piglet study, likely resulting from differences between a single epithelial cell type and complex epithelial tissues[75].

Supplementary Note 5: HuMiX-based co-cultures induce responses in human epithelial cells following their exposure to LGG and *B. caccae* which are distinct from their responses to LGG alone. Following the co-culture of Caco-2 cells with LGG and *B. caccae*, 3 genes (*ndrg3*, *hmgcs2*, *cyr61,* Supplementary Table 6) earlier highlighted in human clinical trials to be differentially expressed after LGG intervention were found to be differentially expressed. Interestingly, these genes

were not differentially expressed when Caco-2 cells were co-cultured with LGG alone. Down-regulated *ndrg3* (FC > 1.5, $P < 0.01$) has been linked to playing a key role in lactate-induced response to hypoxia to promote angiogenesis and cell growth[78,79]. *hmgcs2* (FC > 1.5, $P < 0.05$), primarily expressed in differentiated colonic epithelium was also down-regulated and has also been shown to be down-regulated in colon cancer[80]. *hmgcs2* regulates ketogenesis and energy metabolism[81] and has been previously highlighted to be influenced by gut microbiota[82]. Additionally, *hmgcs2* has been proposed as a prognostic marker for rectal cancer[83]. *cyr61* (FC > 2, $P < 0.08$) has been linked to probiotic-mediated apoptosis during *Salmonella* infection in chicken cacae[84] and it has also been proposed as a prognostic marker for colon cancer[85]. Additionally, *cyr61* has also been suggested to mediate angiogenesis in colitis via the Substance P[86] pathway and this inflammatory disease-linked pathway[87] was also found to be among the top differentially regulated pathways in Caco-2 cells when co-cultured with LGG growing under anaerobic conditions (Supplementary Note 6, Supplementary Table 6). These results highlight the need for exploring the combinatorial effects triggered when human cells are co-cultured with microbial consortia. The HuMiX model is ideally suited to deconvolute such effects.

Supplementary Note 6: Transcriptional responses of Caco-2 cells following co-culture with LGG grown under either aerobic or anaerobic conditions. We identified a number of genes that presented opposing expression patterns in Caco-2 cells depending on whether these had been co-cultured with LGG grown under anaerobic or aerobic conditions. In particular, we identified genes playing known roles in the regulation of inflammatory responses, maintenance and regulation of epithelial barrier function, mediation of host-microbial interactions as well as regulation of cancer-related pathways including *birc3*, *tnfaip3, tnfrsf9, lrrc1*, *igfbp7*, *slc7a9*, *slc19a3*, *irs2, pi3, sulf2* and *adm* (Figure 5a and Supplementary Figure 8 and Supplementary Table 5).

Increased expression of *birc3* (FC > 1.5, $P < 0.05$, B*t*S) was identified in Caco-2 cells co-cultured with LGG growing under anaerobic conditions. Overexpression of *birc3* has been described to prevent epithelial barrier injury caused by cytokine-induced inflammation[88]. The upregulation of the *tnfrsf9* (FC > 1.5, $P < 0.05$, B*t*S) and *tnfaip3* (FC > 1.5, $P < 0.0005$, B*t*S) genes in Caco-2 cells following their co-culture with LGG grown under anaerobic conditions is similar to the results

obtained in murine models and supports earlier findings that both genes play central roles in the regulation of the epithelial barrier and the maintenance of immune homeostasis[89,90]. Moreover, *tnfaip3* plays an active role in a number of inflammatory diseases for which host-microbial interactions have been suggested to play a central role in pathogenesis, including type 1 diabetes, rheumatoid arthritis, psoriasis, lupus and Crohn's disease[91,92].

Increased levels of the regulatory peptide ADM have been demonstrated to exert anti-inflammatory effects in animal models of Crohn's disease[93,94]. ADM has also been linked to the inhibition of proinflammatory cytokines, most notably for its ability to inhibit tumor necrosis factor-α (TNFα)[95]. Most relevantly, the ADM-encoding gene (*adm*) has been shown to stabilize gut barrier function both *in vitro* and *in vivo*[96]. Overall, we observed a significant upregulation of the *adm* gene (Supplementary Figure 8; $P < 0.05$) in Caco-2 cells only when they had been co-cultured with LGG grown under anaerobic conditions, re-emphasizing the anti-inflammatory and epithelial barrier modulatory effect of LGG when cultured under *in vivo*-like oxygen concentrations. The stimulated expression of *slc19a3,* a marker for cellular differentiation, in response to LGG growing under anaerobic conditions (Supplementary Figure 8; $P < 0.05$), provides further evidence for LGG-mediated cell differentiation and regulation of epithelial barrier function[97]. In the same context, *lrrc1* (Supplementary Figure 8; $P < 0.05$), which was found to be upregulated in LGG anaerobic conditions and downregulated under aerobic conditions, is involved in the regulation of cell polarity, which is crucial for the maintenance of epithelial architecture and homeostasis[98].

pi3, which encodes a mucosal anti-microbial peptide named elafin[99], was downregulated in Caco-2 cells following co-culture with LGG grown under anaerobic conditions, whereas the expression of the *pi3* gene remained unchanged under aerobic co-culture conditions (Supplementary Figure 8; $P < 0.05$). This contrasting expression pattern of *pi3* reinforces the notion that the co-culture of Caco-2 cells with LGG growing under anaerobic conditions elicits an exclusive transcriptional response in a number of genes that are not detectable if the Caco-2 cells are co-cultured with LGG under aerobic conditions (Figure 5b). Furthermore, *egr1* and *tnfaip3* did not exhibit any differential expression in Caco-2 cells when co-cultured with LGG growing under aerobic conditions, yet these

genes exhibited a significant differential expression when co-cultured with LGG growing under anaerobic conditions (Supplementary Figure 8). *slc7a9,* an important solute carrier that is responsible for intestinal absorptive function[100], was upregulated in Caco-2 cells following their co-culture with LGG growing under anaerobic conditions but was downregulated when both cell contingents were co-cultured under aerobic conditions, indicating that LGG appears to be more effective in eliciting responses in Caco-2 cells when LGG is grown under anaerobic conditions. Additionally, the upregulation of *slc19a3*, which is typically downregulated under the influence of pathogenic bacteria[101], suggests an anti-inflammatory response in Caco-2 cells after co-culture with LGG grown under anaerobic conditions (Supplementary Figure 8; $P < 0.05$).

Interestingly, *igfbp7*, a tumor-suppressor gene that has been found to be involved in the suppression of colorectal tumors and linked to a favorable prognosis in colorectal cancer patients[102], was upregulated when LGG was co-cultured under anaerobic conditions but downregulated under aerobic conditions. Furthermore, in the context of gastrointestinal cancers, the stimulated *slc19a3* has been suggested to represent a signal of tumor growth arrest[103]. In this context, it is interesting to note that we found the upregulation of *slc19a3* in Caco-2 cells exclusively following their co-culture with LGG grown under anaerobic conditions (Supplementary Figure 8). Downregulation of the *irs2* gene was observed in Caco-2 cells co-cultured with LGG growing under anaerobic conditions, whereas the opposite expression pattern was observed in Caco-2 cells co-cultured with aerobically growing LGG (Figure 5a). *irs2* is a known oncogene in colorectal cancer and an indicator of cancer cell growth[104,105]. Regulation of irs2 expression by probiotic *Lactobacilli* has also been highlighted previously in studies involving human subjects[43]. In this context, the expression of *sulf2*, which is known for its role in colon cancer progression, migration and invasion[106], was found to be downregulated in Caco-2 cells following their co-culture with LGG grown under anaerobic conditions, providing additional evidence for the probiotic effect of LGG in colon cancer (Figure 5a, Supplementary Figure 8 and Supplementary Table 5). The mechanisms underlying the apparent positive effects of LGG and potentially other probiotics in relation to colorectal cancer[107] remain elusive, but the HuMiX model provides an essential tool for systematically exploring these in the future.

To further define the effects of LGG on Caco-2 cells when the co-cultured bacteria were grown in two distinct oxygen conditions, a GeneGo MetaCore™ pathway enrichment analysis was conducted using only the Caco-2 genes that exhibited contrasting gene expression patterns, i.e., genes that were either up- or downregulated when co-cultured with LGG under either anaerobic or aerobic conditions relative to their respective controls (the threshold parameters used were FC > 1.5 and $P < 0.05$, B*t*S, Supplementary Table 6). Interestingly, the top two pathways that exhibited contrasting gene expression patterns (Supplementary Table 6) were linked to the regulation of signaling cascades related to eNOS (endothelial nitric oxide synthase) and substance P (see also Supplemenary Note 5), both of which play major roles in gastrointestinal peristalsis[108,109]. eNOS is expressed in the gastrointestinal tract and results in the secretion of very low concentrations of nitric oxide, which mediates the relaxation of smooth muscle and thereby plays a major role in mucosal blood flow, permeability, motility and protection[110]. In this context, it is interesting to note that probiotics, particularly *Lactobacillus rhamnosus* strains, have been found to alter gut motility *in vivo*[43,111,112], which in turn may be linked to eNOS and substance P pathway activation. Of particular interest in this context are the opposing expression patterns of G-protein signaling pathways involved in proinsulin C-peptide signaling according to LGG culture conditions (Supplementary Table 6). C-peptide is typically associated with insulin release but it has also been identified as a diagnostic marker for colorectal cancer in mucosal biopsy samples[113]. The inferred contrasting expression patterns of C-peptide signaling pathways highlight the ability of HuMiX to sustain co-cultures that trigger known responses in epithelial cells and may allow for validation of diagnostic markers identified *in vivo*. Furthermore, the enriched pathways that exhibited contrasting gene expression patterns were linked to immune response, cell cycle, cell adhesion, apoptosis, cytoskeleton remodeling, lipid metabolism regulation, signal transduction and developmental signaling pathways (Supplementary Table 6). An additional data-driven pathway analysis using the Gene Ontology database revealed that the top enriched pathways which exhibited contrasting gene expression patterns under anaerobic or aerobic conditions were related to metabolism (more specifically lipid, protein and carbohydrate metabolism), cellular homeostasis, amino acid transporters, and particularly adaptive immune responses (Supplementary Table 7). Intriguingly, among the top 100 pathways, we also found pathways linked to

nitric oxide biosynthetic process, which is analogous to the previously described eNOS pathway enrichment which may be linked to the regulation of gut peristalsis.

Overall, these additional results reinforce the importance of co-culturing human cells with bacteria growing under oxygen-conditions to elicit physiologically relevant read-outs.

Supplementary Note 7: HuMiX-based co-cultures of Caco-2 cells with LGG allow the study of uncharacterized sRNAs. In addition to the miRNA microarrays, we also evaluated the differential expression of sRNAs included in the mRNA microarrays following co-culture of Caco-2 cells with LGG grown under anaerobic conditions (Figure 3a). Consistently, vault RNAs (*vtrna1-3*) exhibited a significant downregulation following co-culture (Figure 3a and Supplementary Table 1, FC > 2.5, $P < 0.0001$, B*t*S). Intriguingly, the opposite effect has been observed following the infection of a human lymphocyte cell line with gamma-herpesviruses[114], suggesting that vault RNAs may play some role in the human cellular responses to microorganisms. Furthermore, miRNA expression analyses highlight that among the top differentially expressed miRNAs, *mir3143* and *mir4521* were upregulated, whereas *mir3115*, *mir4434*, *mir4668* and *mir3941* were downregulated (Figure 3a and Supplementary Table 1, all FC > 1.5, $P < 0.05$, B*t*S). Although the functions of these miRNAs are presently unknown, the ability to analyze the expression of miRNAs in response to different microorganisms is an important attribute of the HuMiX model and therefore represents an important research tool for the future functional characterization of these bacteria-inducible miRNAs.

Supplementary Note 8: Intracellular accumulation of GABA in Caco-2 cells following co-culture with LGG. A significant increase in the intracellular concentration of 4-aminobutanoic acid (widely known as γ-aminobutyric acid or GABA) was observed in Caco-2 cells following their co-culture with LGG grown under anaerobic conditions (Figure 3c, FC = 2.18, $P < 0.06$, S*t*T). The inhibitory neurotransmitter GABA has also been shown to be synthesized, stored and secreted by mucosal endocrine-like cells throughout the mammalian intestine[115,116]. GABA is a well-known transmitter of enteric interneurons and targets excitatory $GABA_A$ or inhibitory $GABA_B$ receptors that modulate motility and mucosal function[117]. Bravo *et al.* have recently reported the brain region-specific

overexpression of $GABA_{B1b}$ receptors following the ingestion of the probiotic strain *Lactobacillus rhamnosus* JB-1[118]. Interestingly, *L. rhamnosus* JB-1 reduces stress-induced anxiety- and depression-related behavior in wild-type mice, whereas these beneficial behavioral effects are not identifiable in vagotomized mice, identifying the vagus nerve as a major communication pathway between the GIT microbiome and the brain possibly involving the GIT-derived GABA[118]. Given its modularity and flexibility for inclusion of additional cell types, the HuMiX model is well suited for future investigations into GABA synthesis by epithelial cells and into *Lactobacillus rhamnosus*-induced expression of GABA receptors in neuronal cells.

Supplementary References

1. Liu, C., Rangnekar, V. M., Adamson, E. & Mercola, D. Suppression of growth and transformation and induction of apoptosis by EGR-1. *Cancer Gene Ther.* **5,** 3–28
2. Gitenay, D. & Baron, V. T. Is EGR1 a potential target for prostate cancer therapy? *Futur. Oncol.* **5,** 993–1003 (2009).
3. Chen, A., Xu, J. & Johnson, A. C. Curcumin inhibits human colon cancer cell growth by suppressing gene expression of epidermal growth factor receptor through reducing the activity of the transcription factor Egr-1. *Oncogene* **25,** 278–87 (2006).
4. Song, L.-J. *et al.* Gastrin inhibits a novel, pathological colon cancer signaling pathway involving EGR1, AE2, and P-ERK. *J. Mol. Med. (Berl).* **90,** 707–18 (2012).
5. Kobayashi, D. *et al.* Overexpression of early growth response-1 as a metastasis-regulatory factor in gastric cancer. *Anticancer Res.* **22,** 3963–3970 (2002).
6. Ma, J. *et al.* Targeted knockdown of EGR-1 inhibits IL-8 production and IL-8-mediated invasion of prostate cancer cells through suppressing EGR-1/NF-kappaB synergy. *J. Biol. Chem.* **284,** 34600–6 (2009).
7. Deshmane, S. L., Kremlev, S., Amini, S. & Sawaya, B. E. Monocyte chemoattractant protein-1 (MCP-1): an overview. *J. Interferon Cytokine Res.* **29,** 313–26 (2009).
8. Bobulescu, I. A., Di Sole, F. & Moe, O. W. Na+/H+ exchangers: physiology and link to hypertension and organ ischemia. *Curr. Opin. Nephrol. Hypertens.* **14,** 485–94 (2005).
9. Park, S. Y., Lee, Y. J., Cho, E. J., Shin, C. Y. & Sohn, U. D. Intrinsic resistance triggered under acid loading within normal esophageal epithelial cells: NHE1- and ROS-mediated survival. *J. Cell. Physiol.* **230,** 1503–1514 (2015).
10. Groettrup, M., Pelzer, C., Schmidtke, G. & Hofmann, K. Activating the ubiquitin family: UBA6 challenges the field. *Trends Biochem. Sci.* **33,** 230–7 (2008).
11. Zhang, D. W., Jeang, K.-T. & Lee, C. G. L. p53 negatively regulates the expression of FAT10, a gene upregulated in various cancers. *Oncogene* **25,** 2318–27 (2006).
12. Tymms, M. J. *et al.* A novel epithelial-expressed ETS gene, ELF3: human and murine cDNA

sequences, murine genomic organization, human mapping to 1q32.2 and expression in tissues and cancer. *Oncogene* **15,** 2449–62 (1997).

13. Brown, C. *et al.* ESE-1 is a novel transcriptional mediator of angiopoietin-1 expression in the setting of inflammation. *J. Biol. Chem.* **279,** 12794–803 (2004).

14. Ng, A. Y. *et al.* Inactivation of the transcription factor Elf3 in mice results in dysmorphogenesis and altered differentiation of intestinal epithelium. *Gastroenterology* **122,** 1455–66 (2002).

15. Ganju, R. K. *et al.* The alpha-chemokine, stromal cell-derived factor-1alpha, binds to the transmembrane G-protein-coupled CXCR-4 receptor and activates multiple signal transduction pathways. *J. Biol. Chem.* **273,** 23169–75 (1998).

16. Jordan, N. J. *et al.* Expression of functional CXCR4 chemokine receptors on human colonic epithelial cells. *J. Clin. Invest.* **104,** 1061–1069 (1999).

17. Feng, Y., Broder, C. C., Kennedy, P. E. & Berger, E. A. HIV-1 entry cofactor: functional cDNA cloning of a seven-transmembrane, G protein-coupled receptor. *Science* **272,** 872–7 (1996).

18. Huang, M. *et al.* Identification of genes regulated by Wnt/beta-catenin pathway and involved in apoptosis via microarray analysis. *BMC Cancer* **6,** 221 (2006).

19. Papetti, M. & Augenlicht, L. H. MYBL2, a link between proliferation and differentiation in maturing colon epithelial cells. *J. Cell. Physiol.* **226,** 785–91 (2011).

20. Wang, Z. *et al.* Pim-1: a serine/threonine kinase with a role in cell survival, proliferation, differentiation and tumorigenesis. *J. Vet. Sci.* **2,** 167–79 (2001).

21. Walsh, A. A., Szklarz, G. D. & Scott, E. E. Human cytochrome P450 1A1 structure and utility in understanding drug and xenobiotic metabolism. *J. Biol. Chem.* **288,** 12932–43 (2013).

22. Uno, S. *et al.* Oral exposure to benzo[a]pyrene in the mouse: detoxication by inducible cytochrome P450 is more important than metabolic activation. *Mol. Pharmacol.* **65,** 1225–37 (2004).

23. Liebermann, D. A. & Hoffman, B. Myeloid differentiation (MyD) primary response genes in hematopoiesis. *Oncogene* **21,** 3391–402 (2002).

24. Lu, Q. *et al.* PILRα and PILRβ have a siglec fold and provide the basis of binding to sialic acid. *Proc. Natl. Acad. Sci. U. S. A.* **111,** 8221–6 (2014).

25. Banerjee, A. *et al.* Modulation of Paired Immunoglobulin-Like Type 2 Receptor Signaling Alters the Host Response to Staphylococcus aureus-Induced Pneumonia. *Infect. Immun.* **78,** 1353–1363 (2010).

26. Krystof, V., Baumli, S. & Fürst, R. Perspective of cyclin-dependent kinase 9 (CDK9) as a drug target. *Curr. Pharm. Des.* **18,** 2883–90 (2012).

27. Garriga, J., Xie, H., Obradovic, Z. & Graña, X. Selective control of gene expression by CDK9 in human cells. *J. Cell. Physiol.* **222,** 200–8 (2010).

28. Fang, C.-L. *et al.* Clinical and prognostic association of transcription factor SOX4 in gastric cancer. *PLoS One* **7,** e52804 (2012).

29. Lin, C.-M. *et al.* Clinical and Prognostic Implications of Transcription Factor SOX4 in Patients with Colon Cancer. *PLoS One* **8,** e67128 (2013).

30. Khanna-Gupta, A. Sumoylation and the function of CCAAT enhancer binding protein alpha (C/EBP alpha). *Blood Cells. Mol. Dis.* **41,** 77–81

31. Datta, J. *et al.* Metallothionein expression is suppressed in primary human hepatocellular carcinomas and is mediated through inactivation of CCAAT/enhancer binding protein alpha by phosphatidylinositol 3-kinase signaling cascade. *Cancer Res.* **67,** 2736–46 (2007).

32. Eisinger, A. L., Prescott, S. M., Jones, D. A. & Stafforini, D. M. The role of cyclooxygenase-2 and prostaglandins in colon cancer. *Prostaglandins Other Lipid Mediat.* **82,** 147–54 (2007).

33. Baxter, R. C. IGF binding proteins in cancer: mechanistic and clinical insights. *Nat. Rev. Cancer* **14,** 329–41 (2014).

34. Russo, I., Luciani, A., De Cicco, P., Troncone, E. & Ciacci, C. Butyrate attenuates lipopolysaccharide-induced inflammation in intestinal cells and Crohn's mucosa through modulation of antioxidant defense machinery. *PLoS One* **7,** e32841 (2012).

35. Morel, F., Schulz, W. A. & Sies, H. Gene structure and regulation of expression of human glutathione S-transferases alpha. *Biol. Chem. Hoppe. Seyler.* **375,** 641–9 (1994).

36. Wijnhoven, B. P., Dinjens, W. N. & Pignatelli, M. E-cadherin-catenin cell-cell adhesion

complex and human cancer. *Br. J. Surg.* **87,** 992–1005 (2000).

37. Huber, O., Bierkamp, C. & Kemler, R. Cadherins and catenins in development. *Curr. Opin. Cell Biol.* **8,** 685–91 (1996).

38. Jankowski, J. A., Bruton, R., Shepherd, N. & Sanders, D. S. Cadherin and catenin biology represent a global mechanism for epithelial cancer progression. *Mol. Pathol.* **50,** 289–90 (1997).

39. Byrne, J. A., Frost, S., Chen, Y. & Bright, R. K. Tumor protein D52 (TPD52) and cancer-oncogene understudy or understudied oncogene? *Tumour Biol.* **35,** 7369–82 (2014).

40. Han, G., Fan, M. & Zhang, X. microRNA-218 inhibits prostate cancer cell growth and promotes apoptosis by repressing TPD52 expression. *Biochem. Biophys. Res. Commun.* **456,** 804–9 (2015).

41. McGuckin, M. A., Lindén, S. K., Sutton, P. & Florin, T. H. Mucin dynamics and enteric pathogens. *Nat. Rev. Microbiol.* **9,** 265–78 (2011).

42. Marzorati, M. *et al.* The HMI™ module: a new tool to study the Host-Microbiota Interaction in the human gastrointestinal tract in vitro. *BMC Microbiol.* **14,** 133 (2014).

43. van Baarlen, P. *et al.* Human mucosal in vivo transcriptome responses to three *lactobacilli* indicate how probiotics may modulate human cellular pathways. *Proc. Natl. Acad. Sci.* **108,** 4562–4569 (2011).

44. Fang, S.-B. *et al.* Live and heat-killed *Lactobacillus rhamnosus* GG upregulate gene expression of pro-inflammatory cytokines in 5-fluorouracil-pretreated Caco-2 cells. *Support. Care Cancer* **22,** 1647–54 (2014).

45. Li, L. *et al.* Immunoregulatory effects on Caco-2 cells and mice of exopolysaccharides isolated from *Lactobacillus acidophilus* NCFM. *Food Funct.* **5,** 3261–8 (2014).

46. Jiang, Y. *et al. Lactobacillus acidophilus* induces cytokine and chemokine production via NF-κB and p38 mitogen-activated protein kinase signaling pathways in intestinal epithelial cells. *Clin. Vaccine Immunol.* **19,** 603–8 (2012).

47. Seow, S. W., Rahmat, J. N., Bay, B. H., Lee, Y. K. & Mahendran, R. Expression of chemokine/cytokine genes and immune cell recruitment following the instillation of

Mycobacterium bovis, bacillus Calmette-Guérin or *Lactobacillus rhamnosus* strain GG in the healthy murine bladder. *Immunology* **124,** 419–27 (2008).

48. Klaassen, C. D. & Liu, J. Metallothionein transgenic and knock-out mouse models in the study of cadmium toxicity. *J. Toxicol. Sci.* **23 Suppl 2,** 97–102 (1998).
49. Andrews, G. K. Regulation of metallothionein gene expression by oxidative stress and metal ions. *Biochem. Pharmacol.* **59,** 95–104 (2000).
50. Sakurai, A. *et al.* Regulatory role of metallothionein in NF-kappaB activation. *FEBS Lett.* **455,** 55–8 (1999).
51. Inoue, K. *et al.* Role of metallothionein in coagulatory disturbance and systemic inflammation induced by lipopolysaccharide in mice. *FASEB J.* **20,** 533–535 (2006).
52. Mita, M. *et al.* Metallothionein is a crucial protective factor against *Helicobacter pylori*-induced gastric erosive lesions in a mouse model. *Am. J. Physiol. Gastrointest. Liver Physiol.* **294,** G877–G884 (2008).
53. Donato, K. a, Gareau, M. G., Wang, Y. J. J. & Sherman, P. M. *Lactobacillus rhamnosus* GG attenuates interferon-{gamma} and tumour necrosis factor-alpha-induced barrier dysfunction and pro-inflammatory signalling. *Microbiology* **156,** 3288–97 (2010).
54. Di Caro, S. *et al.* Effects of *Lactobacillus* GG on genes expression pattern in small bowel mucosa. *Dig. Liver Dis.* **37,** 320–9 (2005).
55. Werner, L., Guzner-Gur, H. & Dotan, I. Involvement of CXCR4/CXCR7/CXCL12 Interactions in Inflammatory bowel disease. *Theranostics* **3,** 40–6 (2013).
56. Myung, D.-S. *et al.* Expression of early growth response-1 in colorectal cancer and its relation to tumor cell proliferation and apoptosis. *Oncol. Rep.* **31,** 788–94 (2014).
57. Gorbach, S. L. Probiotics and gastrointestinal health. *Am. J. Gastroenterol.* **95,** S2–4 (2000).
58. Singha, B. *et al.* Proteasome inhibition increases recruitment of IκB kinase β (IKKβ), S536P-p65, and transcription factor EGR1 to interleukin-8 (IL-8) promoter, resulting in increased IL-8 production in ovarian cancer cells. *J. Biol. Chem.* **289,** 2687–700 (2014).
59. Yan, D.-W. *et al.* Ubiquitin D is correlated with colon cancer progression and predicts recurrence for stage II-III disease after curative surgery. *Br. J. Cancer* **103,** 961–9 (2010).

60. Schelling, J. R. & Abu Jawdeh, B. G. Regulation of cell survival by Na+/H+ exchanger-1. *Am. J. Physiol. Renal Physiol.* **295,** F625–32 (2008).

61. Fliegel, L. The Na+/H+ exchanger isoform 1. *Int. J. Biochem. Cell Biol.* **37,** 33–7 (2005).

62. Putney, L. K. & Barber, D. L. Na-H exchange-dependent increase in intracellular pH times G2/M entry and transition. *J. Biol. Chem.* **278,** 44645–9 (2003).

63. Wu, K. L. *et al.* The NHE1 Na+/H+ exchanger recruits ezrin/radixin/moesin proteins to regulate Akt-dependent cell survival. *J. Biol. Chem.* **279,** 26280–6 (2004).

64. Khan, S., Wu, K. L., Sedor, J. R., Abu Jawdeh, B. G. & Schelling, J. R. The NHE1 Na+/H+ exchanger regulates cell survival by activating and targeting ezrin to specific plasma membrane domains. *Cell. Mol. Biol. (Noisy-le-grand).* **52,** 115–21 (2006).

65. Serafino, A. *et al.* Anti-proliferative effect of atrial natriuretic peptide on colorectal cancer cells: evidence for an Akt-mediated cross-talk between NHE-1 activity and Wnt/β-catenin signaling. *Biochim. Biophys. Acta* **1822,** 1004–18 (2012).

66. Najdi, R., Holcombe, R. & Waterman, M. Wnt signaling and colon carcinogenesis: Beyond APC. *J. Carcinog.* **10,** 5 (2011).

67. Barderas, R. *et al.* An optimized predictor panel for colorectal cancer diagnosis based on the combination of tumor-associated antigens obtained from protein and phage microarrays. *J. Proteomics* **75,** 4647–55 (2012).

68. Bachmann, M., Hennemann, H., Xing, P. X., Hoffmann, I. & Möröy, T. The oncogenic serine/threonine kinase Pim-1 phosphorylates and inhibits the activity of Cdc25C-associated kinase 1 (C-TAK1): a novel role for Pim-1 at the G2/M cell cycle checkpoint. *J. Biol. Chem.* **279,** 48319–28 (2004).

69. Shirogane, T. *et al.* Synergistic roles for Pim-1 and c-Myc in STAT3-mediated cell cycle progression and antiapoptosis. *Immunity* **11,** 709–19 (1999).

70. Do, K. N., Fink, L. N., Jensen, T. E., Gautier, L. & Parlesak, A. TLR2 controls intestinal carcinogen detoxication by CYP1A1. *PLoS One* **7,** e32309 (2012).

71. Martin, F.-P. J. *et al.* Probiotic modulation of symbiotic gut microbial-host metabolic interactions in a humanized microbiome mouse model. *Mol. Syst. Biol.* **4,** 157 (2008).

72. Marcobal, A. *et al.* A metabolomic view of how the human gut microbiota impacts the host metabolome using humanized and gnotobiotic mice. *ISME J.* **7,** 1933–43 (2013).

73. Khan, N. & Asif, A. R. Transcriptional regulators of claudins in epithelial tight junctions. *Mediators Inflamm.* **2015,** 219843 (2015).

74. van Baarlen, P. *et al.* Differential NF-kappaB pathways induction by *Lactobacillus plantarum* in the duodenum of healthy humans correlating with immune tolerance. *Proc. Natl. Acad. Sci. U. S. A.* **106,** 2371–6 (2009).

75. Kumar, A. *et al.* In vivo gut transcriptome responses to *Lactobacillus rhamnosus* GG and *Lactobacillus acidophilus* in neonatal gnotobiotic piglets. *Gut Microbes* **5,** 152–64 (2014).

76. Kwon, J. H. *et al.* ESE-1, an enterocyte-specific Ets transcription factor, regulates MIP-3alpha gene expression in Caco-2 human colonic epithelial cells. *J. Biol. Chem.* **278,** 875–84 (2003).

77. Korhonen, R., Kosonen, O., Korpela, R. & Moilanen, E. The expression of COX2 protein induced by *Lactobacillus rhamnosus* GG, endotoxin and lipoteichoic acid in T84 epithelial cells. *Lett. Appl. Microbiol.* **39,** 19–24 (2004).

78. Lee, D. C. *et al.* A lactate-induced response to hypoxia. *Cell* **161,** 595–609 (2015).

79. Park, K. C., Lee, D. C. & Yeom, Y. Il. NDRG3-mediated lactate signaling in hypoxia. *BMB Rep.* **48,** 301–2 (2015).

80. Camarero, N. *et al.* Ketogenic HMGCS2 Is a c-Myc target gene expressed in differentiated cells of human colonic epithelium and down-regulated in colon cancer. *Mol. Cancer Res.* **4,** 645–53 (2006).

81. Helenius, T. O. *et al.* Keratin 8 absence down-regulates colonocyte HMGCS2 and modulates colonic ketogenesis and energy metabolism. *Mol. Biol. Cell* **26,** 2298–310 (2015).

82. Lei, K. *et al.* Effect of dietary supplementation of *Bacillus subtilis* B10 on biochemical and molecular parameters in the serum and liver of high-fat diet-induced obese mice. *J. Zhejiang Univ. Sci. B* **16,** 487–95 (2015).

83. Lee, Y.-E. *et al.* The prognostic impact of lipid biosynthesis-associated markers, HSD17B2 and HMGCS2, in rectal cancer treated with neoadjuvant concurrent chemoradiotherapy. *Tumour Biol.* **36,** 7675–83 (2015).

84. Higgins, S. E., Wolfenden, A. D., Tellez, G., Hargis, B. M. & Porter, T. E. Transcriptional profiling of cecal gene expression in probiotic- and *Salmonella*-challenged neonatal chicks. *Poult. Sci.* **90,** 901–13 (2011).

85. Jeong, D. *et al.* Cyr61 expression is associated with prognosis in patients with colorectal cancer. *BMC Cancer* **14,** 164 (2014).

86. Koon, H.-W. *et al.* Substance P-mediated expression of the pro-angiogenic factor CCN1 modulates the course of colitis. *Am. J. Pathol.* **173,** 400–10 (2008).

87. O'Connor, T. M. *et al.* The role of substance P in inflammatory disease. *J. Cell. Physiol.* **201,** 167–80 (2004).

88. O'Callaghan, J., Buttó, L. F., MacSharry, J., Nally, K. & O'Toole, P. W. Influence of adhesion and bacteriocin production by *Lactobacillus salivarius* on the intestinal epithelial cell transcriptional response. *Appl. Environ. Microbiol.* **78,** 5196–203 (2012).

89. Gusti, V., Bennett, K. M. & Lo, D. D. CD137 signaling enhances tight junction resistance in intestinal epithelial cells. *Physiol. Rep.* **2,** (2014).

90. Vereecke, L. *et al.* Enterocyte-specific A20 deficiency sensitizes to tumor necrosis factor-induced toxicity and experimental colitis. *J. Exp. Med.* **207,** 1513–1523 (2010).

91. Vereecke, L. *et al.* A20 controls intestinal homeostasis through cell-specific activities. *Nat. Commun.* **5,** 5103 (2014).

92. Vereecke, L., Beyaert, R. & van Loo, G. The ubiquitin-editing enzyme A20 (TNFAIP3) is a central regulator of immunopathology. *Trends Immunol.* **30,** 383–91 (2009).

93. Gonzalez-Rey, E., Fernandez-Martin, A., Chorny, A. & Delgado, M. Therapeutic effect of urocortin and adrenomedullin in a murine model of Crohn's disease. *Gut* **55,** 824–32 (2006).

94. Talero, E. *et al.* Acute and chronic responses associated with adrenomedullin administration in experimental colitis. *Peptides* **29,** 2001–12 (2008).

95. Wu, R., Zhou, M. & Wang, P. Adrenomedullin and adrenomedullin binding protein-1 downregulate TNF-α in macrophage cell line and rat Kupffer cells. *Regul. Pept.* **112,** 19–26 (2003).

96. Temmesfeld-Wollbrück, B. *et al.* Adrenomedullin reduces intestinal epithelial permeability in

vivo and in vitro. *Am. J. Physiol. Gastrointest. Liver Physiol.* **297,** G43–51 (2009).

97. Nabokina, S. M., Reidling, J. C. & Said, H. M. Differentiation-dependent up-regulation of intestinal thiamin uptake: cellular and molecular mechanisms. *J. Biol. Chem.* **280,** 32676–82 (2005).

98. Saito, H. *et al.* Lano, a novel LAP protein directly connected to MAGUK proteins in epithelial cells. *J. Biol. Chem.* **276,** 32051–5 (2001).

99. Simpson, A. J., Maxwell, A. I., Govan, J. R. W., Haslett, C. & Sallenave, J.-M. Elafin (elastase-specific inhibitor) has anti-microbial activity against Gram-positive and Gram-negative respiratory pathogens. *FEBS Lett.* **452,** 309–313 (1999).

100. Yang, H. S. *et al.* Dietary supplementation with N-carbamylglutamate increases the expression of intestinal amino acid transporters in weaned Huanjiang mini-pig piglets. *J. Anim. Sci.* **91,** 2740–8 (2013).

101. Ghosal, A., Chatterjee, N. S., Chou, T. & Said, H. M. Enterotoxigenic *Escherichia coli* infection and intestinal thiamin uptake: studies with intestinal epithelial Caco-2 monolayers. *Am. J. Physiol. Cell Physiol.* **305,** C1185–91 (2013).

102. Ruan, W. *et al.* IGFBP7 plays a potential tumor suppressor role in colorectal carcinogenesis. *Cancer Biol. Ther.* **6,** 354–9 (2007).

103. Liu, X. *et al.* Promoter hypermethylation mediates downregulation of thiamine receptor SLC19A3 in gastric cancer. *Tumour Biol.* **30,** 242–8 (2009).

104. Day, E. *et al.* IRS2 is a candidate driver oncogene on 13q34 in colorectal cancer. *Int. J. Exp. Pathol.* **94,** 203–11 (2013).

105. Zhang, Q. *et al.* Role of MicroRNA 30a Targeting Insulin Receptor Substrate 2 in Colorectal Tumorigenesis. *Mol. Cell. Biol.* **35,** 988–1000 (2015).

106. Vicente, C. M., Lima, M. A., Yates, E. A., Nader, H. B. & Toma, L. Enhanced Tumorigenic Potential of Colorectal Cancer Cells by Extracellular Sulfatases. *Mol. Cancer Res.* **13,** 510–523 (2015).

107. Escamilla, J., Lane, M. A. & Maitin, V. Cell-free supernatants from probiotic *Lactobacillus casei* and *Lactobacillus rhamnosus* GG decrease colon cancer cell invasion in vitro. *Nutr.*

Cancer **64,** 871–8 (2012).

108. Li, C., Micci, M.-A., Murthy, K. S. & Pasricha, P. J. Substance P is essential for maintaining gut muscle contractility: a novel role for coneurotransmission revealed by botulinum toxin. *Am. J. Physiol. Gastrointest. Liver Physiol.* **306,** G839–48 (2014).

109. Grider, J. R. & Murthy, K. S. Autoinhibition of endothelial nitric oxide synthase (eNOS) in gut smooth muscle by nitric oxide. *Regul. Pept.* **151,** 75–9 (2008).

110. Dijkstra, G., van Goor, H., Jansen, P. L. M. & Moshage, H. Targeting nitric oxide in the gastrointestinal tract. *Curr. Opin. Investig. Drugs* **5,** 529–36 (2004).

111. Wu, R. Y. *et al.* Spatiotemporal maps reveal regional differences in the effects on gut motility for *Lactobacillus reuteri* and *rhamnosus* strains. *Neurogastroenterol. Motil.* **25,** e205–14 (2013).

112. Williams, M. D., Ha, C. Y. & Ciorba, M. A. Probiotics as therapy in gastroenterology: a study of physician opinions and recommendations. *J. Clin. Gastroenterol.* **44,** 631–6 (2010).

113. Vidal, A. C. *et al.* Elevated C-peptide and insulin predict increased risk of colorectal adenomas in normal mucosa. *BMC Cancer* **12,** 389 (2012).

114. Nandy, C. *et al.* Epstein-barr virus-induced expression of a novel human vault RNA. *J. Mol. Biol.* **388,** 776–84 (2009).

115. Davanger, S. *et al.* Colocalization of gamma-aminobutyrate and gastrin in the rat antrum: an immunocytochemical and in situ hybridization study. *Gastroenterology* **107,** 137–48 (1994).

116. Krantis, A., Nichols, K., de Blas, A. & Staines, W. Demonstration of benzodiazepine receptors in submucosal neurons of the gastrointestinal tract. *Neurosci. Lett.* **176,** 32–6 (1994).

117. Krantis, A. GABA in the Mammalian Enteric Nervous System. *News Physiol. Sci.* **15,** 284–290 (2000).

118. Bravo, J. A. *et al.* Ingestion of *Lactobacillus* strain regulates emotional behavior and central GABA receptor expression in a mouse via the vagus nerve. *Proc. Natl. Acad. Sci.* **108,** 16050–16055 (2011).
